# Enhancing Drug Delivery Efficacy Through Bilayer Coating of Zirconium-Based Metal–Organic Frameworks: Sustained Release and Improved Chemical Stability and Cellular Uptake for Cancer Therapy

**DOI:** 10.1021/acs.chemmater.3c02954

**Published:** 2024-04-11

**Authors:** Xiewen Liu, Joanna Obacz, Giulia Emanuelli, Joseph E. Chambers, Susana Abreu, Xu Chen, Emily Linnane, Joshua P. Mehta, Andrew E. H. Wheatley, Stefan J. Marciniak, David Fairen-Jimenez

**Affiliations:** † The Adsorption & Advanced Materials Laboratory (A^2^ML), Department of Chemical Engineering & Biotechnology, University of Cambridge, Philippa Fawcett Drive, Cambridge CB3 0AS, United Kingdom; ‡ Cambridge Institute for Medical Research, Keith Peters Building, Cambridge Biomedical Campus, University of Cambridge, Cambridge CB2 0XY, United Kingdom; § Yusuf Hamied Department of Chemistry, University of Cambridge, Lensfield Road, Cambridge CB2 1EW, United Kingdom

## Abstract

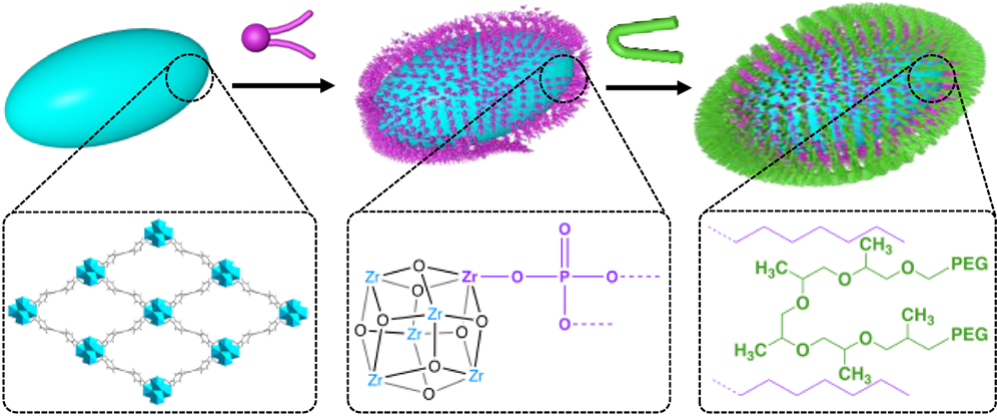

The development of nanoparticle (NP)-based drug carriers has presented an exciting opportunity to address challenges in oncology. Among the 100,000 available possibilities, zirconium-based metal–organic frameworks (MOFs) have emerged as promising candidates in biomedical applications. Zr-MOFs can be easily synthesized as small-size NPs compatible with intravenous injection, whereas the ease of decorating their external surfaces with functional groups allows for targeted treatment. Despite these benefits, Zr-MOFs suffer degradation and aggregation in real, in vivo conditions, whereas the loaded drugs will suffer the burst effect—i.e., the fast release of drugs in less than 48 h. To tackle these issues, we developed a simple but effective bilayer coating strategy in a generic, two-step process. In this work, bilayer-coated MOF NU-901 remained well dispersed in biologically relevant fluids such as buffers and cell growth media. Additionally, the coating enhances the long-term stability of drug-loaded MOFs in water by simultaneously preventing sustained leakage of the drug and aggregation of the MOF particles. We evaluated our materials for the encapsulation and transport of pemetrexed, the standard-of-care chemotherapy in mesothelioma. The bilayer coating allowed for a slowed release of pemetrexed over 7 days, superior to the typical 48 h release found in bare MOFs. This slow release and the related performance were studied in vitro using both A549 lung cancer and 3T mesothelioma cells. Using high-resolution microscopy, we found the successful uptake of bilayer-coated MOFs by the cells with an accumulation in the lysosomes. The pemetrex-loaded NU-901 was indeed cytotoxic to 3T and A549 cancer cells. Finally, we demonstrated the general approach by extending the coating strategy using two additional lipids and four surfactants. This research highlights how a simple yet effective bilayer coating provides new insights into the design of promising MOF-based drug delivery systems.

## 1 Introduction

Cancer stands as one of the most significant global health challenges, impacting millions of lives annually.^1^ Its importance stems not only from its widespread prevalence and high mortality rates but also from its complex nature, which involves genetic, environmental, and lifestyle factors. The disease’s profound impact on patients, families, and healthcare systems underscores the urgent need for innovative treatment strategies. New therapeutics, many times, require adequate formulation to make sure they arrive to tumor cells.^2^ As such, optimal and biocompatible drug delivery systems should enable controlled, sustained medication release. Indeed, an optimal system should (i) shield the active pharmaceutical ingredients (APIs) and ensure their accurate delivery to the intended site, (ii) maintain in vivo concentration to reduce the frequency of administration and mitigate adverse effects associated with systemic delivery, and (iii) guarantee the therapeutic effectiveness of the APIs upon reaching their target.^3^ Presently, the predominant delivery systems are organic-based, including various lipid formulations, hydrogels, micelles, and diverse polymeric nanocarriers.^4,5^ Despite their widespread use, these systems often suffer from limitations, such as inconsistent drug release rates and limited drug loading capacity, in the order of 5 wt %.

Metal–organic frameworks (MOFs) are porous and crystalline materials formed from the self-assembly between metal nodes and organic linkers.^6^ The diversity that MOFs offer owes to the possibility of tuning the metals and the linkers, leading to more than 100,000 MOF structures reported on the Cambridge Structural Database.^7,8^ As multifunctional materials, MOFs have been widely reported for gas storage,^9^ gas separation,^10^ catalysis,^11^ and sensing applications.^12^ Over the past decade—and thanks to the pioneering work from Horcajada, Gref, Serre, Morris, Lin et al.—MOFs have also gained traction as promising drug delivery nanocarriers.^13−16^ The high modifiability of surface functionalities and capacity for encapsulating substantial quantities of active substances^17−19^ position MOFs as a promising alternative to conventional organic carriers, enabling efficient drug delivery with reduced API quantities and minimal toxicity.

In this regard, we have recently shown that it is possible to design MOF nanocarriers that can deliver multiple, small-molecule cancer drugs,^20^ knockdown gene via delivery of small interfering RNA (siRNA),^21^ and achieve targeted delivery to the cytosol^22^ as well as to the mitochondria.^23^ We have also demonstrated the controlled release of cargoes from MOFs by collapsing the porosity around the payload, either mechanically^24^ or thermally.^18^ Through modifying the particle size and external surface chemistry of MOFs, we and others successfully directed their cellular uptake to clathrin- or caveolae-mediated endocytic.^16,17^ By using a methoxy-PEG phosphate coating, we also enhanced the colloidal stability and redispersity of MOFs—an approach that should allow for the MOF translation to the clinic.^27^ MOF toxicity has been reviewed recently, including key features that might affect their biocompatibility.^28,29^ In any case, a critical limitation for using MOFs in the clinic is the limited control of the burst effect of drugs from the porosity.^25^

Interestingly, the aqueous instability of certain MOFs, which might generally be seen as a drawback, is—in principle—advantageous in drug delivery systems as it prevents bodily accumulation, thereby reducing cytotoxicity.^24,30^ Considering the safety and stability requirements of MOFs for biomedical applications, zirconium (Zr)-based MOFs—with low toxicity and strong coordination with carboxylic linkers—are a popular choice.^31^ Despite their excellent stability in water, the stability of Zr-MOFs in the body remains an issue due to the presence of phosphate ions (PO_4_^3–^) in biological environments. With notable exceptions,^32^ phosphate groups will disassemble the Zr-based, or other metals, MOF structure by substituting carboxylic linkers to form Zr–O–P bonds. The higher affinity of phosphates toward the Zr clusters makes MOF nanoparticles lose their crystallinity and morphology.^33^ Beyond degradation, phosphate ions can also significantly affect the colloidal stability of nanoMOFs in an aqueous environment, provoking MOFs’ aggregation and impeding their cellular uptake and in vivo administration.^34^ In detail, aggregation hinders the free circulation of MOFs in blood vessels and activates clearing by phagocytes,^35^ while early degradation causes the burst-release of drugs before reaching the tumor sites.^36^ Although modifying their external surface can enhance the stability of Zr-MOFs,^27,37,38^ most grafting polymers are cytotoxic,^39^ expensive,^40^ and often involve expensive multistep synthesis.^26^ We have published elsewhere an assessment of the advancements in external surface functionalization of MOFs.^41^ All in all, these limitations present significant challenges in translating MOFs for clinical use.

**Scheme 1:**
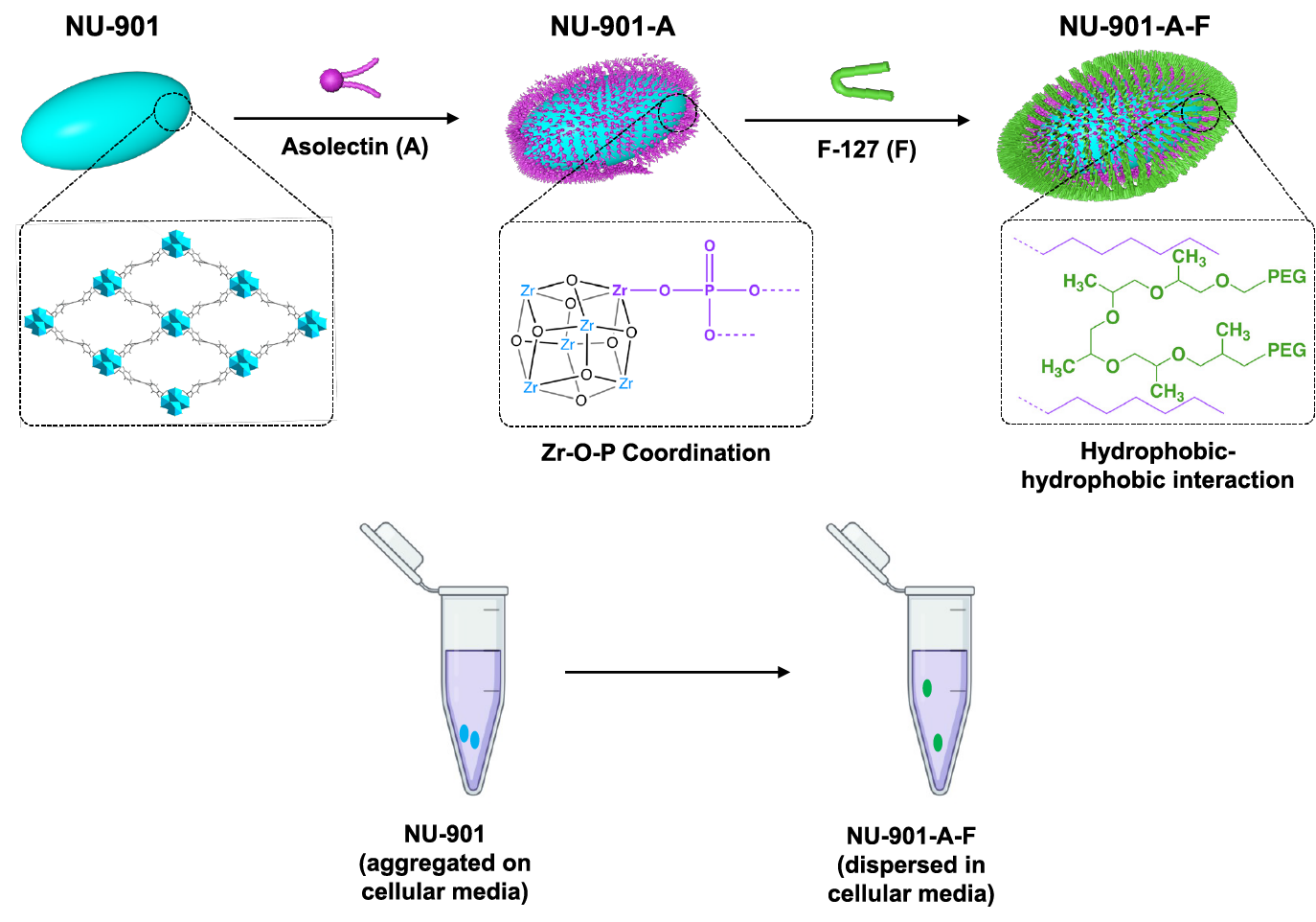
Schematic Illustration of NU-901 Coating with Asolectin and Biosurfactant F-127 to Allow Good Dispersibility in Cellular Media

Driven by these problems, we developed a simple, safe, and economical solution. Our previous PEGylation process allowed to protect the external surface of MOFs and allow for better hydrochemical and colloidal stability.^18^ Here, we design a novel bilayer coating strategy to protect MOFs from aggregation and avoid the burst release of drugs in physiological media. The decision to use a bilayer phospholipid instead of a traditional PEGylation approach for the external surface of our MOF is driven by several factors. First, phospholipids are major components of cell membranes, making them inherently biocompatible. This biomimetic approach aims to enhance the integration of our MOF system within biological environments, potentially reducing the immunogenicity often associated with foreign materials. Second, while PEG primarily offers steric stabilization, phospholipids can engage in more complex interactions with biological systems, including potential fusion with cell membranes for direct intracellular delivery. Scheme 1 shows the bilayer coating strategy. This bilayer coating is a combination of two facile methods: a solvent-assisted ligand incorporation^42^ (SALI) and emulsification,^43^ which offer double protections to MOFs. Inspired by the stability of the Zr–O–P bond,^40,44^ we used a SALI approach to graft a first layer of asolectin, an economic and biocompatible mixture of zwitterionic phospholipid extracted from soybeans,^45^ on the external surface of a MOF. We then used emulsification to incorporate a second layer of biosurfactant, Pluronic F-127, through hydrophobic–hydrophobic interactions,^46^ to inhibit the aggregation of MOFs. F-127 is an amphiphilic triblock copolymer of [poly(ethylene glycol)]–[poly(propylene glycol)]–[poly(ethylene glycol)] (PEG–PPG-PEG), where the middle PPG block is hydrophobic while both of the terminal PEG blocks are hydrophilic. More importantly, the inner hydrophobic region of the bilayer provides a continuous shield, which is particularly impermeable to phosphate ions, and therefore, we hypothesize that it will protect the existing coordination bonds of the MOF.

To probe the effectiveness of our approach, we focused on nonsmall cell lung cancer (NSCLC) and mesothelioma, two hard-to-treat cancers that urgently need novel therapies. Mesothelioma, in particular, is an aggressive cancer that commonly occurs in the linings of the pleural space^47^ and shows limited therapeutic options and a dismal prognosis of approximately 1 year,^48^ while NSCLC subtypes account for 40% of patients with lung cancer.^49^ Here, we focused on the use of pemetrexed—the standard-of-care (SoC) chemotherapeutic drug^50^ for, among others, these two cancers. Pemetrexed is a chemotherapeutic drug that inhibits the synthesis of DNA precursors, as it works as a folate antimetabolite. Pemetrexed has been the SoC for mesothelioma since it was licensed for this purpose in 2004. As its structure suggests, it is a well-known folate analogue and unequivocally acts as a folate antimetabolite, inhibiting enzymes, including thymidylate synthetase and dihydrofolate reductase. We studied two cell models: a lung cancer cell line (A549) and a primary cell line of epithelioid mesothelioma (3T) from the Mesobank due to their sensitivity to pemetrexed. We demonstrated that the bilayer coating could control the drug release from MOFs more sustainably. Furthermore, we also studied the live colocalization of bare and bilayer-coated MOF with 3T and A549 cells through z-stack super-resolution confocal microscopy imaging and flow cytometry. We found that the bilayer coating significantly improved the uptake efficiency of drug-loaded MOFs in lung cancer cell. To demonstrate the generality of our strategy, we used two additional lipids and four surfactants. Altogether, this novel bilayer coating approach indicates a simple way to increase the colloidal stability of drug delivery vehicles in the physiological environment, enhancing their potential in biomedical applications.

## 2 Size and Morphology: Controlled Synthesis of Zr-MOFs

**Figure 1:**
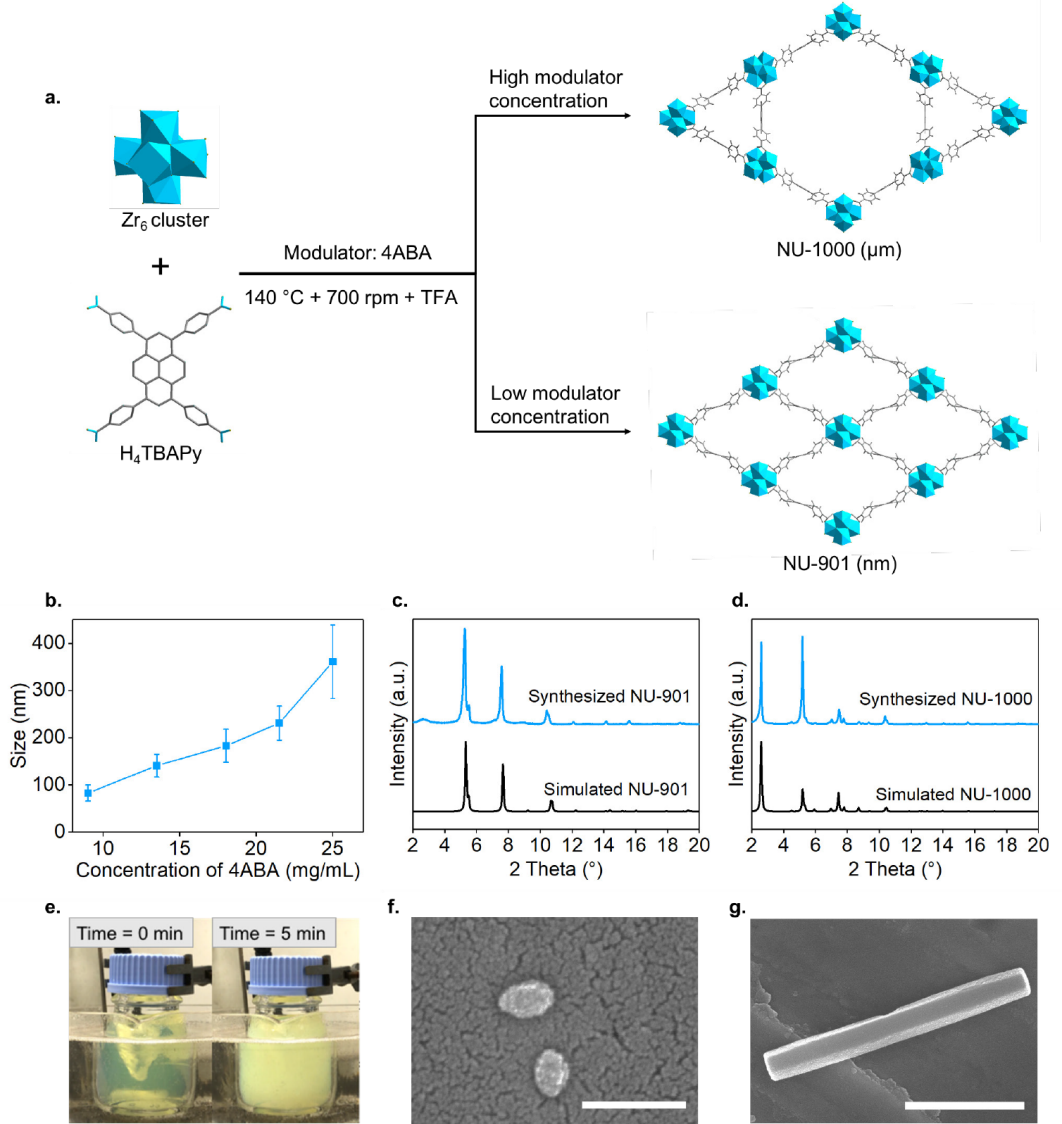
Controlled synthesis of Zr-MOFs. (a). Synthesis of NU-901 and NU-1000 with high and low concentrations of modulators, respectively. (b). Effect of the concentration of modulator 4ABA on the particle size of NU-901. (c). PXRD of as-synthesized and simulated nanoNU-901. (d). PXRD of as-synthesized and simulated microNU-1000. (e). Photograph of 5 min precipitation of NU-901 nanoparticles during synthesis. (f.) SEM of nanoNU-901 particles, scale bar = 200 nm. (g). SEM images of a microNU-1000 particle; scale bar = 1 μm.

We focused in this work on NU-901, a prototypical Zr (IV)-based MOF with large mesopores. Mesopores not only allow for the adsorption of large amounts of drugs but also macromolecules such as siRNA.^18,21^ NU-901 consists of Zr_6_ clusters connected to 1,3,6,8-tetrakis(*p*-benzoic acid) pyrene (H_4_TBAPy) linkers, featuring 1D rhombus channels with a pore size of 12 Å × 26 Å.^51,52^ By changing the synthetic conditions, the same precursors can also yield a different structure, NU-1000,^53^ in which the Zr_6_ nodes are oriented differently from the ones in NU-901.^54,55^ When synthesizing a drug delivery system, a short synthetic route and large scale that does not compromise the quality of the final materials are always preferable. To date, several established methods have shown the possibility of producing NU-901 MOF nanoparticles smaller than 200 nm. However, low crystallinity, long synthesis time, and small scale remain an issue.^18,56,57^ Here, we prepared nanosized NU-901 through a solvothermal reaction between H_4_TBAPy^58^ (Figure S1) and ZrOCl_2_·8H_2_O in dimethylformamide (DMF) using *para*-aminobenzoic acid (4ABA) as a modulator and trifluoroacetic acid (TFA) as a comodulator. Figure 1a shows the general strategy for the synthesis of NU-901 and NU-1000. We introduced a rigorous magnetic stirring at 700 rpm and 140 °C to accelerate the nucleation of the NU-901 particles with a precipitation time of only 5 min (Figure 1e). This modified method returned 100 mg of NU-901 in 80 mL and 50 min (i.e., 36 g/L/day), in contrast with previous studies where 18-h reactions were required to produce ca. 1 mg of nanosized NU-901.^18,21^

The size of a drug delivery vehicle, the MOF particles in this case, is crucial as it directly affects the cellular uptake efficiency^22,59^ and biodistribution in the body.^60,61^ As such, we studied the impact on the particle size of the synthesis time, temperature, and modulator concentration. We found both synthesis time and temperature to have minimal effects on particle size (Figure S4). On the other hand, Figure 1b shows the increment of the particle size with the 4ABA modulator concentration. Particle size increases from 90 to 320 nm when the 4ABA concentration is increased from 8 to 25 mg/mL; Figure S5 provides full details on the particle size analysis. In particular, concentrations of 4ABA under 15 mg/mL resulted in 141 ± 24 nm oval NU-901 nanoparticles (Figure 1f), but when the concentration of 4ABA increased to 60 mg/mL, the synthesis resulted in 1.9 ± 0.3 μm NU-1000 cylindrical microparticles (Figure 1g). Therefore, the lower concentration favors the nucleation of small NU-901 MOF particles, while the higher concentration leads to the formation of large NU-1000 particles. The phases were confirmed by the different particle shapes and the powder X-ray diffraction (PXRD) patterns. Figure 1c shows the PXRD for NU-901, matching the simulated pattern of the single crystal structure of NU-901, whereas the PXRD of NU-1000 (Figure 1d) shows the distinctive peaks of NU-1000 at 2.6° (1 0 0), 4.5° (2–1 0), and 6.0° (1 0 1).^52,56^ To discover the phase change behavior of NU series MOFs when using other modulators, we repeated the synthesis by replacing 4ABA with two modulators known for yielding phase-pure NU-1000:^58,62^ benzoic acid (BA) and biphenyl-4-carboxylic acid (B4CA). When using low concentrations of modulators (8 mg/mL), we obtained nanosized NU-901 of 188 ± 22 and 190 ± 23 nm in size, respectively (Figure S6). In contrast, high concentrations of BA and B4CA (80 mg/mL) yielded microsized NU-1000 particles of 1.36 ± 0.46 and 1.57 ± 0.47 μm in size, respectively (Figure S7). To the best of our knowledge, this is the first reported evidence of simultaneous size-and-phase-switching between NU-901 and NU-1000 by tuning only the concentration of modulators. Previous works induced NU-901 to NU-1000 phase change by adjusting the H_4_TBAPy linker concentration^63^ or switching the types of modulators, including 4ABA, TFA, BA, and B4CA.^64^ In addition, Figures 1f and S2 show the scanning and transmission electron microscopy (SEM and TEM) images, respectively, of the synthesized nanoparticles with their typical and expected oval shape. Figure S3a shows the N_2_ isotherms at 77 K; Figure S3b shows the pore size distribution (PSD) using the nonlocal density functional theory (NLDFT) implemented in the Micromeritics software. The central pore size is centered at 26 Å, possibly related to missing ligand defects or NU-1000 phase impurities in the nanoparticles.^52,62^ Using our BETSI protocol, the synthesized nanosized NU-901 shows a BET area of 1951 m^2^/g (see Section S5 for more details).^65^

Although the modulation strategy for controlling the particle size of Zr-MOFs has been studied before,^66^ a clear understanding of the effect of modulators on the phase change between NU-901 and NU-1000 is not present. Our proposed mechanism stems from their growth process, with NU-901 being denser than NU-1000 due to the higher packing of the Zr_6_ clusters (Figure 1a).^51,62,66^ We assume that the Zr_6_ clusters are first capped with modulators to form the clusters; this is followed by the H_4_TBAPy linker being exchanged by the modulators to promote the growth of the framework. Hence, when an excess of modulator is added, most Zr_6_ clusters are entirely capped with modulators, provoking stronger steric repulsion between them and forming the less-dense NU-1000 phase. In contrast, when using a low modulator concentration, more Zr_6_ clusters will remain uncapped or partially capped, forming the denser NU-901 phase due to weaker repulsion among clusters.

## 3 Optimization of the MOF using a Bilayer Coating

**Figure 2:**
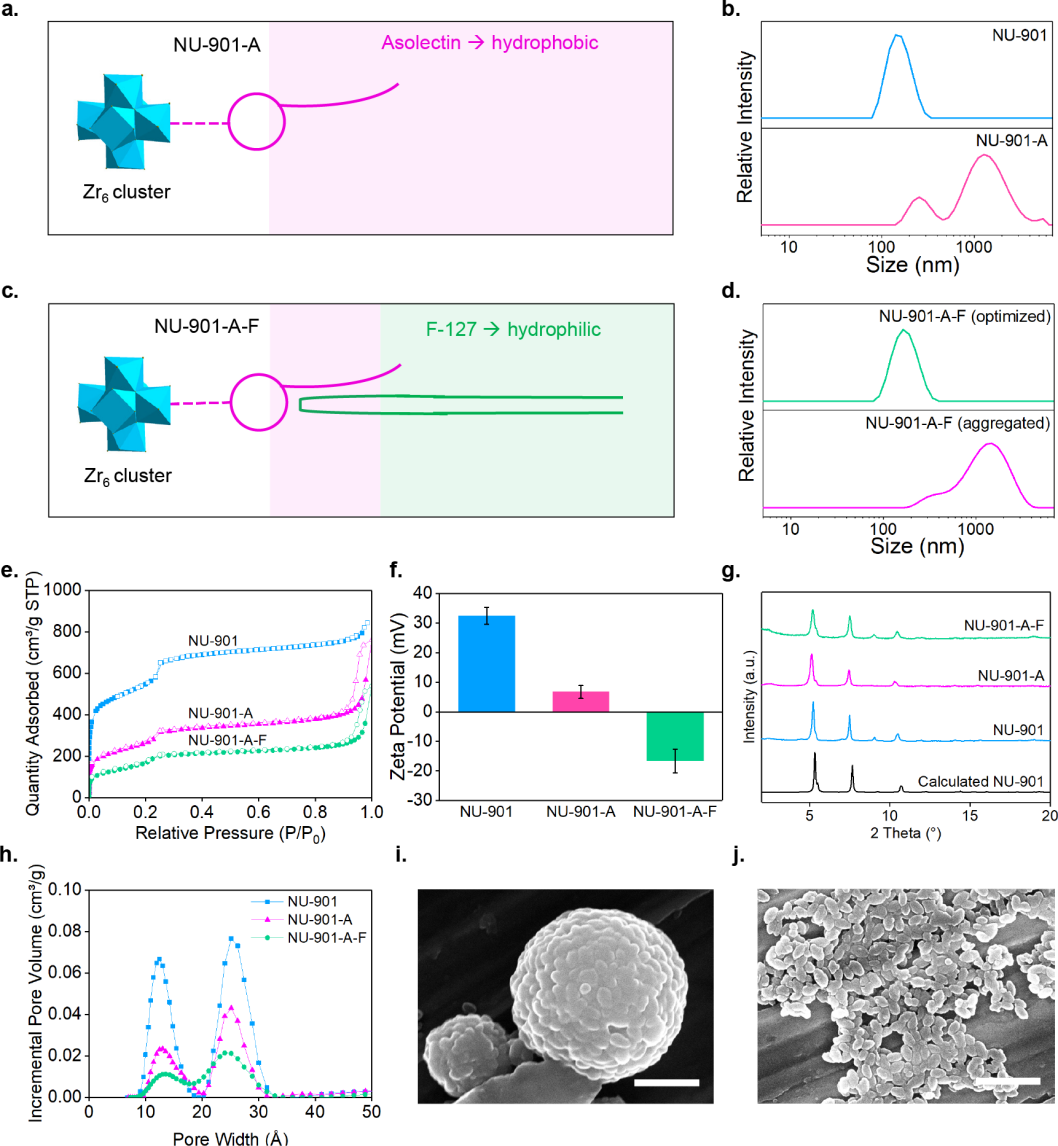
External surface modification of NU-901. (a). Schematic illustration of coating NU-901 with asolectin to induce hydrophobicity. (b). Intensity-averaged DLS size of the suspension of NU-901 (blue line) and NU-901-A (purple line) in water. (c). Schematic illustration of coating NU-901-A with F-127 to induce hydrophilicity. (d). Intensity-average size of the aggregated and optimized NU-901-A-F in water. (e). N_2_ isotherms of NU-901, NU-901-A, and NU-901-A-F at 77 K. (f). Zeta potentials of NU-901, NU-901-A, and NU-901-A-F. (g). PXRD patterns of synthesized NU-901, NU-901-A, and NU-901-A-F with calculated NU-901. (h). Pore size distributions obtained with the NLDFT method; SEM images of (i). severely aggregated and (j). well dispersed NU-901-A-F particles; scale bars = 1 μm.

After controlling the particle size, we applied the bilayer coating of NU-901 in a two-step fashion. In the first step, we mixed the zwitterionic asolectin (A, with its chemical structure shown in Figure S8) with nanosized NU-901 (141 ± 24 nm) suspension in chloroform (CHCl_3_) for 4 h at room temperature to form **NU-901**-**A** (Figure 2a) owing to Zr–O–P bonding.^40^ Here, both the crystallinity and morphology of the nanoparticles remained intact: Figure 2g shows the resulting PXRD after the addition of asolectin, whereas Figure S8 shows the SEM images. Figure S9 shows the Fourier- transform infrared (FT-IR) spectra, with the addition of new bands at 2837 and 2854 cm^–1^, attributed to the stretching vibration of _symmetric_CH_2_ and _asymmetric_CH_2_, respectively, from asolectin, confirming the successful grafting. Considering the weight percentage of phosphorus (P) present in **NU-901**-**A**, inductively coupled plasma-optical emission spectroscopy (ICP-OES) confirmed 16.8 wt % of asolectin grafted onto NU-901 (Table S1). After the grafting, NU-901-A is expected to become hydrophobic due to the alkane chains of asolectin; this can be exploited for the bilayer coating with the hydrophobic PGG block of F-127. Figure 2b compares the DLS results for NU-901 and NU-901-A particles. While NU-901 has an original particle size of 145 ± 12 nm (polydispersity index, PDI = 0.764), NU-901-A showed aggregation with sizes of 1.2 ± 0.2 μm (PDI = 0.764). DLS results were consistent even after 1-h sonication in water at room temperature (Figure S10), while SEM showed μm-sized agglomerates of ca. 5 μm (Figure S11), confirming the hydrophobicity of NU-901-A due to the asolectin coating.

In a second step, we suspended NU-901-A in CHCl_3_ and mixed it with an aqueous solution of F-127 (**F**) biosurfactant under rapid stirring, producing **NU-901-A**-**F** (Figure 2c). ^1^H nuclear magnetic resonance (NMR) spectroscopy shows the new peaks of −CH_3_ in the PGG block from F-127 (Figure S12), confirming the existence of F-127 in NU-901-A-F. The interdigitated hydrophobic chains have been proven to be thermodynamically stable.^67^ However, the emulsification step without careful control induced the aggregation of NU-901-A-F particles (898 ± 121 nm; PDI = 0.798) in water (Figure 2d), displaying ca. 3 μm spherical agglomerates under SEM (Figure 2i). Indeed, emulsification-led aggregation is a common phenomenon in the nanoparticle field, including for silica nanoparticles,^68^ quantum dots,^69^ and metal oxides.^70^ During the emulsification process, the NU-901-A were confined in the F-127-stabilized microemulsion droplets, which shrank with the evaporation of CHCl_3_, facilitating the aggregation of NU-901-A-F particles. To avoid emulsification-led aggregation,^71^ we studied the effects of four parameters: 1) the MOF concentration in CHCl_3_, 2) the F-127 concentration in water, 3) the water:CHCl_3_ ratio, and 4) the stirring speed during the emulsification process. Table 1 shows the effect of these four parameters on the particle size. We found that by halving the MOF concentration, as well as increasing F-127 concentration, the water:CHCl_3_ ratio, and stirring speed, it is possible to reduce the hydrodynamic size of coated-NU-901 from 984 to 593, 352, 486, and 320 nm, respectively. Using this approach, we, therefore, successfully obtained optimized NU-901-A-F (Figure 2d) when using 2.5 mg/mL of MOF in CHCl_3_, 2 mg/mL of F-127 in water, 20:1 of water:CHCl_3_ ratio, and 1500 rpm. This NU-901-A-F had a hydrodynamic size of 151 ± 23 nm and PDI of 0.102, demonstrating its high monodispersity. From ICP analysis, we understand that the incorporated bilayer is nontrivial, with 36.9 wt % in NU-901-A-F (Table S1). The crystallinity and morphology of optimized NU-901-A-F NPs also remained consistent with that of bare NU-901 (Figure 2g,j). Interestingly, we observed a significant change in the zeta potential after the coating; Figure 2f shows the evolution of the z-potential, with an apparent decrease in the zeta potential during the two-step coating process. It starts with a z-potential of 32.5 ± 2.9 eV for bare NU-901, suggesting that the dominant ending groups on the surface are Zr_6_ clusters. This value decreases to 6.7 ± 2.2 eV after coating with asolectin, due to neutral/zwitterionic asolectin starting to occupy the available binding sites in the Zr_6_ cluster. Again, this value further decreased to −16.6 ± 3.9 eV after coating with F-127, which is attributed to the attraction of OH^–^ ions by the hydrophilic PEG block of F-127 biosurfactant.^72^

**Table 1:** Effects of Four Parameters on the Hydrodynamic Size of Bilayer-Coated NU-901

MOF concentration in CHCl_3_(mg/mL)	F-127 concentration in water (mg/mL)	H_2_O: CHCl_3_ (mL: mL)	stirring speed (rpm)	hydrodynamic size (nm)
5	1	1	750	984 ± 121
2.5	1	1	750	593 ± 56
5	2	1	750	352 ± 29
5	1	2	750	486 ± 33
5	1	1	1500	320 ± 25

We next evaluated the effect of the bilayer coating on the porosity of the MOFs by measuring the N_2_ uptake at 77 K of the different materials. Figure 2e shows the N_2_ adsorption isotherms. The amount of N_2_ adsorbed at *P*/*P*_0_ = 0.8 (i.e., the total pore volume) by NU-901, NU-901-A, and NU-901-A-F reduced from 737 cm^3^/g to 378 and 245 cm^3^/g, respectively, with BETSI areas^65^ decreasing from 1951 m^2^/g to 959 and 599 m^2^/g, respectively (see Section S5 for more details about the use and results of BETSI fitting). Figure 2h shows the NLDFT PSD. From NU-901 to NU-901-A, although the total pore volume reduces due to some pore blocking, the PSD remains unchanged, suggesting that asolectin is preferentially grafted on the external surface of NU-901. This is likely due to the short grafting time of 4 h. Similarly, NU-901-A-F only shows a slight reduction in the calculated pore size from 26 to 25 Å. Altogether, these results suggest that the bilayer coating mainly occurs on the external surface of NU-901 and does not compromise the internal porosity of the nanoMOFs.

## 4 Colloidal Stability of Coated and Bare MOFs

**Figure 3:**
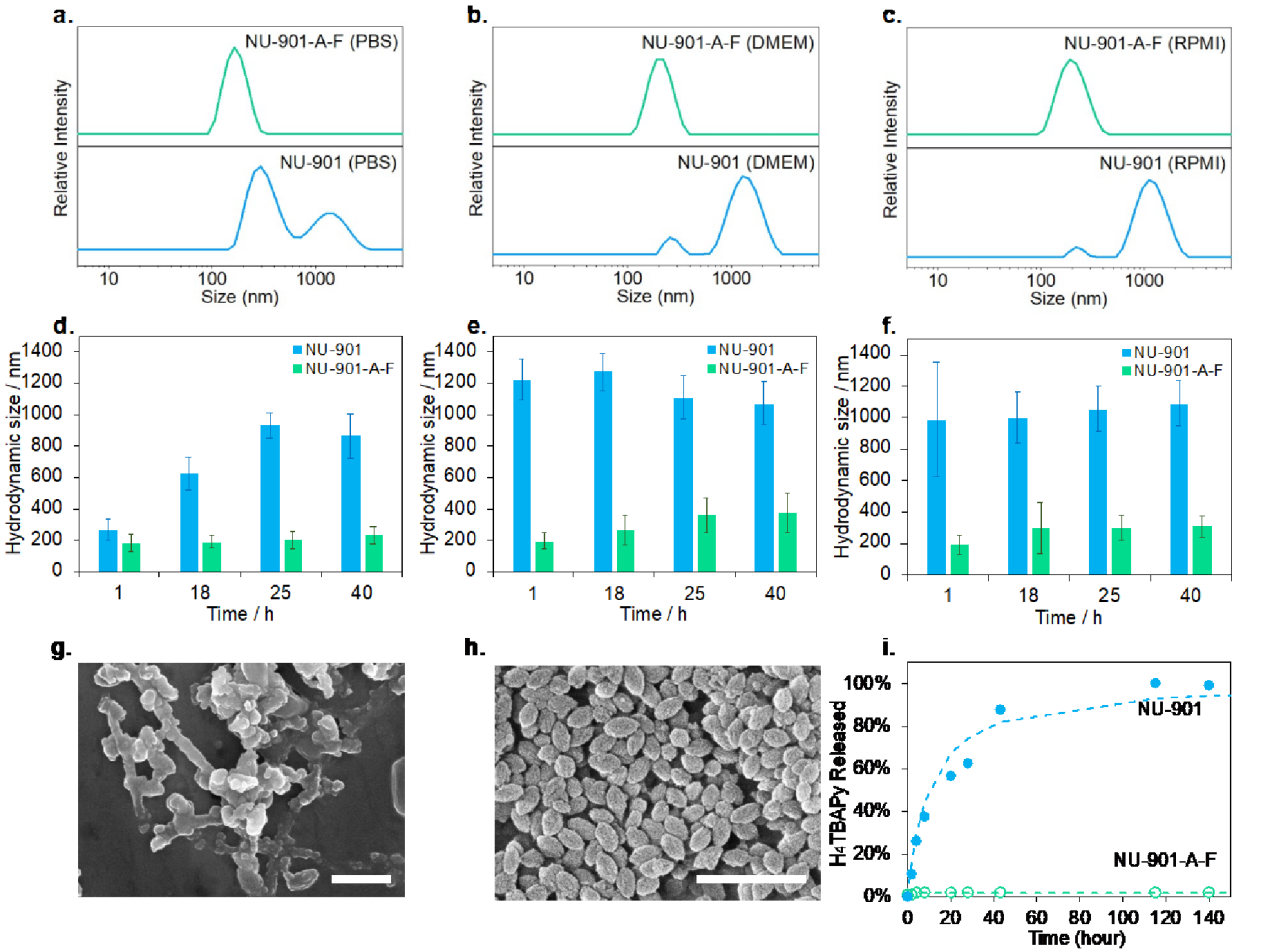
Intensity-average size of the suspension of NU-901 and NU-901-A-F in (a). PBS, (b). DMEM, and (c). RPMI. Change in hydrodynamic size of bare and coated NU-901 in (d). PBS, (e). DMEM, and (f). RPMI at *t* = 1, 18, 25, and 40 h; SEM images of (g). bare NU-901 and (h). coated NU-901 after 48-h dispersion in PBS at 37 °C, both scale bars = 500 nm. (i). Degradation of H_4_TBAPy ligands from coated and uncoated NU-901 in PBS solution for up to 460 h (19 days, error bars are shown in both instances but smaller than symbol size).

After we optimized the bilayer coating conditions, we compared the instant and long-term particle dispersity of bare and coated NU-901 in three biological media: phosphate-buffered saline (PBS, pH = 7.4), Roswell Park Memorial Institute (RPMI) medium, and Dulbecco’s modified eagle medium (DMEM) at 37 °C to mimic the body environment. Indeed, as described above, the aggregation of Zr MOFs in these media is a common issue^44^ that can undermine the biomedical application of MOFs. This is due to the severely impeded biodistribution of micrometer scale particles, decreasing cellular uptake efficiency and increasing toxicity.^59^ We first prefiltered each medium using a 0.2 μm membrane to avoid unnecessary protein or salt colloids affecting DLS measurements; we then dispersed NU-901 and NU-901-A-F in the media, using DLS to check their hydrodynamic size. Figure 3a–c shows the hydrodynamic size of coated and bare NU-901 in PBS, RPMI, and DMEM, respectively. Bare NU-901 aggregates instantaneously, offering sizes of 412 ± 118, 1020 ± 431, and 986 ± 365 nm in the three buffers. In comparison, the coated NU-901-A-F particles avoid aggregation for all three media, showing sizes of 168 ± 39, 199 ± 50, and 191 ± 59 nm, consistent with the size of the pristine NU-901 particles in water (145 ± 12 nm). Expecting typical circulation and internalization times of nanoparticles in the body from 15 min to 4 h,^73,74^Figure 3d–f shows the extended time of the colloidal stability study for up to 40 h. Bare NU-901 aggregated even more severely, whereas coated NU-901 remained well dispersed: within 40 h, the size of NU-901 dispersed in PBS sharply increased from 266 ± 69 nm (1 h) to 932 ± 82.25 nm (25 h). When using DMEM and RPMI, the aggregation of uncoated NU-901 particles was even more pronounced, with an average hydrodynamic size greater than 1 μm in the 40-h time frame (Figure 3e,f), whereas, in water, where no salts are present, the particles remained stable (Figure S13a). In contrast, NU-901-A-F remained colloidally stable and dispersed in all solvents—especially in PBS—keeping a particle size of ca. 180 nm. Although NU-901-A-F slightly increased its size in DMEM and RPMI toward the end of stability studies, their size remained valid for cellular uptake.^22^ Once more, the hydrophilic PEG chains of F127 in the aqueous solution can generate active steric repulsion among MOF particles to improve their colloidal stability. All in all, the results demonstrate our hypothesis that the additional bilayer coatings are able to improve the long-term dispersity of nanoMOF particles.

Colloidal stability is not the only issue when phosphate ions are present. Their attack on the metal centers^26^ leads to a structure collapse, provoking the burst release of drugs in less than 48 h. We evaluated, therefore, the ability of the bilayer to avoid coordination of phosphate salts to NU-901 and the release of the organic ligand. We first incubated NU-901 and NU-901-A-F separately in PBS (pH = 7.4) at 37 °C for 48 h; we then compared their morphology and crystallinity using SEM and PXRD. Whereas the morphology of NU-901 changed drastically (Figure 3g), with the PXRD showing a largely amorphous material (Figure S13b), NU-901-A-F preserved both the oval-shape morphology (Figure 3h) and crystallinity (Figure S13b). Figure 3h shows the release of the H_4_TBAPy ligand from bare and coated NU-901 over up to 150 h; Figure S14 shows the calibration curve. When exposed to PBS, bare NU-901 rapidly releases more than 80% of its ligands in ca. 48 h. In contrast, NU-901-A-F did not release any detectable ligand for at least 140 h. Combined with the achievement of successful colloidal stability, these results show the effectiveness of the bilayer coating in offering protection against phosphate attack. First, asolectin provides a shielding effect on the Zr-metal nodes through Zr–O–P coordination. Second, the hydrophobic region of the alkane chains from asolectin and the PGG blocks from F-127 offer an additional shield around the MOF nanoparticles, impeding the diffusion of phosphate ions toward the metals.

## 5 Long-Term Storage and Drug Release

**Figure 4:**
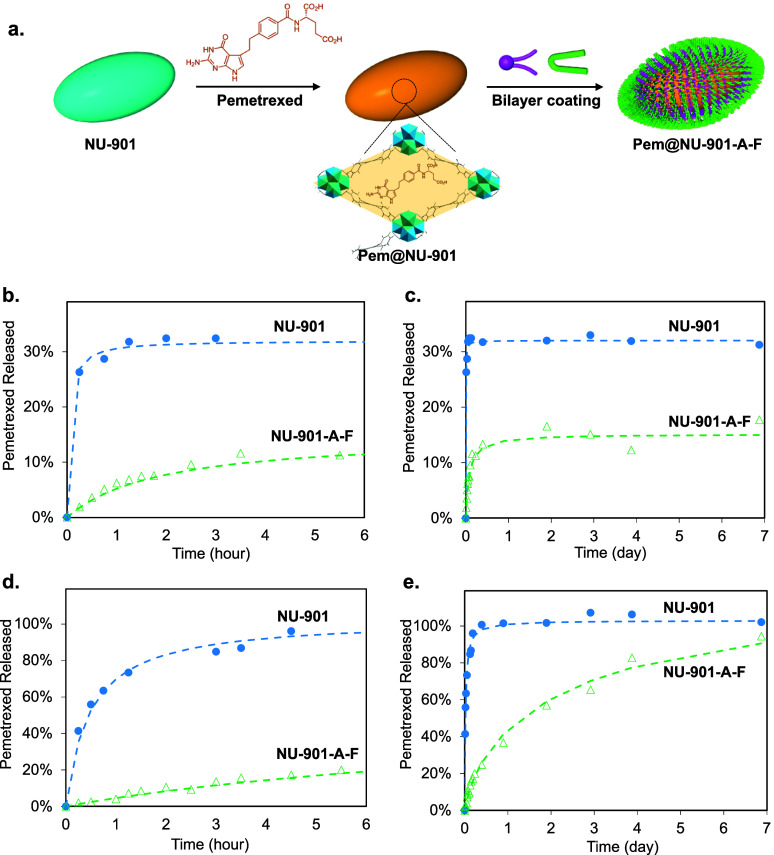
Pemetrexed loading and release in NU-901. (a). Schematic illustration of loading pemetrexed into NU-901 to obtain Pem@NU-901, followed by bilayer coating to obtain Pem@NU-901-A-F. Release of pemetrexed from NU-901 and NU-901-A-F in water for the (b) first 6 h and (c) 7 days at 37 °C. Release of pemetrexed from bare and coated NU-901 in PBS for the (d) first 6 h and (e) 7 days at 37 °C.

Long-term stability and optimal storage of drug-loaded MOFs in water are crucial in healthcare applications since dried MOFs can lead to irreversible aggregation,^38^ elevating the risk of embolia during administration in vivo.^38^ Here, we wanted to evaluate the capability of the A-F bilayer coating to prevent the leakage of drugs from the MOF, avoiding the burst effect and allowing their long-term storage. We first loaded pemetrexed (**pem**)^50,75^ into NU-901 to get **pem@NU-901**. Then, we applied the bilayer coating to obtain **pem@NU-901-A-F** (Figure 4a). The crystallinity, particle size, and morphology of pem@NU-901-A-F were maintained (Figures S17,S18), while the drug loadings decreased from 25.1 ± 0.7 to 15.0 ± 0.2 wt % for pem@NU-901 and pem@NU-901-A-F (Figure S15). BETSI analysis indicates a significant reduction of the BET area from 1951 to 1254 m^2^/g after loading NU-901 with pemetrexed (see Section S5 for more details). The NLDFT PSD also shows a sharp decrease in the pore volume of NU-901 after being loaded with pemetrexed, together with the calculated average pore size reducing from 26 to 22 Å. This indicates that a considerable portion of pemetrexed molecules occupies the internal channels of NU-901 (Figure S16). Applying the following bilayer coating (pem@NU-901-A-F) leads to a further decrease of N_2_ uptake and the BETSI area to 676 m^2^/g as a result of the pore-blocking effect. Interestingly, this BETSI area is higher than that of NU-901-A-F (599 m^2^/g); this can be understood as small experimental errors on the N_2_ adsorption experiment or in the coating process that partially blocks access to the porosity when working at 77 K. All in all, although the drug loading decreased after the coating, the results confirm the compatibility of the coating with the cargo loading.

After loading pemetrexed, we evaluated its release from bare and coated NU-901-A-F in water and PBS over 7 days at room temperature (see Section S4 for full details). Figure 4b–e show the release curves of NU-901 and NU-901-A-F in water and PBS, respectively. Interestingly, in water, NU-901 releases 32% of the pemetrexed in the first 2 h, followed by a plateau for at least 7 days. In PBS, 98% of pemetrexed is released in the first 6 h. The burst release can be related to the desorption of pemetrexed from NU-901 in water, as well as by the MOF degradation through the phosphate salts in PBS. In the case of water, the coordination bonds between the COOH group of pemetrexed and the Zr_6_ cluster of NU-901 could be the reason for limiting further release beyond 32%.^76^ In contrast, NU-901-A-F only released 8% of pemetrexed in the first 2 h in water and 14% after 7 days. This behavior is essential for the long-term storage of MOF DDS, with, in our case, the bilayer inhibiting the diffusion of adsorbed pemetrexed out of the framework. In PBS, NU-901-A-F released only 20% of pemetrexed in the first 6 h (Figure 4d), which is about 5 times lower than NU-901. Over 1 week, NU-901-A-F released pemetrexed in a much slower manner throughout the entire period, reaching ca. 95% just on day 7 (Figure 4e). We consider that the bilayer coating effectively impedes the desorption of pemetrexed and the diffusion of phosphate salts toward the inner NU-901 core, protecting its structure in PBS at body temperature. These results confirm that with the assistance of the bilayer, the MOF can avoid the burst effect. We have shown in the past slow-release MOF systems developed through mechanical and temperature-based amorphization methods that lead to the loss of crystallinity of MOFs and the entrapment of drugs in the porosity.^24,77^ However, these methods might represent a risk for delicate cargoes such as macromolecules at elevated temperatures.^78,79^ The proposed bilayer coating shown here, on the other hand, is easy to implement and cargo agnostic, being compatible with small drugs and macromolecules. Indeed, the MOF grafting is relatively benign, avoiding high mechanical stress and high temperatures and not harming RNA or peptides during the process.

## 6 Uptake of NU-901 and NU-901-A-F by Cancer Cells

The endocytosis mechanism of MOFs and the understanding of their final fate in vitro and in vivo are of great importance in healthcare applications and require detailed attention.^80^ For example, it is essential to understand if the delivery system can be internalized through the cellular membrane, as this will impact its biodistribution in vivo. Here, we examined the interaction between nanoparticles and two human thoracic cancer models: A549 is a nonsmall cell lung cancer cell line, and 3T is a low-passage epithelioid pleura mesothelioma cell line.^47^ We exploited the fluorescence properties of the pyrene linkers of NU-901 MOFs for their detection via flow cytometry and microscopy. Neither pemetrexed loading nor bilayer coating affected the fluorescence of NU-901-based MOFs (Figure S22).

**Figure 5:**
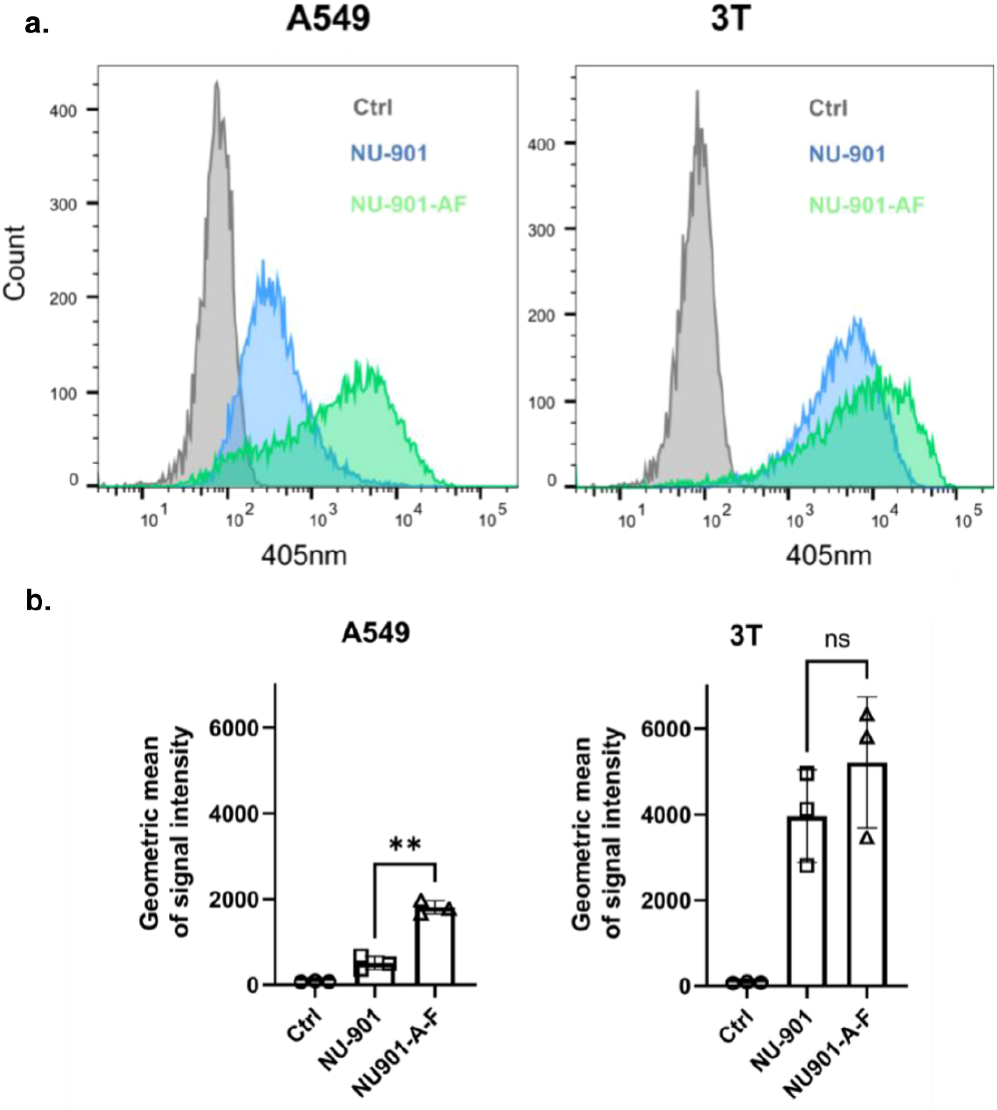
Cellular association with NU-901 and NU-901_A-F. (a). Representative histograms of fluorescent (405 nm) cell populations, A549 and 3T, exposed to NU-901 vs NU-901-A-F nanoparticles for 16 h, acquired via flow cytometry, *n* = 3. (b). The geometric mean of fluorescence intensity, *n* = 3. Paired student *t* test: **p = 0.0027, ns = 0.549.

To study the internalization of MOFs by cancer cells, A549 and 3T cells were exposed to NU-901 or NU-901-A-F for 16 h and then washed before flow cytometric analysis. Figure 5 shows the cellular association with NU-901 and NU-901-A-F obtained by flow cytometry. Increased fluorescence signals in the treated samples confirmed the strong association of MOFs with both cell lines (Figure 5a). In A549 cells, the uptake of coated NU-901-A-F (signal intensity: 1750 au) was significantly higher than that of its uncoated counterpart NU-901 (signal intensity: 500 au) at the time point tested (Figure 5b). We have seen previously that the size and surface chemistry of the delivery system can influence the cellular uptake.^22,26^ Since the bilayer coating improves the colloidal stability and the monodispersity of the MOF nanoparticles, it enhances their affinity for cancer cells. Differences in affinity between coated and uncoated MOF for A549 and 3T cell lines could be related to cell size (3T3 being larger) and differences in cell surface proteomes. Indeed, a larger cell surface area might facilitate their interaction with the nanoparticles (Figure 5), showing a much higher signal intensity in 3T cells exposed to either uncoated or coated MOFs. Additionally, there is a small tendency for increased affinity for the coated MOF in 3T cells; this marginal increase could be explained by the already high affinity displayed by the uncoated NU-901 in these cells.

**Figure 6:**
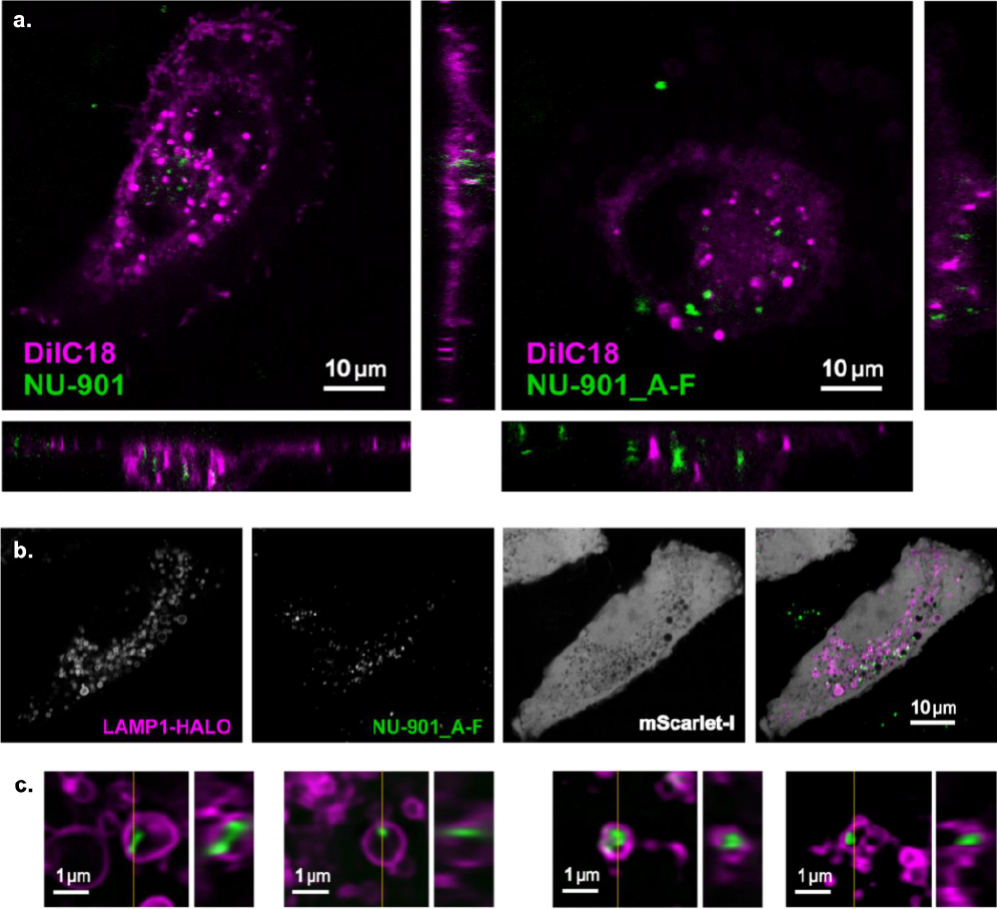
Subcellular localization of NU-901-based MOFs in cancer cells. (a). Live-cell confocal micrographs (LSM880 Airyscan microscope) of 3T cells exposed to NU-901 or NU-901-A-F (green) for 16 h and stained with DilC18 (magenta), including three orthogonal views. (b). Live cell confocal imaging of A549 cells transiently transfected with mScarlet-I (gray) and Lamp1-HaloTag (magenta). Cells were exposed to NU-901 (not shown) or NU-901-A-F (green) for 16 h and labeled with Halo ligand JF646 just before acquisition. Merge images show MOFs in close proximity with LAMP1-positive vesicles (lysosomes). Images were processed in ImageJ (Fiji) using a Gaussian blur with standard deviation sigma of 0.75. (c) Live-cell lattice SIM images (acquired on a Zeiss Elyra7 microscope) of A549 and 3T cells expressing LAMP1-HaloTag (magenta) and exposed to NU-901-A-F MOFs (green) for 16 h (2 examples each). Cells were labeled with JF646 HaloTag ligand.^81^ Images were reconstructed by using the Zeiss SIM^2^ algorithm. The yellow line represents the Z plane for each orthogonal projection on the right. Images show MOFs enclosed in lysosomes.

Figure 6 shows the live-cell confocal microscopy performed to characterize the association between cells and nanoparticles at 16 h. Similar results were obtained at 24 h (not shown). The fluorescent lipophilic dye DilC18 was used to identify the cell boundary (magenta), and NU-901 MOFs (green) were visualized by excitation at 405 nm (Figure 6a). The orthogonal projections confirmed that NU-901 and NU-901-A-F entered the cells. From the orthogonal projections, both NU-901 and NU-901-A-F were observed inside the cells rather than at the surface. We then set out to determine the subcellular localization of the internalized MOFs. Using transiently transfected fluorescent markers (cytosolic mScarlet-I and LAMP1-HaloTag to visualize lysosomes), we observed MOFs (green) near LAMP1-positive vesicles (magenta) (Figure 6b). However, the optical resolution limited our ability to assess the nature of this interaction. We, therefore, moved to structured illumination microscopy (SIM) to achieve subdiffraction limit resolution. Figure 6c shows live-cell lattice SIM images of A549 and 3T cells exposed to NU-901-A-F for 16 h; Supplementary Movies 1 and 2 show the SIM videos. Here, we observed coated NU-901 inside cells within lysosomes marked by LAMP1-HaloTag. These data demonstrate that both coated and uncoated MOFs are localized within lysosomes in two models of thoracic malignancy. To our knowledge, this represents the first example of live imaging to capture the internalization of the MOF DDS by primary mesothelioma cells.

## 7 Cytotoxicity of Pemetrexed-Loaded NU-901 Vs NU-901-A-F

**Figure 7:**
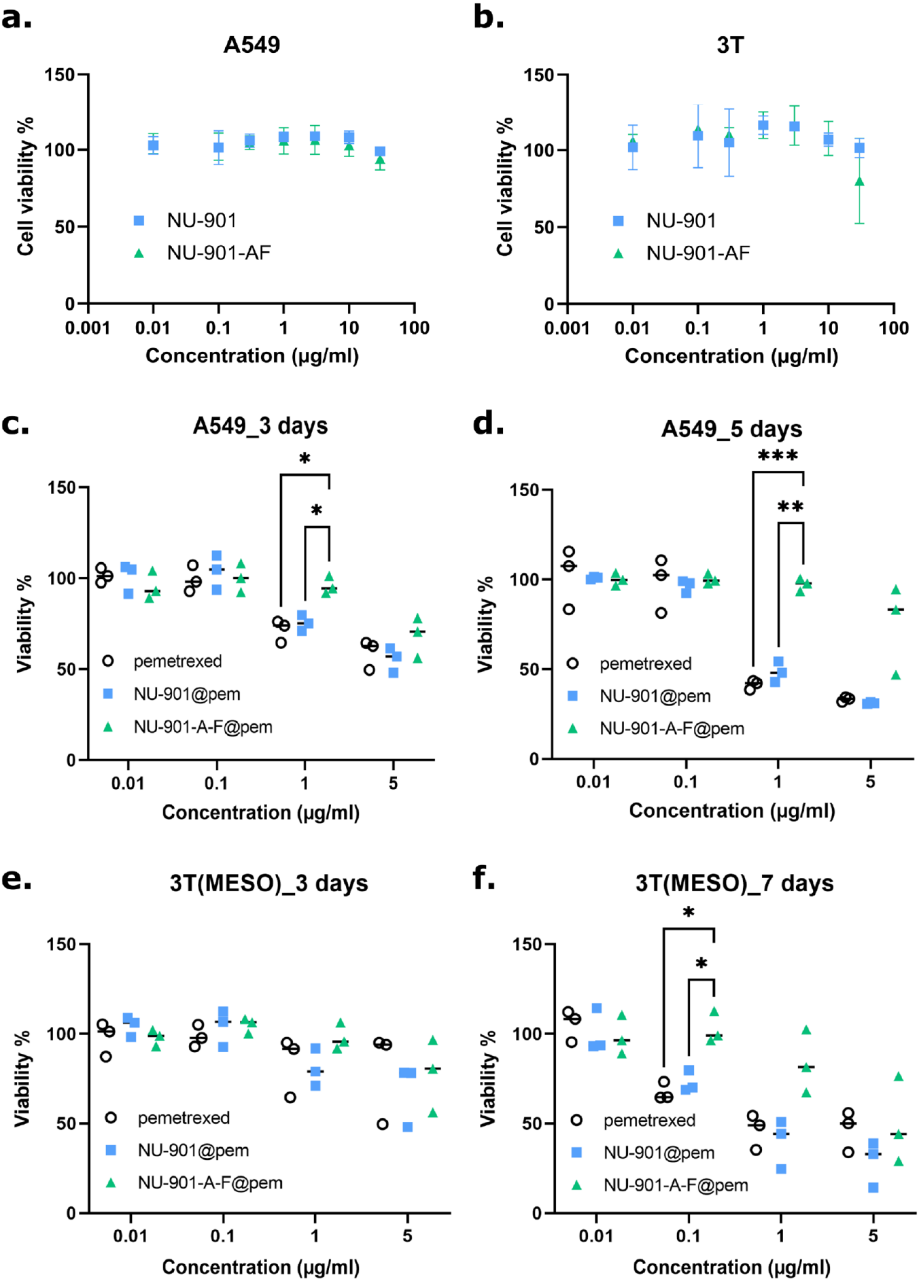
Cytotoxicity of NU-901-based nanoparticles. CellTiterGlo assay in (a). A549 and (b). 3T cells exposed to unloaded NU-901 vs NU-901-A-F MOFs for 72 h, which were tolerated by the cells up to 10 μg/mL in these conditions. Data are means ± SD. Bottom panels show exposure to pemetrexed-loaded nanoparticles vs pemetrexed alone (equivalent doses) for (c). 3 days or (d). 5 days in A549 cells. Similar experiments were conducted in 3T mesothelioma cells for (e). 3 days or (f). 7 days. *n* = 3 biological replicates, data medians are shown. 2-way ANOVA with Tukey’s multiple comparison test (***p ≤ 0.001, **p ≤ 0.01, *p ≤ 0.05).

Cell viability was assessed after 72 h of exposure with both coated and uncoated nanoparticles (Promega, CellTiterGlo) on A549 and 3T cells. When not loaded with the drug, both MOFs were well tolerated up to 10 μg/mL (Figures 7a,b). As expected, both cell lines were susceptible to killing by the pemetrexed solution, with an IC_50_ of 0.15 and 0.45 μg/mL for A549 and 3T cells, respectively, after 3 days of exposure (Figure S21). Following this, we prepared free pemetrexed, pem@NU-901, and pem@NU-901-A-F at the same pemetrexed concentration as a stock solution prior to each experiment, which was then diluted to the desired concentrations for cell culture. The pemetrexed-loaded NU-901 (pem@NU-901) MOFs were equally toxic to an equivalent dose of free pemetrexed (i.e., the same amount of pemetrexed was delivered to the cell culture dish) at the time points tested (Figure 7c–f). Consistent with the slower drug release profile (Figure 4), pem@NU-901-A-F showed a delayed cytotoxic effect in both cell models (Figure 7d–f), suggesting the avoidance of the burst effect in the coated-MOF system.

## 8 Generality and Versatility of the Bilayer Coating Method

**Figure 8:**
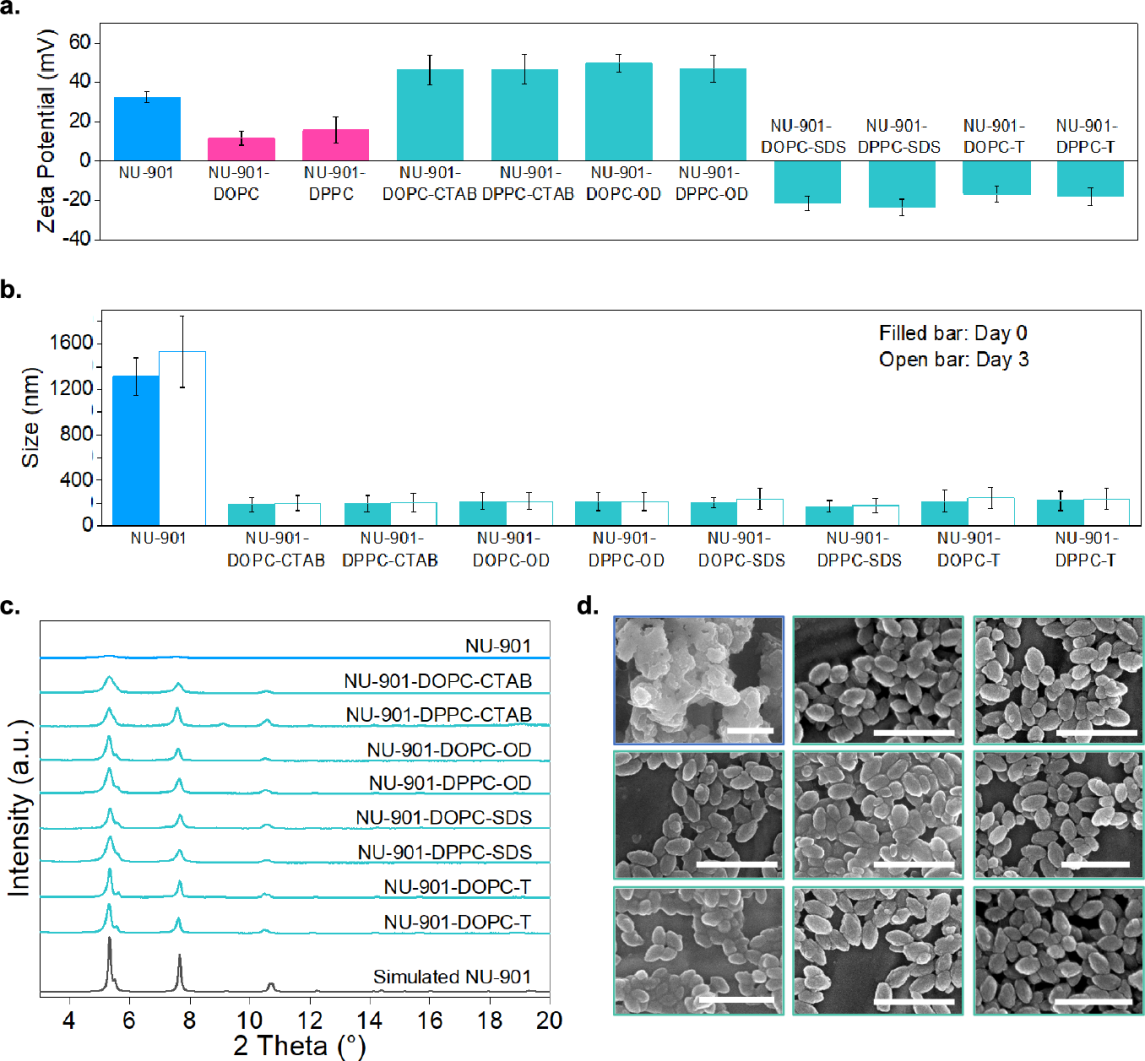
(a). Zeta potential of NU-901, lipid-coated NU-901 after SALI, and bilayer-coated NU-901 after emulsification. (b). Hydrodynamic sizes of uncoated and coated NU-901 on days 0 and day 3. (c). PXRD patterns of simulated NU-901, uncoated, and coated NU-901 NPs after 2-days incubation in PBS at 37 °C. (d) SEM images of bare (blue frame) and coated NU-901s (green edges) after 2 days of incubation in PBS at 37 °C, from left to right/top to bottom: NU-901, NU-901-DOPC-CTAB, NU-901-DPPC-CTAB, NU-901-DOPC-OD, NU-901-DPPC-OD, NU-901-DOPC-SDS, NU-901-DPPC-SDS, NU-901-DOPC-T, and NU-901-DPPC-T. Scale bar = 500 nm.

Once the slow drug release was verified, we probed the generality of the bilayer coating approach by extending the method to other lipids and surfactants. We first used SALI to coat NU-901 with two lipids separately, 1,2-dioleoyl-*sn*-glycero-3-phosphocholine (DOPC) and 1,2-dipalmitoyl-*sn*-glycero-3-phosphocholine (DPPC). This yielded NU-901-DOPC and NU-901-DPPC. We subsequently applied the emulsification described above to add four different surfactants that were used in functionalizing and stabilizing drug-delivery nanoparticles,^37,46,82,83^ cetrimonium bromide (**CTAB**, cationic), octenidine dihydrochloride (**OD**, cationic), sodium dodecyl sulfate (**SDS**, anionic), and Tween 80 (**T**, nonionic). This produced 8 different materials, NU-901-DOPC-CTAB, NU-901-DPPC-CTAB, NU-901-DOPC-OD, NU-901-DPPC-OD, NU-901-DOPC-SDS, NU-901-DPPC-SDS, NU-901-DOPC-T, and NU-901-DPPC-T. We measured the zeta potentials of the eight sets of coated particles to confirm their changes in surface chemistry. After coating NU-901 with DOPC and DPPC, the zeta potential of NU-901 reduced from +32.5 ± 2.8 mV to +11.5 ± 2.9 eV and +15.6 ± 5.4 eV, respectively (Figure 8a). Subsequently, both cationic CTAB and OD increased the zeta potential to ca. + 40 mV, while anionic SDS reduced the zeta potential to around −22 eV. NU-901-DOPC-T and NU-901-DPPC-T showed less negative zeta potentials of −16.8 ± 4.3 and −18.1 ± 4.4 eV, respectively.

After confirming the changes in the external surface chemistry, we evaluated the protection afforded by each bilayer coating against NU-901 degradation. We compared the crystallinity and morphology of the bare and the 8-coated NU-901-based samples after dispersing them in PBS for 2 days at 37 °C. Figure 8c shows the PXRD patterns of the different materials; Figure 8d shows representative SEM images of the materials. While the 8-coated NU-901 retained its crystallinity, bare NU-901 samples lost it due to the phosphate attack. Similarly, the coated materials kept the original morphology, in contrast to the serious corrosion of uncoated NU-901. We then compared the instant and long-term particle dispersity of bare and coated NU-901 in PBS at 37 °C for 3 days. Figure 8b shows that bare NU-901 aggregated instantaneously, with a hydrodynamic size of 1311 ± 164 nm in PBS, which increased to 1532 ± 312 nm after 3 days, whereas all the 8 coated NU-901 samples remained well dispersed at around 200 nm throughout the 3 days. Altogether, by extending the bilayer coating method using different lipids and surfactants, we demonstrated the versatility and generality of this strategy to enhance the stability of our Zr-MOF.

## 9 Outlook

We have successfully developed a two-step postsynthetic coating method to increase the colloidal stability of metal–organic frameworks in biological media. Using NU-901 as a model nanoparticle, we first coated the MOF with an economic lipid, asolectin, as a shield from phosphate attack, followed by grafting the F-127 biosurfactant to enhance the dispersity in cell media. By carefully controlling the concentration of the modulator 4ABA, we could tune the particle size and topology simultaneously. We then successfully produced a dispersed biosurfactant and asolectin-coated NU-901 by controlling four parameters: NU-901 concentration, F-127 concentration, H_2_O:CHCl_3_ ratio, and stirring speed. The bilayer coating improved the nanoparticle stability in PBS and enhanced dispersity in cellular media, addressing the early degradation and aggregation issues that hindered Zr-MOF biocompatibility. In addition, when MOFs were loaded with the chemotherapeutic drug pemetrexed, the bilayer coating accounted for a slower drug release, which was reflected in a delayed cytotoxic effect in cancer cell models. This altered pharmacokinetics could enable less frequent drug-dosing regimens. We investigated the cellular uptake of coated vs uncoated MOFs using flow cytometry and found that in A549 cells, the bilayer coating increased it significantly. The shift in surface chemistry for the coated MOF, from positively charged to negatively charged, may be responsible for enhancing the affinity for the cell membrane. In the mesothelioma model, MOF uptake was high for both MOFs after 16 h of treatment, and the coating only marginally increased it. Furthermore, we used live imaging (confocal and super-resolution) to determine the subcellular localization of MOFs in the two cancer cell models. We saw that both MOFs were internalized and localized inside lysosomes, regardless of coating. While unloaded MOFs were well tolerated by cells, we demonstrated that pem@NU-901 and pem@NU-901-AF MOFs were competent in delivering the drug to cells by measuring their cytotoxic effects compared to equivalent doses of the drug alone. Altogether, our bilayer method provides a convenient and economical way to modify drug-loaded MOF nanocarriers, which are promising for biomedical applications.

## 10 Experimental/Methods

### 10.1 Powder X-Ray Diffraction (PXRD)

Powder X-ray diffraction (PXRD) data were collected on a Bruker D8 DAVINCI diffractometer at 298 K by using Cu Kα radiation. The calculated PXRD patterns were produced using the Mercury program and single crystal reflection data.

### 10.2 Gas Uptake

N_2_ sorption isotherm measurements were performed on a Micromeritics 3Flex analyzer at 77 K. Samples were degassed under vacuum at 120 °C for 20 h using the internal turbopump. The surface areas were calculated using BETSI.

### 10.3 Dynamic Light Scattering

Measurements were recorded on a Zetasizer Nano ZS (Malvern Instrument Ltd., U.K.) equipped with a He–Ne laser operating at 633 nm and 25 °C.

### 10.4 UV–Vis Fluorescence Spectroscopy

UV–vis and fluorescence spectra were recorded by using a Tecan Spark Multimode Microplate Reader.

### 10.5 Scanning Electron Microscopy (SEM)

The samples for the SEM test were coated with Pt for 40 s and imagined using an FEI Nova Nano SEM 450.

### 10.6 Transmission Electron Microscopy (TEM)

The samples for the TEM test were prepared by dispersing the samples in ethanol using ultrasonication. After that, a small number of suspensions were drop-cast on a copper grid with a carbon support film. TEM micrographs were collected on a Tecnai F20 instrument with an acceleration voltage of 200 kV.

### 10.7 Inductively Coupled Plasma–Optical Emission Spectroscopy (ICP-OES)

ICP-OES was performed using a Perkin Elemer ICP-OES Optima 2100DV. Samples were dispersed in 2 mL of nitric acid and 6 mL of hydrochloric acid (*CAUTION!*) and left to stand at room temperature in the fume cupboard for at least 1 h until all reactions have ceased. After that, samples were heated at 90 °C for 10 h to fully digest the sample. The mixture was diluted 750 times before the measurement.

### 10.8 Nuclear Magnetic Resonance (NMR) Spectroscopy

^1^H NMR spectroscopy was performed on a 500 MHz DCH Cryoprobe NMR instrument at 25 °C.

### 10.9 Thermogravimetric Analysis (TGA)

TGA measurements were carried out by using a TA Instruments Q500 Thermogravimetric Analyzer. Measurements were collected from room temperature to 800 °C with a heating rate of 20 °C/min under nitrogen.

### 10.10 Cell Culture

A549, an adenocarcinomic alveolar basal epithelial cell line (nonsmall cell lung cancer), was obtained from ATCC and was maintained in Dulbecco’s modified Eagle’s medium (DMEM) (Sigma) supplemented with 10% fetal bovine serum (FBS) (Lonza) and penicillin (100 U/mL) and streptomycin (100 μg/mL). 3T, a low-passage primary cell line derived from epithelioid pleura mesothelioma, was obtained from MesobanK (Rintoul et al. 2016, PMID: 26467803) and was maintained in RPMI-1640 media (Sigma) supplemented with 10% FBS, l-glutamine (2 mM), penicillin (100 U/mL), streptomycin (100 μg/mL), hEGF (20 ng/mL), hydrocortisone (1 μg/mL), and heparin (2 μg/mL) as described in Chernova et al.^47^ (PMID: 26891694). All cells were cultured in a 5% CO_2_ humidified atmosphere at 37 °C.

### 10.11 Cell Viability Assay

3T and A549 cells were seeded at the concentration of 5 × 10^4^ cells per well in triplicates in optical bottom 96-well plates. Cells were treated with pemetrexed (Sigma), unloaded MOFs, or MOFs loaded with pemetrexed as described above. Cells were incubated for the indicated time points at 37 °C in 5% CO_2_ and cell viability was determined using a bioluminescence-based commercially available kit, CellTiterGlo (Promega), following the manufacturer’s instructions. The luminescence signal was measured using a Tecan Spark Multimode Microplate Reader. Data are presented as percentages of surviving cells compared to controls.

### 10.12 Flow Cytometry

3T and A549 cells were seeded at the density of 5 × 10^5^ cells per well on 6-well plates and allowed to attach for 6 h before treatment. Cells were exposed to 6 μg/mL of NU-901 or NU901-AF for 16 h. Before analysis, excess MOFs were washed away from the cells with PBS. Cells were then detached from plates using Trypsin/EDTA solution (Gibco) and collected by centrifugation at 400 g for 4 min. After 3 washes with PBS, cells were resuspended in 500 μL 2% FBS in PBS for detection of fluorescence at 405–50 nm via flow cytometry. Data were acquired using a BD LSRFortessaTM Cell Analyzer (BD Biosciences), and the population of interest was gated according to its FSC/SSC criteria. Analysis was conducted with FlowJoTM software (BD Biosciences).

### 10.13 In vitro Microscopy

3T primary mesothelioma cells and A549 cells were plated at a density of 1000 cells/cm^2^ on 30 mm diameter glass coverslips. Four h post seeding, cells were transfected using 1 μg of plasmid DNA and a Lipofectamine 2000 (Invitrogen) in 1:3 ratio. Mammalian expression plasmids encoding mScarlet-I and LAMP1-HaloTag were a kind gift from Jonathon Nixon-Abell (CIMR, Cambridge UK). Two hours post-transfection, a stock suspension of MOFs was agitated by sonicating water bath, diluted to 10 μg/mL in culture medium, and added to cells. After 16 h of MOF incubation, cells were washed 3 times with 3 mL of PBS. Untransfected cells were labeled for 15 min with 2.5 μg/mL DilC18 (1,1′-Dioctadecyl-3,3,3′,3′-Tetramethylindocarbocyanine Perchlorate, Thermofischer, Catalog number: D3911) followed by 3 PBS washes and addition of fresh culture medium prior to imaging. Transfected cells were labeled with JF646 HaloTag ligand [PMID: 25599551] followed by 3 PBS washes and the addition of fresh culture medium prior to imaging. Confocal live-cell imaging was performed on a Zeiss LSM780 confocal microscope with GaAsP detectors using a 63× 1.4 NA oil immersion lens. MOFs, DilC18, iScarlet, and JF646 were excited at 405, 561, 561, and 633 nm wavelengths, and emissions were collected at 410–556, 578–696, 579–650, and 652–755 nm, respectively. Super-resolution, live-cell Lattice SIM images were acquired on a Zeiss Elyra7 microscope using a ×63 1.4 NA oil immersion objective. Images were reconstructed using the Zeiss SIM^2^ algorithm in three dimensions using the “standard–live” settings with the sectioning set at 92. MOFs and HaloTag-JF646 were excited at 488 and 642 nm, respectively, and emitted light was captured simultaneously using an OptoSplit beam splitter and two pco.edge sCMOS cameras filtered with band-pass 420–480 nm + band-pass 495–550 nm (MOFs) and band-pass 570–620 + long-pass 655 nm (HaloTag-JF646).

## 11 Materials and Synthesis

### 11.1 Preparation of 4,4′,4″,4′″-(Pyrene-1,3,6,8-tetrayl)tetrabenzoate (H_4_TBAPy)

H_4_TBAPy was prepared according to the protocol by Wang et al.^2^ Tetrabromopyrene (0.5 g, 0.97 mmol), 4-ethoxycarbonylphenylboronic acid (8.25 g, 42.5 mmol), potassium phosphate tribasic (16.5 g, 77.7 mmol), and tetrakis(triphnylphosphine)-palladium(0) (0.75 g, 0.65 mmol) were added to a 2-necked round-bottom flask and purged with N_2_ for 1 h. Degassed dioxane (270 mL) was injected, and the solution heated to 90 °C for 72 h before 300 mL of water was added and let the reaction cool down at room temperature for 1 h. The yellow solid was collected under Büchner filtration and washed with 100 mL of water (24 mL) and 200 mL of acetone. The product was dissolved in boiling chloroform (300 mL) and filtered, followed by reducing the volume of the collected chloroform solution containing the product with rotor evaporator to 100 mL. The product was precipitated with methanol (300 mL) and isolated under Buchner filtration before being dried at 70 °C for 12 h in a vacuum oven, giving 4,4′,4″,4′″-(pyrene-1,3,6,8-tetrayl)tetrabenzoate, a yellow solid.

4,4′,4″,4′″-(Pyrene-1,3,6,8-tetrayl)tetrabenzoate (4.5 g) was then suspended in dioxane (500 mL), and 400 mL of aqueous potassium hydroxide solution (KOH, 7.1 g) was injected. The mixture was stirred vigorously and refluxed for 20 h, producing a clear solution. Let the solution cool to room temperature and slowly add concentrated HCl (37%, 12 M) until the solution has a pH = 1 in an ice bath. A yellow precipitate was observed after acidification, and the reaction was stirred for an additional 1 h. The yellow precipitate was collected under Büchner filtration, dried, suspended in 200 mL of water, and sonicated for 1 h to ensure all the salt impurities generated in the neutralization were fully dissolved. The product was filtered and washed with 100 mL of water followed by dissolving it in 100 mL of DMF at 120 °C and filtering while it is hot (CAUTION, please wear heat-insulating gloves). The solution was cooled down to room temperature, and 300 mL of dichloromethane were added while stirring (DCM) to obtain yellow precipitate. The yellow solid was collected under filtration and washed with 100 mL of DCM and dried under vacuum at 120 °C for 36 h, yielding 3.7 g of product (bright yellow solid). ^1^H NMR (500 MHz, DMSO-*d*_6_) δ 13.12 (s, 4H), 8.22 (s, 4H), 8.18 (d, J = 8.0 Hz, 8H), 8.10 (s, 2H), 7.88 (d, J = 8.0 Hz, 8H).

### 11.2 Sterile Synthesis of MOFs

To ensure the sterility of MOFs for cellular studies, all solution of reactants was filtered with 0.2 μm PTFE filters, and all apparatus such as vials and stir bars were autoclaved at 120 °C. All the centrifuge tubes were sterile as received during the washing steps, and 70% ethanol and sterile water were both applied in the washing step.

### 11.3 NU-901 Synthesis (∼150 Nm)

ZrOCl_2_·8H_2_O (484 mg, 2.7 mmol), 4-aminobenzoic acid (1050 mg, 7.7 mmol), and 800 μL of TFA were dissolved in DMF (40 mL) to obtain Solution 1. The 4ABA can be replaced by either the same mole of BA or B4CA. H_4_TBAPy (100 mg, 0.146 mmol) was then dissolved in DMF (40 mL) in a separate vial to obtain Solution 2. Solution 1 was mixed with Solution 2 in a 100 mL threaded vial. The resultant mixture was incubated in an oil bath at 140 °C and 700 rpm (rpm) for 50 min. The resulting material was isolated by centrifugation and washed three times with DMF and three times with ethanol. The final product was redispersed in 70% ethanol for in vitro studies.

### 11.4 NU-1000 Synthesis (∼2 μm)

ZrOCl_2_·8H_2_O (484 mg, 1.5 mmol), 4-aminobenzoic acid (5 g, 36.5 mmol), and 800 μL of TFA were dissolved in DMF (40 mL) to obtain Solution 1. The 4ABA can be replaced by either the same mole of BA or B4CA. H_4_TBAPy (100 mg, 0.146 mmol) was then dissolved in DMF (40 mL) in a separate vial to obtain Solution 2. Solution 1 was mixed with Solution 2 in a 100 mL threaded vial. The resultant mixture was incubated in an oil bath at 140 °C and 700 rpm (rpm) for 50 min. The resulting material was isolated by centrifugation and washed three times with DMF and three times with acetone. The final product was redispersed in ethanol for further.

### 11.5 The Procedure for Coating Asolectin to NU-901 (NU-901-A)

Asolectin solution (1 mL, 25 mg/mL in CHCl_3_) was added into the NU-901 suspension (10 mL, 2 mg/mL in CHCl_3_). After stirring (800 rpm) at room temperature for 4 h, the reaction mixture was washed thrice with CHCl_3_ under centrifugation at 15 000 rpm to remove unreacted reagents. The resultant NU-901-A should disperse well in chloroform but aggregate to visible flakes in water due to its strong hydrophobicity. The methods for synthesis of **NU-901-DOPC** and **NU-901-DPPC** were the same as that of NU-901-A.

### 11.6 The Procedure for Coating F-127 to NU-901-A (NU-901-A-F)

NU-901-A suspension (1 mL, 2.5 mg/mL) in CHCl_3_ were mixed with the solution of F-127 (20 mL, 2 mg/mL) in water. The system was then emulsified by ultrasonic treatment for 5 min, followed by rapid stirring at 1500 rpm for 10 min in a capped vial. The cap was then removed to allow the removal of CHCl_3_ to obtain NU-901-A-F at room temperature in a fumehood over 12 h. The final product was washed trice with 70% ethanol and dispersed in sterile water. The methods for synthesis of **NU-901-DOPC-CTAB**, **NU-901-DPPC-CTAB**, **NU-901-DOPC-OD**, **NU-901-DPPC-OD**, **NU-901-DOPC-SDS**, **NU-901-DPPC-SDS**, **NU-901-DOPC-T**, and **NU-901-DPPC-T** were the same as those of NU-901-A-F.

### 11.7 The Procedure for Loading Pemetrexed (Pem@NU-901)

Pemetrexed solution (1 mL, 10 mg/mL) in water was added into the NU-901 suspension (4 mL, 2.5 mg/mL) in water and stirred at 500 rpm at room temperature for 1 day. The final product was washed two times with 70% ethanol and dispersed in sterile water. The Pem@NU-901 were coated with asolectin and F-127 as described previously to obtain **Pem@NU-901-A-F**.

### 11.8 MOF Degradation Study

An equal amount (1 mg) of NU-901 or NU-901-A-F was separately dispersed in 2 mL of PBS solution (pH = 7.4) in a closed vial and then shaken in a water bath with an oscillator at 37 °C. At different times, 0.1 mL of supernatant was taken after centrifugation. The amount of H_4_TBAPy was measured using a UV–vis spectrophotometer at 394 nm with the help of a calibration curve. The scanned supernatant was then transferred back to the vial for a continued release.

### 11.9 Pemetrexed Release Study

An equal amount (1 mg) of Pem@NU-901 or Pem@NU-901-A-F was separately dispersed in 2 mL of water or PBS solution (pH = 7.4) in a closed vial and then shaken in a water bath with an oscillator at 37 °C. At different times, 0.1 mL of supernatant was taken after centrifugation. The amount of pemetrexed was measured using a UV–vis spectrophotometer at 286 nm with the help of a calibration curve. The scanned supernatant was then transferred back to the vial for continued release.

## Supporting Information Available

General experimental remarks, synthesis, physicochemical characterization, stability analysis of MOFs in PBS, analysis of pemetrexed release, in vitro characterization, and calculation of BET areas using BETSI approach (PDF)
Supplementary Movie 1: Structured illumination microscopy (SIM) video of A549 cells exposed to NU-901-A-F for 16 h (AVI)
Supplementary Movie 2: Structured illumination microscopy (SIM) video of A549 cells exposed to NU-901-A-F for 16 h (AVI)

## Supplementary Material

cm3c02954_si_001.pdf

cm3c02954_si_002.avi

cm3c02954_si_003.avi

## References

[ref1] Wild World Cancer Report 2014, Stewart B. W., Ed.; International Agency for Research on Cancer, WHO Press, 2014. 39432694

[ref2] Lammers T.; Subr V.; Ulbrich K.; Hennink W. E.; Storm G.; Kiessling F. Polymeric Nanomedicines for Image-Guided Drug Delivery and Tumor-Targeted Combination Therapy. Nano Today 2010, 5, 197–212. 10.1016/j.nantod.2010.05.001.

[ref3] Langer R. S.; Peppas N. A. Present and Future Applications of Biomaterials in Controlled Drug Delivery Systems. Acta Biomater. 1981, 2, 201–214. 10.1016/0142-9612(81)90059-4. 7034798

[ref4] Liechty W. B.; Kryscio D. R.; Slaughter B. V.; Peppas N. A. Polymers for Drug Delivery Systems. Annu. Rev. Chem. Biomol. Eng. 2010, 1, 149–173. 10.1146/annurev-chembioeng-073009-100847. 22432577 PMC3438887

[ref5] Whitehead K. A.; Langer R.; Anderson D. G. Knocking down Barriers: Advances in SiRNA Delivery. Nat. Rev. Drug Discovery 2009, 8, 129–138. 10.1038/nrd2742. 19180106 PMC7097568

[ref6] Hoskins B. F.; Robson R. Infinite Polymeric Frameworks Consisting of Three Dimensionally Linked Rod-like Segments. J. Am. Chem. Soc. 1989, 111, 5962–5964. 10.1021/ja00197a079.

[ref7] Moghadam P. Z.; Li A.; Wiggin S. B.; Tao A.; Maloney A. G. P.; Wood P. A.; Ward S. C.; Fairen-Jimenez D. Development of a Cambridge Structural Database Subset: A Collection of Metal – Organic Frameworks for Past, Present, and Future. Chem. Mater. 2017, 29, 2618–2625. 10.1021/acs.chemmater.7b00441.

[ref8] Moghadam P. Z.; Li A.; Liu W.-X.; Bueno-Perez R.; Wang S.-D.; Wiggin S. B.; et al. P. A. W. and D. F.-J. Targeted Classification of Metal-Organic Frameworks in the Cambridge Structural Database (CSD). Chem. Sci. 2020, 11, 8373–8387. 10.1039/d0sc01297a. 33384860 PMC7690317

[ref9] Ma S.; Zhou H. C. Gas Storage in Porous Metal-Organic Frameworks for Clean Energy Applications. Chem. Commun. 2010, 46, 44–53. 10.1039/B916295J. 20024292

[ref10] Li J. R.; Sculley J.; Zhou H. C. Metal-Organic Frameworks for Separations. Chem. Rev. 2012, 112, 869–932. 10.1021/cr200190s. 21978134

[ref11] Zhu L.; Liu X. Q.; Jiang H. L.; Sun L. B. Metal-Organic Frameworks for Heterogeneous Basic Catalysis. Chem. Rev. 2017, 117, 8129–8176. 10.1021/acs.chemrev.7b00091. 28541694

[ref12] Kreno L. E.; Leong K.; Farha O. K.; Allendorf M.; Van Duyne R. P.; Hupp J. T. Metal-Organic Framework Materials as Chemical Sensors. Chem. Rev. 2012, 112, 1105–1125. 10.1021/cr200324t. 22070233

[ref13] Horcajada P.; Gref R.; Baati T.; Allan P. K.; Maurin G.; Couvreur P.; Férey G.; Morris R. E.; Serre C. Metal–Organic Frameworks in Biomedicine. Chem. Rev. 2012, 112 (2), 1232–1268. 10.1021/cr200256v. 22168547

[ref14] Huxford R. C.; Della Rocca J.; Lin W. Metal-Organic Frameworks as Potential Drug Carriers. Curr. Opin. Chem. Biol. 2010, 14, 262–268. 10.1016/j.cbpa.2009.12.012. 20071210 PMC2847625

[ref15] Xiao B.; Wheatley P. S.; Zhao X.; Fletcher A. J.; Fox S.; Rossi A. G.; Megson I. L.; Bordiga S.; Regli L.; Thomas K. M.; Morris R. E. High-Capacity Hydrogen and Nitric Oxide Adsorption and Storage in a Metal-Organic Framework. J. Am. Chem. Soc. 2007, 129, 1203–1209. 10.1021/ja066098k. 17263402

[ref16] Horcajada P.; Serre C.; Vallet-Regí M.; Sebban M.; Taulelle F.; Férey G. Metal-Organic Frameworks as Efficient Materials for Drug Delivery. Angew. Chemie - Int. Ed. 2006, 45, 5974–5978. 10.1002/anie.200601878. 16897793

[ref17] Bernini M. C.; Fairen-Jimenez D.; Pasinetti M.; Ramirez-Pastor A. J.; Snurr R. Q. Screening of Bio-Compatible Metal-Organic Frameworks as Potential Drug Carriers Using Monte Carlo Simulations. J. Mater. Chem. B 2014, 2, 766–774. 10.1039/C3TB21328E. 32261308

[ref18] Teplensky M. H.; Fantham M.; Li P.; Wang T. C.; Mehta J. P.; Young L. J.; Moghadam P. Z.; Hupp J. T.; Farha O. K.; Kaminski C. F.; Fairen-Jimenez D. Temperature Treatment of Highly Porous Zirconium-Containing Metal-Organic Frameworks Extends Drug Delivery Release. J. Am. Chem. Soc. 2017, 139, 7522–7532. 10.1021/jacs.7b01451. 28508624

[ref19] He C.; Lu K.; Liu D.; Lin W. Nanoscale Metal-Organic Frameworks for the Co-Delivery of Cisplatin and Pooled SiRNAs to Enhance Therapeutic Efficacy in Drug-Resistant Ovarian Cancer Cells. J. Am. Chem. Soc. 2014, 136, 5181–5184. 10.1021/ja4098862. 24669930 PMC4210117

[ref20] Orellana-Tavra C.; Marshall R. J.; Baxter E. F.; Lázaro I. A.; Tao A.; Cheetham A. K.; Forgan R. S.; Fairen-Jimenez D. Drug Delivery and Controlled Release from Biocompatible Metal-Organic Frameworks Using Mechanical Amorphization. J. Mater. Chem. B 2016, 4, 7697–7707. 10.1039/C6TB02025A. 32263827

[ref21] Teplensky M. H.; Fantham M.; Poudel C.; Hockings C.; Lu M.; Guna A.; Aragones-Anglada M.; Moghadam P. Z.; Li P.; Farha O. K.; Bernaldo de Quirós Fernández S.; Richards F. M.; Jodrell D. I.; Kaminski Schierle G.; Kaminski C. F.; Fairen-Jimenez D. A Highly Porous Metal-Organic Framework System to Deliver Payloads for Gene Knockdown. Chem. 2019, 5, 2926–2941. 10.1016/j.chempr.2019.08.015.

[ref22] Orellana-Tavra C.; Haddad S.; Marshall R. J.; Abánades Lázaro I.; Boix G.; Imaz I.; Maspoch D.; Forgan R. S.; Fairen-Jimenez D. Tuning the Endocytosis Mechanism of Zr-Based Metal-Organic Frameworks through Linker Functionalization. ACS Appl. Mater. Interfaces 2017, 9, 35516–35525. 10.1021/acsami.7b07342. 28925254 PMC5663390

[ref23] Haddad S.; Lazaro I. A.; Fantham M.; Mishra A.; Silvestre-Albero J.; Osterrieth J. W. M.; Kaminski G. S.; Kaminski C. F.; Forgan R.; Fairen-Jimenez D. Design of a Functionalized Metal–Organic Framework System for Enhanced Targeted Delivery to Mitochondria. J. Am. Chem. Soc. 2020, 142 (14), 6661–6674. 10.1021/jacs.0c00188. 32182066 PMC7146860

[ref24] Orellana-Tavra C.; Baxter E. F.; Tian T.; Bennett T. D.; Slater N. K. H.; Cheetham A. K.; Fairen-Jimenez D. Amorphous Metal-Organic Frameworks for Drug Delivery. Chem. Commun. 2015, 51, 13878–13881. 10.1039/C5CC05237H. 26213904

[ref25] Abánades Lázaro I.; Haddad S.; Rodrigo-Muñoz J. M.; Marshall R. J.; Sastre B.; Del Pozo V.; Fairen-Jimenez D.; Forgan R. S. Surface-Functionalization of Zr-Fumarate MOF for Selective Cytotoxicity and Immune System Compatibility in Nanoscale Drug Delivery. ACS Appl. Mater. Interfaces 2018, 10, 31146–31157. 10.1021/acsami.8b11652. 30136840

[ref26] Abánades Lázaro I.; Haddad S.; Sacca S.; Orellana-Tavra C.; Fairen-Jimenez D.; Forgan R. S. Selective Surface PEGylation of UiO-66 Nanoparticles for Enhanced Stability, Cell Uptake, and PH-Responsive Drug Delivery. Chem. 2017, 2, 561–578. 10.1016/j.chempr.2017.02.005. 28516168 PMC5421152

[ref27] Chen X.; Zhuang Y.; Rampal N.; Hewitt R.; Divitini G.; O’Keefe C. A.; Liu X.; Whitaker D. J.; Wills J. W.; Jugdaohsingh R.; Powell J. J.; Yu H.; Grey C. P.; Scherman O. A.; Fairen-Jimenez D. Formulation of Metal-Organic Framework-Based Drug Carriers by Controlled Coordination of Methoxy PEG Phosphate: Boosting Colloidal Stability and Redispersibility. J. Am. Chem. Soc. 2021, 143, 13557–13572. 10.1021/jacs.1c03943. 34357768 PMC8414479

[ref28] Ettlinger R.; Lächelt U.; Gref R.; Horcajada P.; Lammers T.; Serre C.; Couvreur P.; Morris R. E.; Wuttke S. Toxicity of Metal-Organic Framework Nanoparticles: From Essential Analyses to Potential Applications. Chem. Soc. Rev. 2022, 51, 464–484. 10.1039/D1CS00918D. 34985082

[ref29] Singh N.; Qutub S.; Khashab N. M. Biocompatibility and Biodegradability of Metal Organic Frameworks for Biomedical Applications. J. Mater. Chem. B 2021, 9, 5925–5934. 10.1039/D1TB01044A. 34259304

[ref30] Christodoulou I.; Lyu P.; Soares C. V.; Patriarche G.; Serre C.; Maurin G.; Gref R. Nanoscale Iron-Based Metal–Organic Frameworks: Incorporation of Functionalized Drugs and Degradation in Biological Media. Int. J. Mol. Sci. 2023, 24, 3362 10.3390/ijms24043362. 36834775 PMC9965190

[ref31] Horcajada P.; Gref R.; Baati T.; Allan P. K.; Maurin G.; Couvreur P.; Férey G.; Morris R. E.; Serre C. Metal-Organic Frameworks in Biomedicine. Chem. Rev. 2012, 112, 1232–1268. 10.1021/cr200256v. 22168547

[ref32] Pinto R. V.; Wang S.; Tavares S. R.; Pires J.; Antunes F.; Vimont A.; Clet G.; Daturi M.; Maurin G.; Serre C.; Pinto M. L. Tuning Cellular Biological Functions Through the Controlled Release of NO from a Porous Ti-MOF. Angew. Chemie - Int. Ed. 2020, 59, 5135–5143. 10.1002/anie.201913135. 31951064

[ref33] Bužek D.; Demel J.; Lang K. Zirconium Metal-Organic Framework UiO-66: Stability in an Aqueous Environment and Its Relevance for Organophosphate Degradation. Inorg. Chem. 2018, 57, 14290–14297. 10.1021/acs.inorgchem.8b02360. 30371080

[ref34] Behzadi S.; Serpooshan V.; Tao W.; Hamaly M. A.; Alkawareek M. Y.; Dreaden E. C.; Brown D.; Alkilany A. M.; Farokhzad O. C.; Mahmoudi M. Cellular Uptake of Nanoparticles: Journey inside the Cell. Chem. Soc. Rev. 2017, 46, 4218–4244. 10.1039/C6CS00636A. 28585944 PMC5593313

[ref35] Mosesson Y.; Mills G. B.; Yarden Y. Derailed Endocytosis: An Emerging Feature of Cancer. Nat. Rev. Cancer 2008, 8, 835–850. 10.1038/nrc2521. 18948996

[ref36] Farha O. K.; Vermeulen N. A.; Hupp J. T.; Howarth A. J.; Wang T. C.; Peters A. W. Best Practices for the Synthesis, Activation, and Characterization of Metal–Organic Frameworks. Chem. Mater. 2017, 29, 26–39. 10.1021/acs.chemmater.6b02626.

[ref37] McGuire C. V.; Forgan R. S. The Surface Chemistry of Metal-Organic Frameworks. Chem. Commun. 2015, 51, 5199–5217. 10.1039/C4CC04458D. 25116412

[ref38] Simon-Yarza T.; Mielcarek A.; Couvreur P.; Serre C. Nanoparticles of Metal-Organic Frameworks: On the Road to In Vivo Efficacy in Biomedicine. Adv. Mater. 2018, 30 (37), 1707365 10.1002/adma.201707365. 29876985

[ref39] Bai F.; Wang D.; Huo Z.; Chen W.; Liu L.; Liang X.; Chen C.; Wang X.; Peng Q.; Li Y. A Versatile Bottom-up Assembly Approach to Colloidal Spheres from Nanocrystals. Angew. Chemie - Int. Ed. 2007, 46, 6650–6653. 10.1002/anie.200701355. 17661295

[ref40] Yang J.; Chen X.; Li Y.; Zhuang Q.; Liu P.; Gu J. Zr-Based MOFs Shielded with Phospholipid Bilayers: Improved Biostability and Cell Uptake for Biological Applications. Chem. Mater. 2017, 29, 4580–4589. 10.1021/acs.chemmater.7b01329.

[ref41] Chen X.; Argandona S. M.; Melle F.; Rampal N.; Fairen-Jimenez D. Advances in Surface Functionalization of Next-Generation Metal-Organic Frameworks for Biomedical Applications: Design, Strategies, and Prospects. Chem. 2024, 10, 504–543. 10.1016/j.chempr.2023.09.016.

[ref42] Deria P.; Bury W.; Hupp J. T.; Farha O. K. Versatile Functionalization of the NU-1000 Platform by Solvent-Assisted Ligand Incorporation. Chem. Commun. 2014, 50, 1965–1968. 10.1039/c3cc48562e. 24406797

[ref43] Li Y. F.; Yue P. P.; Hao X.; Bian J.; Ren J. L.; Peng F.; Sun R. C. Comparison of Emulsifying Capacity of Two Hemicelluloses from Moso Bamboo in Soy Oil-in-Water Emulsions. RSC Adv. 2020, 10, 4657–4663. 10.1039/C9RA08636F. 35495257 PMC9049161

[ref44] Wang S.; Chen Y.; Wang S.; Li P.; Mirkin C. A.; Farha O. K. DNA-Functionalized Metal-Organic Framework Nanoparticles for Intracellular Delivery of Proteins. J. Am. Chem. Soc. 2019, 141, 2215–2219. 10.1021/jacs.8b12705. 30669839 PMC8212418

[ref45] Johns A.; Morris S.; Edwards K.; Quirino R. L. Asolectin from Soybeans as a Natural Compatibilizer for Cellulose-Reinforced Biocomposites from Tung Oil. J. Appl. Polym. Sci. 2015, 132 (17), 1–9. 10.1002/app.41833.25866416

[ref46] Wu Z.; Guo C.; Liang S.; Zhang H.; Wang L.; Sun H.; Yang B. A Pluronic F127 Coating Strategy to Produce Stable Up-Conversion NaYF 4: Yb,Er(Tm) Nanoparticles in Culture Media for Bioimaging. J. Mater. Chem. 2012, 22, 18596–18602. 10.1039/c2jm33626j.

[ref47] Chernova T.; Sun X. M.; Powley I. R.; Galavotti S.; Grosso S.; Murphy F. A.; Miles G. J.; Cresswell L.; Antonov A. V.; Bennett J.; Nakas A.; Dinsdale D.; Cain K.; Bushell M.; Willis A. E.; MacFarlane M. Molecular Profiling Reveals Primary Mesothelioma Cell Lines Recapitulate Human Disease. Cell Death Differ 2016, 23, 1152–1164. 10.1038/cdd.2015.165. 26891694 PMC4946883

[ref48] Obacz J.; Yung H.; Shamseddin M.; Linnane E.; Liu X.; Azad A. A.; Rassl D. M.; Fairen-Jimenez D.; Rintoul R. C.; Nikolić M. Z.; Marciniak S. J. Biological Basis for Novel Mesothelioma Therapies. Br. J. Cancer 2021, 125, 1039–1055. 10.1038/s41416-021-01462-2. 34226685 PMC8505556

[ref49] Herbst R. S.; Morgensztern D.; Boshoff C. The Biology and Management of Non-Small Cell Lung Cancer. Nature 2018, 553, 446–454. 10.1038/nature25183. 29364287

[ref50] Vogelzang N. J.; Rusthoven J. J.; Symanowski J.; Denham C.; Kaukel E.; Ruffie P.; Gatzemeier U.; Boyer M.; Emri S.; Manegold C.; Niyikiza C.; Paoletti P. Phase III Study of Pemetrexed in Combination with Cisplatin versus Cisplatin Alone in Patients with Malignant Pleural Mesothelioma. J. Clin. Oncol. 2003, 21, 2636–2644. 10.1200/JCO.2003.11.136. 12860938

[ref51] Webber T. E.; Liu W. G.; Desai S. P.; Lu C. C.; Truhlar D. G.; Penn R. L. Role of a Modulator in the Synthesis of Phase-Pure NU-1000. ACS Appl. Mater. Interfaces 2017, 9, 39342–39346. 10.1021/acsami.7b11348. 29090902

[ref52] Garibay S. J.; Iordanov I.; Islamoglu T.; DeCoste J. B.; Farha O. K. Synthesis and Functionalization of Phase-Pure NU-901 for Enhanced CO2 Adsorption: The Influence of a Zirconium Salt and Modulator on the Topology and Phase Purity. CrystEngcomm 2018, 20, 7066–7070. 10.1039/C8CE01454J.

[ref53] Mondloch J. E.; Bury W.; Fairen-Jimenez D.; Kwon S.; Demarco E. J.; Weston M. H.; Sarjeant A. A.; Nguyen S. T.; Stair P. C.; Snurr R. Q.; Farha O. K.; Hupp J. T. Vapor-Phase Metalation by Atomic Layer Deposition in a Metal-Organic Framework. J. Am. Chem. Soc. 2013, 135, 10294–10297. 10.1021/ja4050828. 23829224

[ref54] Verma P. K.; Huelsenbeck L.; Nichols A. W.; Islamoglu T.; Heinrich H.; Machan C. W.; Giri G. Controlling Polymorphism and Orientation of NU-901/NU-1000 Metal-Organic Framework Thin Films. Chem. Mater. 2020, 32, 10556–10565. 10.1021/acs.chemmater.0c03539.

[ref55] Xu M.; Meng S. S.; Cai P.; Gu Y. H.; Yan T. A.; Yan T. H.; Zhang Q. H.; Gu L.; Liu D. H.; Zhou H. C.; Gu Z. Y. Homogeneously Mixing Different Metal-Organic Framework Structures in Single Nanocrystals through Forming Solid Solutions. ACS Cent. Sci. 2022, 8, 184–191. 10.1021/acscentsci.1c01344. 35233451 PMC8874727

[ref56] Li P.; Klet R. C.; Moon S. Y.; Wang T. C.; Deria P.; Peters A. W.; Klahr B. M.; Park H. J.; Al-Juaid S. S.; Hupp J. T.; Farha O. K. Synthesis of Nanocrystals of Zr-Based Metal-Organic Frameworks with Csq-Net: Significant Enhancement in the Degradation of a Nerve Agent Simulant. Chem. Commun. 2015, 51, 10925–10928. 10.1039/C5CC03398E. 26063329

[ref57] Webber T. E.; Desai S. P.; Combs R. L.; Bingham S.; Lu C. C.; Penn R. L. Size Control of the MOF NU-1000 through Manipulation of the Modulator/Linker Competition. Cryst. Growth Des. 2020, 20, 2965–2972. 10.1021/acs.cgd.9b01590.

[ref58] Wang T. C.; Vermeulen N. A.; Kim I. S.; Martinson A. B. F.; Fraser Stoddart J.; Hupp J. T.; Farha O. K. Scalable Synthesis and Post-Modification of a Mesoporous Metal-Organic Framework Called NU-1000. Nat. Protoc. 2016, 11 (1), 149–162. 10.1038/nprot.2016.001. 26678084

[ref59] Orellana-Tavra C.; Mercado S. A.; Fairen-Jimenez D. Endocytosis Mechanism of Nano Metal-Organic Frameworks for Drug Delivery. Adv. Healthc. Mater. 2016, 5, 2261–2270. 10.1002/adhm.201600296. 27385477

[ref60] Torchilin V. P. Multifunctional, stimuli-sensitive nanoparticulate systems for drug delivery. Nat. Rev. Drug Discov. 2014, 13 (11), 813–827. 10.1038/nrd4333. 25287120 PMC4489143

[ref61] Blanco E.; Shen H.; Ferrari M. Principles of Nanoparticle Design for Overcoming Biological Barriers to Drug Delivery. Nat. Biotechnol. 2015, 33, 941–951. 10.1038/nbt.3330. 26348965 PMC4978509

[ref62] Islamoglu T.; Otake K. I.; Li P.; Buru C. T.; Peters A. W.; Akpinar I.; Garibay S. J.; Farha O. K. Revisiting the Structural Homogeneity of NU-1000, a Zr-Based Metal-Organic Framework. CrystEngcomm 2018, 20, 5913–5918. 10.1039/C8CE00455B.

[ref63] Osterrieth J. W. M.; Wright D.; Noh H.; Kung C. W.; Vulpe D.; Li A.; Park J. E.; Van Duyne R. P.; Moghadam P. Z.; Baumberg J. J.; Farha O. K.; Fairen-Jimenez D. Core-Shell Gold Nanorod@Zirconium-Based Metal-Organic Framework Composites as in Situ Size-Selective Raman Probes. J. Am. Chem. Soc. 2019, 141, 3893–3900. 10.1021/jacs.8b11300. 30707577

[ref64] Chen X.; Jagadesan P.; Valandro S.; Hupp J. T.; Schanze K. S.; Goswami S. Identifying the Polymorphs of Zr-Based Metal-Organic Frameworks via Time-Resolved Fluorescence Imaging. ACS Mater. Lett. 2022, 4, 370–377. 10.1021/acsmaterialslett.1c00754.

[ref65] Osterrieth J. W. M.; Rampersad J.; Madden D.; Rampal N.; Skoric L.; Connolly B.; Allendorf M. D.; Stavila V.; Snider J. L.; Fairen-Jimenez D. How Reproducible Are Surface Areas Calculated from the BET Equation?. Adv. Mater. 2022, 34, 202201502. 10.1002/adma.202201502 35603497

[ref66] Marshall C. R.; Staudhammer S. A.; Brozek C. K. Size Control over Metal-Organic Framework Porous Nanocrystals. Chem. Sci. 2019, 10, 9396–9408. 10.1039/C9SC03802G. 32055316 PMC6979335

[ref67] Fan H.; Yang K.; Boye D. M.; Sigmon T.; Malloy K. J.; Xu H.; López G. P.; Brinker C. J. Self-Assembly of Ordered, Robust, Three-Dimensional Gold Nanocrystal/ Silica Arrays. Science 2004, 304, 567–571. 10.1126/science.1095140. 15105495

[ref68] Salabat A.; Eastoe J.; Mutch K. J.; Tabor R. F. Tuning Aggregation of Microemulsion Droplets and Silica Nanoparticles Using Solvent Mixtures. J. Colloid Interface Sci. 2008, 318, 244–251. 10.1016/j.jcis.2007.10.050. 18054035

[ref69] Ethayaraja M.; Dutta K.; Muthukumaran D.; Bandyopadhyaya R. Nanoparticle Formation in Water-in-Oil Microemulsions: Experiments, Mechanism, and Monte Carlo Simulation. Langmuir 2007, 23, 3418–3423. 10.1021/la062896c. 17305375

[ref70] Aubery C.; Solans C.; Prevost S.; Gradzielski M.; Sanchez-Dominguez M. Microemulsions as Reaction Media for the Synthesis of Mixed Oxide Nanoparticles: Relationships between Microemulsion Structure, Reactivity, and Nanoparticle Characteristics. Langmuir 2013, 29, 1779–1789. 10.1021/la303817w. 23305179

[ref71] Luo D.; Qin X.; Song Q.; Qiao X.; Zhang Z.; Xue Z.; Liu C.; Mo G.; Wang T. Ordered Superparticles with an Enhanced Photoelectric Effect by Sub-Nanometer Interparticle Distance. Adv. Funct. Mater. 2017, 27 (44), 1701982 10.1002/adfm.201701982.

[ref72] Mejia-Ariza R.; Huskens J. The Effect of PEG Length on the Size and Guest Uptake of PEG-Capped MIL-88A Particles. J. Mater. Chem. B 2016, 4, 1108–1115. 10.1039/C5TB01949D. 32263003

[ref73] Wang Q.; Shen M.; Zhao T.; Xu Y.; Lin J.; Duan Y.; Gu H. Low Toxicity and Long Circulation Time of Polyampholyte-Coated Magnetic Nanoparticles for Blood Pool Contrast Agents. Sci. Rep. 2015, 5, 7774 10.1038/srep07774. 25585607 PMC4293589

[ref74] Gratton S. E. A.; Ropp P. A.; Pohlhaus P. D.; Luft J. C.; Madden V. J.; Napier M.E.; DeSimone J. M. The Effect of Particle Design on Cellular Internalization Pathways. Proc. Natl. Acad. Sci. U. S. A. 2008, 105, 11613–11618. 10.1073/pnas.0801763105. 18697944 PMC2575324

[ref75] Greenhalgh J.; McLeod C.; Bagust A.; Boland A.; Fleeman N.; Dundar Y.; Oyee J.; Dickson R.; Davis H.; Green J.; McKenna E.; Pearson M. Pemetrexed for the Maintenance Treatment of Locally Advanced or Metastatic Non-Small Cell Lung Cancer. Health Technol. Assess 2010, 14, 33–39. 10.3310/hta14suppl2-05. 21047489

[ref76] Zhang M.; Chen Y. P.; Bosch M.; Gentle T.; Wang K.; Feng D.; Wang Z. U.; Zhou H. C. Symmetry-Guided Synthesis of Highly Porous Metal-Organic Frameworks with Fluorite Topology. Angew. Chemie - Int. Ed. 2014, 53, 815–818. 10.1002/anie.201307340. 24218230

[ref77] Orellana-Tavra C.; Köppen M.; Li A.; Stock N.; Fairen-Jimenez D. Biocompatible,Crystalline, and Amorphous Bismuth-Based Metal-Organic Frameworks for Drug Delivery. ACS Appl. Mater. Interfaces 2020, 12, 5633–5641. 10.1021/acsami.9b21692. 31940165

[ref78] Ho V. M.; Lee J.-A. The Effect of Temperature on Gene Silencing by SiRNAs: Implications for Silencing in the Anterior Chamber of the Eye Paul. Bone 2012, 23, 1–7. 10.1016/j.exer.2005.12.003 PMC1489385 16466716

[ref79] Simon J.; Müller J.; Ghazaryan A.; Morsbach S.; Mailänder V.; Landfester K. Protein Denaturation Caused by Heat Inactivation Detrimentally Affects Biomolecular Corona Formation and Cellular Uptake. Nanoscale 2018, 10, 21096–21105. 10.1039/C8NR07424K. 30427359

[ref80] Linnane E.; Haddad S.; Melle F.; Mei Z.; Fairen-Jimenez D. The Uptake of Metal-Organic Frameworks: A Journey into the Cell. Chem. Soc. Rev. 2022, 51, 6065–6086. 10.1039/D0CS01414A. 35770998 PMC9289890

[ref81] Ling S.; Polymenidou M.; Cleveland D. W.; Revyakin A.; Patel R.; Macklin J. J.; Normanno D.; Robert H. A General Method to Improve Fluorophores for Live-Cell and Single-Molecule Microscopy. Nat. Methods 2015, 79, 244–250. 10.1038/nmeth.3256. PMC4344395 25599551

[ref82] Rijnaarts T.; Mejia-Ariza R.; Egberink R. J. M.; Roosmalen W. V.; Huskens J. Metal-Organic Frameworks (MOFs) as Multivalent Materials: Size Control and Surface Functionalization by Monovalent Capping Ligands. Chem. - A Eur. J. 2015, 21, 10296–10301. 10.1002/chem.201501974. 26096150

[ref83] White B.; Banerjee S.; O’Brien S.; Turro N. J.; Herman I. P. Zeta-Potential Measurements of Surfactant-Wrapped Individual Single-Walled Carbon Nanotubes. J. Phys. Chem. Sci. 2007, 111, 13684–13690. 10.1021/jp070853e.

