# Supplementary material for: Enhancing Drug Delivery Efficacy Through Bilayer Coating of Zirconium-Based Metal–Organic Frameworks: Sustained Release and Improved Chemical Stability and Cellular Uptake for Cancer Therapy
Source: Chem Mater. 2024 Apr 11;36(8):3588–603. doi: 10.1021/acs.chemmater.3c02954 (PMC11044268; doi:10.1021/acs.chemmater.3c02954)
Supplement: Supplementary file 1 — cm3c02954_si_001.pdf [file cm3c02954_si_001.pdf]

Supporting Information for:

Enhancing Drug Delivery Efficacy through Bilayer Coating of Zirconium-Based Metal-Organic Frameworks: Sustained Release and Improved Chemical Stability and Cellular Uptake for Cancer Therapy

Xiewen Liu[a], Joanna Obacz[b], Giulia Emanuelli[b], Joseph E. Chambers[b], Susana Abreu[b], Xu Chen[a], Emily Linnane[a], Joshua P. Mehta[c], Andrew E. H. Wheatley[c], Stefan J. Marciniak[b,*] and David Fairen-Jimenez[a,*]

[a] The Adsorption & Advanced Materials Laboratory (A^{2}ML), Department of Chemical Engineering & Biotechnology, University of Cambridge, Philippa Fawcett Drive, Cambridge CB3 0AS, UK

[b] Cambridge Institute for Medical Research, Keith Peters Building, Cambridge Biomedical Campus, University of Cambridge, Cambridge, CB2 0XY, UK

[c] Department of Chemistry, University of Cambridge, Lensfield Road, Cambridge CB2 1EW, UK

*e-mails: sjm20@cam.ac.uk; df334@cam.ac.uk

Table of Contents

S1. General experimental remarks **S2**
S2. Materials and synthesis **S4**
S3. Characterisation **S6**
S4. Stability Analysis of MOFs in PBS **S16**
S5. Analysis of pemetrexed release **S16**
S6. In vitro characterization of nanoMOFs **S17**
S7. Calculation of BET areas using BETSI **S18**
S8. References **S30**

S1. General experimental remarks

Powder X-ray Diffraction (PXRD): Powder X-ray diffraction (PXRD) data were collected on a Bruker D8 DAVINCI diffractometer at 298 K using Cu Kα radiation. The calculated PXRD patterns were produced using the Mercury program and single crystal reflection data.

Gas Uptake: N_2 sorption isotherm measurements were performed on a Micromeritics 3 Flex analyzer at 77K. Samples were degassed under vacuum at 120 °C for 20 hours using the internal turbopump. The Surface areas were calculated using BETSI.

Dynamic Light Scattering: Measurements were recorded on a Zetasizer Nano ZS, (Malvern Instrument Ltd., U.K.) equipped with a He−Ne laser operating at 633 nm at 25 °C.

UV-Vis/Fluorescence Spectroscopy: UV-vis and fluorescence spectra were recorded using a Tecan Spark® Multimode Microplate Reader.

Scanning Electron Microscopy (SEM): The samples for SEM test were coated with Pt for 40 seconds and imagined using an FEI Nova Nano SEM 450.

Transmission electron microscopy (TEM): The samples for TEM test were prepared by dispersing the samples in ethanol using ultrasonication. After that, a small number of suspensions were drop-casted on a copper grid with a carbon support film. TEM micrographs were collected on a Tecnai F20 with an acceleration voltage of 200 kV.

Inductively coupled plasma-optical emission spectroscopy (ICP-OES): ICP-OES was performed using a Perkin Elemer ICP-OES Optima 2100DV. Samples were dispersed in 2 mL of nitric acid and 6 mL of hydrochloric acid (*CAUTION !*) and left to stand at room temperature in the fume cupboard for at least 1 h until all reactions have ceased. After that, samples were heated at 90°C for 10 h to fully digest the sample. The mixture was diluted 750 times before the measurement.

Nuclear Magnetic Resonance (NMR) Spectroscopy: ^{1}H NMR spectroscopy was performed on a 500 MHz DCH Cryoprobe Spectrometer NMR instrument at 25 °C.

Thermogravimetric Analysis (TGA): TGA measurements were carried out using a TA Instruments Q500 Thermogravimetric Analyzer. Measurements were collected from room temperature to 800 °C with a heating rate of 20 °C / min under nitrogen.

Cell culture

A549, an adenocarcinomic alveolar basal epithelial cell line (non-small cell lung cancer) was obtained from ATCC and was maintained in Dulbecco's modified Eagle's medium (DMEM) (Sigma) supplemented with 10% fetal bovine serum (FBS) (Lonza) and penicillin (100 U/mL), streptomycin (100 µg/mL). 3T, a low-passage primary cell line derived from epithelioid pleura mesothelioma, was obtained from MesobanK (Rintoul et al. 2016, PMID: 26467803) and was maintained in RPMI-1640 media (Sigma) supplemented with 10% FBS, l-glutamine (2 mM), penicillin (100 U/mL), streptomycin (100 µg/mL), hEGF (20 ng/mL), hydrocortisone

(1 µg/mL) and heparin (2 µg/mL) as described in (Chernova et al 2016, PMID: 26891694). All cells were cultured in 5% CO2 humidified atmosphere at 37°C.

Cell viability assay

3T and A549 cells were seeded at the concentration of $5x10^4$ cells per well in triplicates in optical bottom 96-well plates. Cells were treated with pemetrexed (Sigma), unloaded MOFs or MOFs loaded with pemetrexed as described above. Cells were incubated for the indicated time points at 37 °C in 5% CO2 and cell viability was determined using a bioluminescence-based commercially available kit, CellTiterGlo® (Promega), following the manufacturer's instructions. The luminescence signal was measured using a Tecan Spark® Multimode Microplate Reader. Data are presented as percentages of surviving cells compared to controls.

Flow Cytometry

3T and A549 cells were seeded at the density of $5x10^5$ cells per well on 6-well plates and allowed to attach for 6 hours before treatment. Cells were exposed to 6 µg/mL of NU-901 or NU901-AF for 16 hours. Before analysis, excess MOFs were washed away from the cells with PBS. Cells were then detached from plates using Trypsin/EDTA solution (Gibco) and collected by centrifugation at 400g for 4 minutes. After 3 washes with PBS cells were resuspended in 500µl 2% FBS in PBS for detection of fluorescence at 405-50 nm via flow cytometry. Data were acquired using a BD LSRFortessaTM Cell Analyzer (BD Biosciences) and the population of interest was gated according to its FSC/SSC criteria. Analysis was conducted with FlowJoTM software (BD Biosciences).

Microscopy

3T primary mesothelioma cells and A549 cells were plated at a density of 1000 cells/cm^2 on 30 mm diameter glass coverslips. 4 hours post seeding, cells were transfected using 1ug of plasmid DNA and a Lipofectamine 2000 (Invitrogen) in a 1:3 ratio. Mammalian expression plasmids encoding mScarlet-I and LAMP1-HaloTag were a kind gift from Jonathon Nixon-Abell (CIMR, Cambridge UK). 2 hours post-transfection, a stock suspension of MOFs was agitated by sonicating water bath, diluted to 10 µg/mL in culture medium, and added to cells. After 16 hours of MOF incubation, cells were washed 3 times with 3 mL of PBS. Untransfected cells were labeled for 15 minutes with 2.5 µg/mL DilC18 (1,1'-Dioctadecyl-3,3,3',3'-Tetramethylindocarbocyanine Perchlorate, Thermofischer, Catalog number: D3911) followed by 3 PBS washes and addition of fresh culture medium prior to imaging. Transfected cells were labeled with JF646 HaloTag ligand [PMID: 25599551] followed by 3 PBS washes and the addition of fresh culture medium prior to imaging. Confocal live-cell imaging was performed on a Zeiss LSM780 confocal microscope with GaAsP detectors using a 63X 1.4NA oil immersion lens. MOFs, DilC18, iScarlet and JF646 were excited at 405 nm, 561 nm, 561 nm and 633 nm wavelengths, and emissions were collected at 410-556 nm, 578-696 nm, 579-650 nm, 652-755 nm, respectively. Super-resolution, live-cell Lattice SIM images were acquired on a Zeiss Elyra7 microscope using a ×63 1.4 NA oil immersion objective. Images were reconstructed using the Zeiss SIM^2 algorithm in three dimensions using the "standard – live" settings with the sectioning set at 92. MOFs and HaloTag-JF646 were excited at 488 nm and 642 nm respectively and emitted light captured simultaneously using an OptoSplit beam splitter and two pco.edge sCMOS cameras filtered

with band-pass 420-480 nm + band-pass 495-550 nm (MOFs) and band-pass 570-620 + long-pass 655 nm (HaloTag-JF646).

S2. Materials and synthesis

All reagents unless otherwise stated were obtained from commercial sources and were used without further purification.

Preparation of 4,4',4'',4'''-(pyrene-1,3,6,8-tetrayl)tetrabenzoate (H_4TBAPy): H_4TBAPy was prepared according to the protocol by Wang et al.[2] Tetrabromopyrene (0.5 g, 0.97 mmol), 4-ethoxycarbonylphenylboronic acid (8.25 g, 42.5 mmol), potassium phosphate tribasic (16.5 g, 77.7 mmol) and tetrakis(triphnylphosphine)- palladium(0) (0.75 g, 0.65 mmol) were added to a 2-necked round bottom flask and purged with N_2 for 1 hour. Degassed dioxane (270 mL) was injected and the solution heated to 90°C for 72 hours before 300 mL of water was added and let the reaction to cool down at room temperature for 1 hour. The yellow solid was collected under Büchner filtration and washed with 100 mL of water (24 mL) and 200 mL of acetone. The product was dissolved in boiling chloroform (300 mL) and filtered, followed by reducing the volume of the collected chloroform solution containing the product with rotor evaporator to 100 mL. The product was precipitated with methanol (300 mL) and isolated under Buchner filtration before being dried at 70 °C for 12 hours in a vacuum oven, giving 4,4',4'',4'''-(pyrene-1,3,6,8-tetrayl)tetrabenzoate, a yellow solid.

4,4',4'',4'''-(pyrene-1,3,6,8-tetrayl)tetrabenzoate (4.5 g) was then suspended in dioxane (500 mL) and 400 mL of aqueous potassium hydroxide solution (KOH, 7.1 g) was injected. The mixture was stirred vigorously and refluxed for 20 hours, producing a clear solution. Let the solution cool down to room temperature and slowly add concentrated HCl (37%, 12 M) until the solution has a pH = 1 in an ice bath. A yellow precipitate was observed after acidification and the reaction was stirred for an additional 1 hour. The yellow precipitate was collected under Büchner filtration, dried, and suspended in 200 mL of water and sonicated for 1 h to ensure all the salt impurities generated in the neutralization were fully dissolved. The product was filtered and washed with 100 mL of water followed by dissolving it in 100 mL of DMF at 120°C, and filtering while it is hot (CAUTION, please wear heat-insulating gloves). The solution was cooled down to room temperature and 300 mL of dichloromethane were added while stirring (DCM) to obtain yellow precipitate. The yellow solid was collected under filtration and washed with 100 mL of DCM and dried under vacuum at 120°C for 36 hours, yielding 3.7 g of product (bright yellow solid). ^{1}H NMR (500 MHz, DMSO-d6) δ 13.12 (s, 4H), 8.22 (s, 4H), 8.18 (d, J = 8.0 Hz, 8H), 8.10 (s, 2H), 7.88 (d, J = 8.0 Hz, 8H).

Sterile Synthesis of MOFs: To ensure the sterility of MOFs for cellular studies, all solution of reactants was filtered with 0.2 um PTFE filters and all apparatus such as vials and stir bars were autoclaved at 120 °C. All the centrifuge tubes were sterile as received during the washing steps, and 70 % ethanol and sterile water were both applied in the washing step.

NU-901 synthesis (~150 nm): $ZrOCl_2{\cdot}8H_2O$ (484 mg, 2.7 mmol), 4-aminobenzoic acid (1050 mg, 7.7 mmol), and 800 μL of TFA were dissolved in DMF (40 mL) to obtain Solution

1. The 4ABA can be replaced by either the same mole of BA or B4CA. H_4TBAPy (100 mg, 0.146 mmol) was then dissolved in DMF (40 mL) in a separate vial to obtain Solution 2. Solution 1 was mixed with Solution 2 in a 100 mL threaded vial. The resultant mixture was incubated in an oil bath at 140 °C and 700 revolutions per minute (rpm) for 50 minutes. The resulting material was isolated by centrifugation and washed thrice with DMF, and thrice with ethanol. The final product was re-dispersed in 70 % ethanol for in vitro studies.

NU-1000 synthesis (~2 μm): $ZrOCl_2 \cdot 8H_2O$ (484 mg, 1.5 mmol), 4-aminobenzoic acid (5 g, 36.5 mmol), and 800 μL of TFA were dissolved in DMF (40 mL) to obtain Solution 1. The 4ABA can be replaced by either the same mole of BA or B4CA. H_4TBAPy (100 mg, 0.146 mmol) was then dissolved in DMF (40 mL) in a separate vial to obtain Solution 2. Solution 1 was mixed with Solution 2 in a 100 mL threaded vial. The resultant mixture was incubated in an oil bath at 140 °C and 700 revolutions per minute (rpm) for 50 minutes. The resulting material was isolated by centrifugation and washed thrice with DMF, and thrice with acetone. The final product was re-dispersed in ethanol for further.

The procedure for coating asolectin to NU-901 (NU-901-A): Asolectin solution (1 mL, 25 mg/mL in $CHCl_3$) was added into the NU-901 suspension (10 mL, 2 mg/mL in $CHCl_3$). After stirring (800 rpm) at room temperature for 4 h, the reaction mixture was washed thrice with $CHCl_3$ under centrifugation at 15,000 rpm to remove unreacted reagents. The resultant NU-901-A should disperse well in chloroform but aggregate to visible flakes in water due to its strong hydrophobicity. The methods for synthesis of **NU-901-DOPC** and **NU-901-DPPC** were same as that of NU-901-A.

The procedure for coating F-127 to NU-901-A (NU-901-A-F)：NU-901-A suspension (1 mL, 2.5 mg/mL) in $CHCl_3$ were mixed with the solution of F-127 (20 mL, 2 mg/mL) in water. The system was then emulsified by ultrasonic treatment for 5 minutes, followed by rapid stirring at 1500 rpm for 10 minutes in a capped vial. The cap was then removed to allow the removal of $CHCl_3$ to obtain NU-901-A-F at room temperature in a fumehood over 12 hours. The final product was washed trice with 70 % ethanol and dispersed in sterile water. The methods for synthesis of **NU-901-DOPC-CTAB**, **NU-901-DPPC-CTAB, NU-901-DOPC-OD**, **NU-901-DPPC-OD**, **NU-901-DOPC-SDS**, **NU-901-DPPC-SDS**, **NU-901-DOPC-T** and **NU-901-DPPC-T** were the same as that of NU-901-A-F.

The procedure for loading pemetrexed (Pem@NU-901)：Pemetrexed solution (1 mL, 10mg/mL) in water was added into the NU-901 suspension (4 mL, 2.5 mg/mL) in water and stirred at 500 rpm at room temperature for 1 day. The final product was washed two times with 70 % ethanol and dispersed in sterile water. The Pem@NU-901 were coated with asolectin and F-127 as described previously to obtain **Pem@NU-901-A-F**.

MOF degradation study. An equal amount (1 mg) of NU-901 or NU-901-A-F was separately dispersed in 2 mL of PBS solution (pH = 7.4) in a closed vial, and then shaken in a water bath with an oscillator at 37 °C. At different times, 0.1 mL of supernatant was taken after centrifugation. The amount of H_4TBAPy was measured using a UV-vis

spectrophotometer at 394 nm with the help of a calibration curve. The scanned supernatant was then transferred back to the vial for continued release.

Pemetrexed release study. An equal amount (1 mg) of Pem@NU-901 or Pem@NU-901-A-F was separately dispersed in 2 mL of water or PBS solution (pH = 7.4) in a closed vial, and then shaken in a water bath with an oscillator at 37 °C. At different times, 0.1 mL of supernatant was taken after centrifugation. The amount of pemetrexed was measured using a UV-vis spectrophotometer at 286 nm with the help of a calibration curve. The scanned supernatant was then transferred back to the vial for continued release.

S3. Characterization

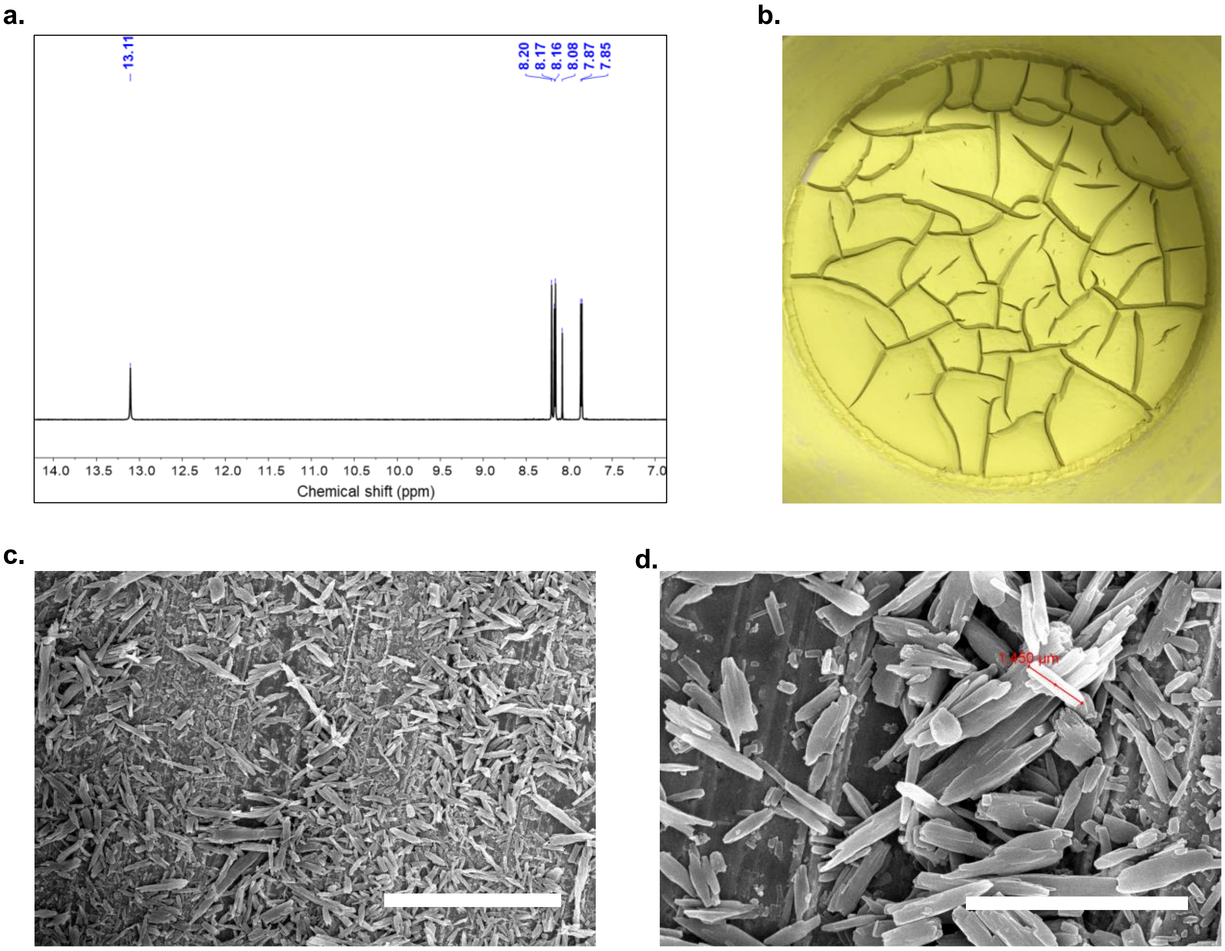

Figure S1. (a) ^{1}H NMR spectrum of synthesized H_4TBAPy in DMSO-d_6. The positions of the measured ^{1}H NMR peaks match with the positions reported in literature[2] [δ = 13.12 (s, 4H), 8.22 (s, 4H), 8.18 (d, J = 8.0 Hz, 8H), 8.10 (s, 2H), 7.88 (d, J = 8.0 Hz, 8H)]. **(b)** A photo of the synthesised yellow H_4TBAPy linker on top of the filter. SEM images of synthesised H_4TBAPy linkers with **(c)** lower magnification (3,000 x, scale bar = 20 µm) and **(d)** higher magnification (10,000 x, scale bar = 5 µm).

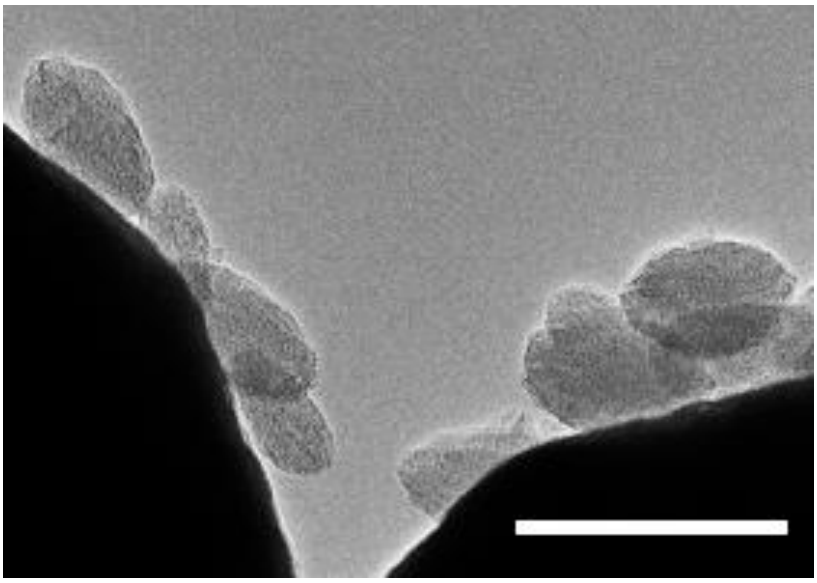

Figure S2. A TEM image of the synthesised nanoNU-901. Scale bar = 200 nm.

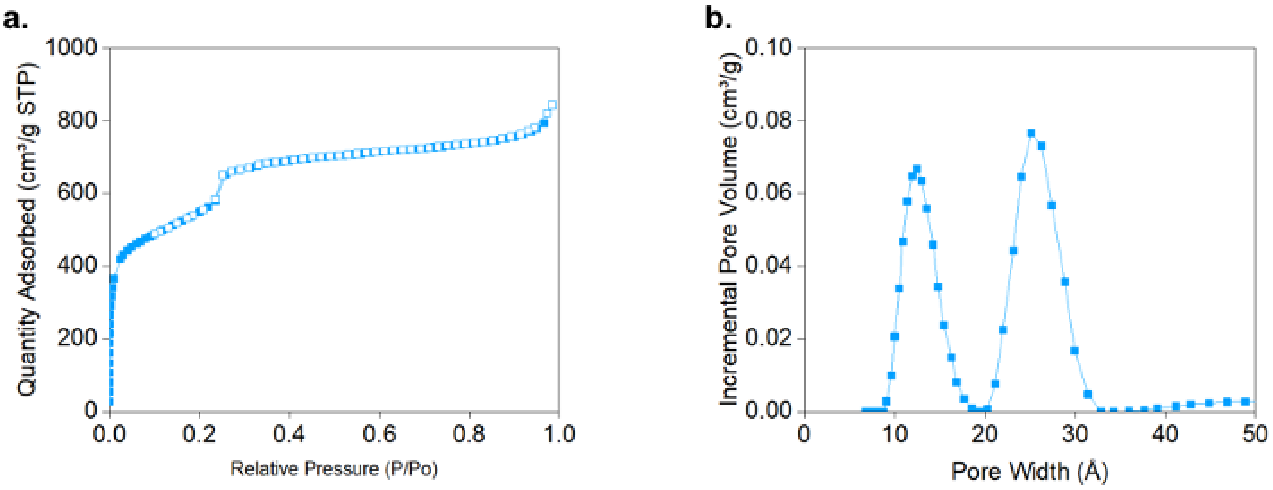

Figure S3. (a) N_2 adsorption isotherm at 77 K of synthesised NU-901 and **(b)** the pore size distribution obtained with the nonlocal density functional theory (NLDFT) method.

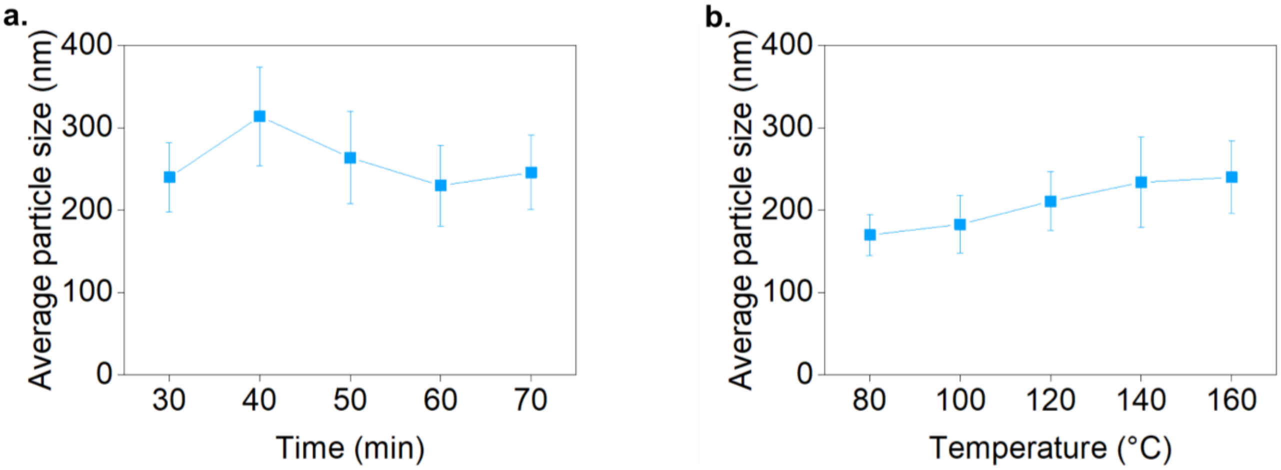

Figure S4. The effects of **(a)** synthesis time and **(b)** temperature on the average particle size of NU-901.

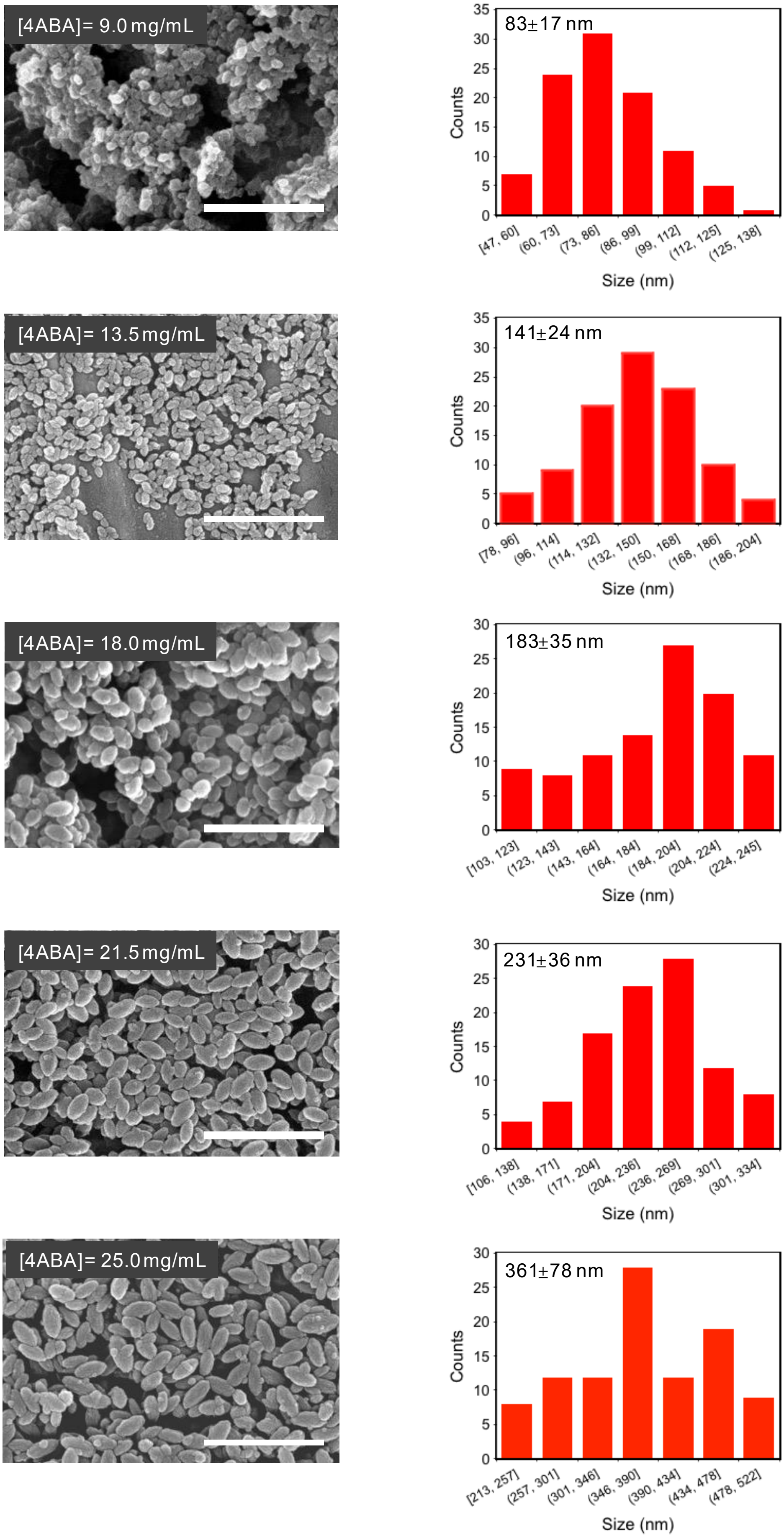

Figure S5. SEM images and particle size analysis (n = 100 measured with ImageJ) of NU-901 in different sizes. With the addition of more 4ABA, the size of the MOF particles increases. Scale bar = 1 μm.

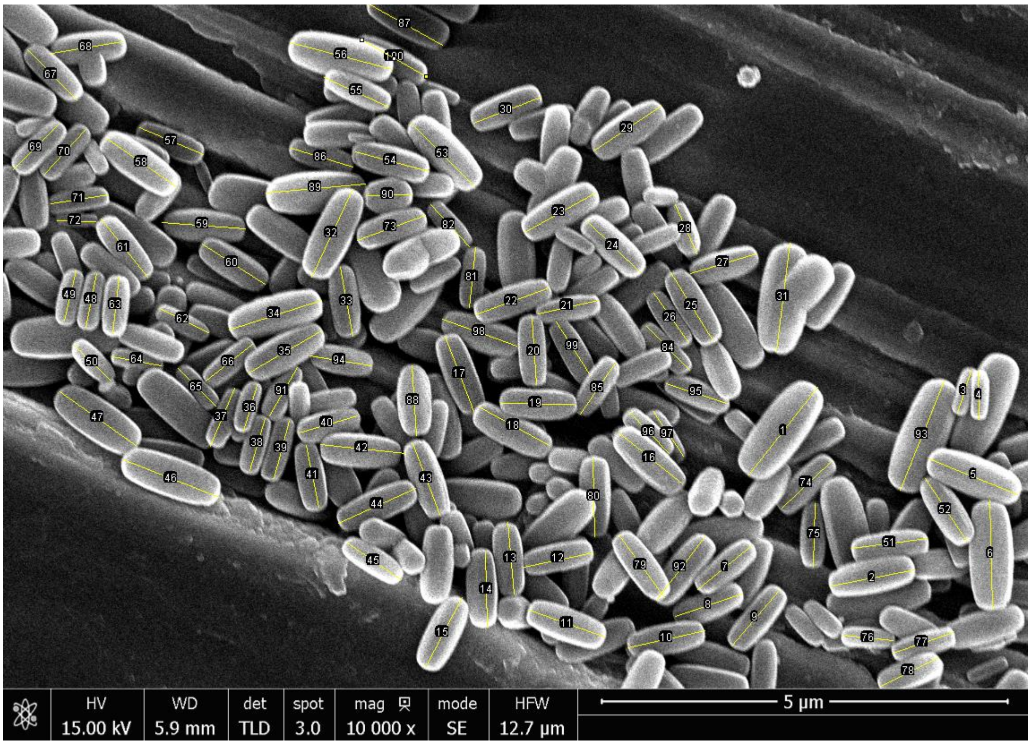

Figure 1. Example of using ImageJ software to acquire mean particle size distribution from an SEM image. The mean particle size is defined via averaging the length of 100 MOF particles from this SEM image using ImageJ and Excel, and the standard deviation (SD) is calculated using Excel.

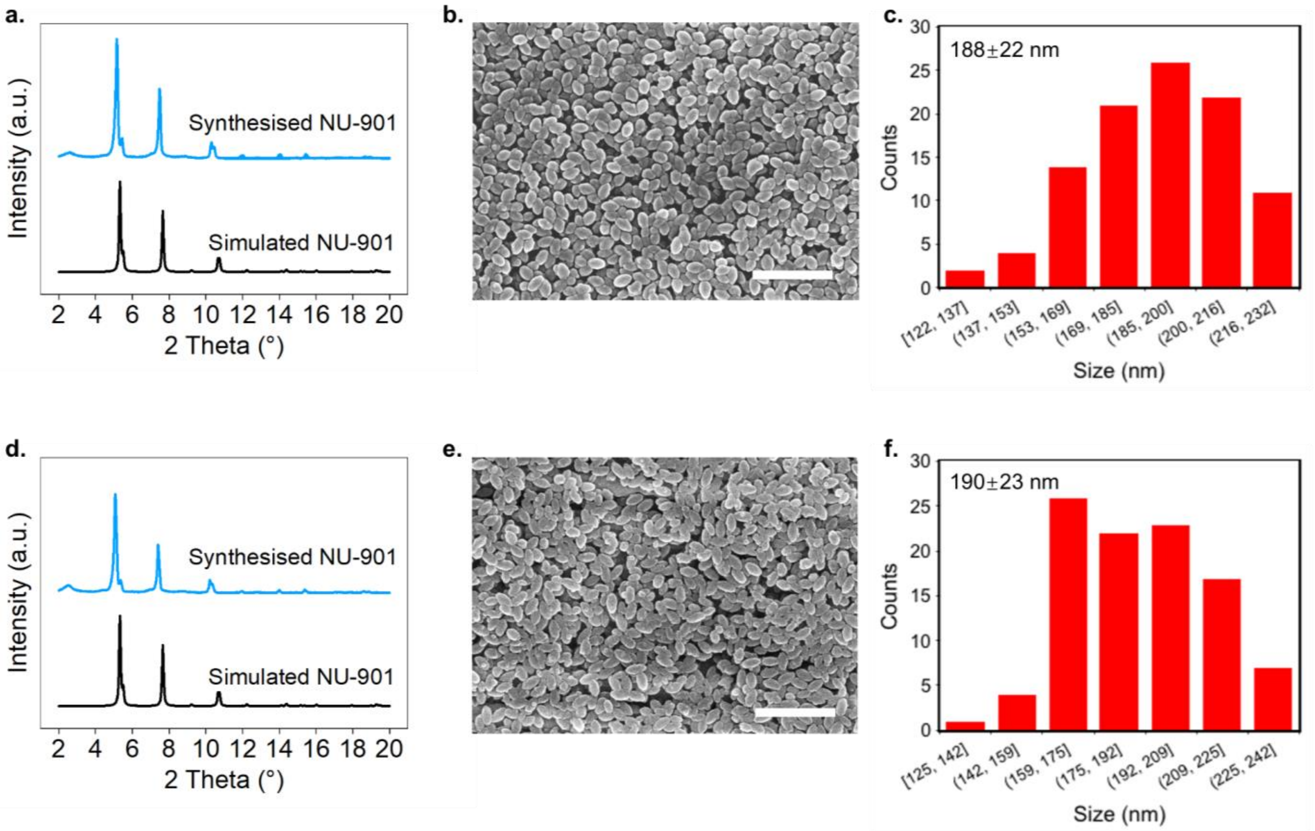

Figure S6. (**a**) PXRD pattern and (**b**) SEM of synthesised NU-901 using biphenyl-4-carboxylic acid (B4CA); (**c**) PXRD pattern and (**d**) SEM of synthesised NU-901 using benzoic acid (BA). Scale bars = 1 µm

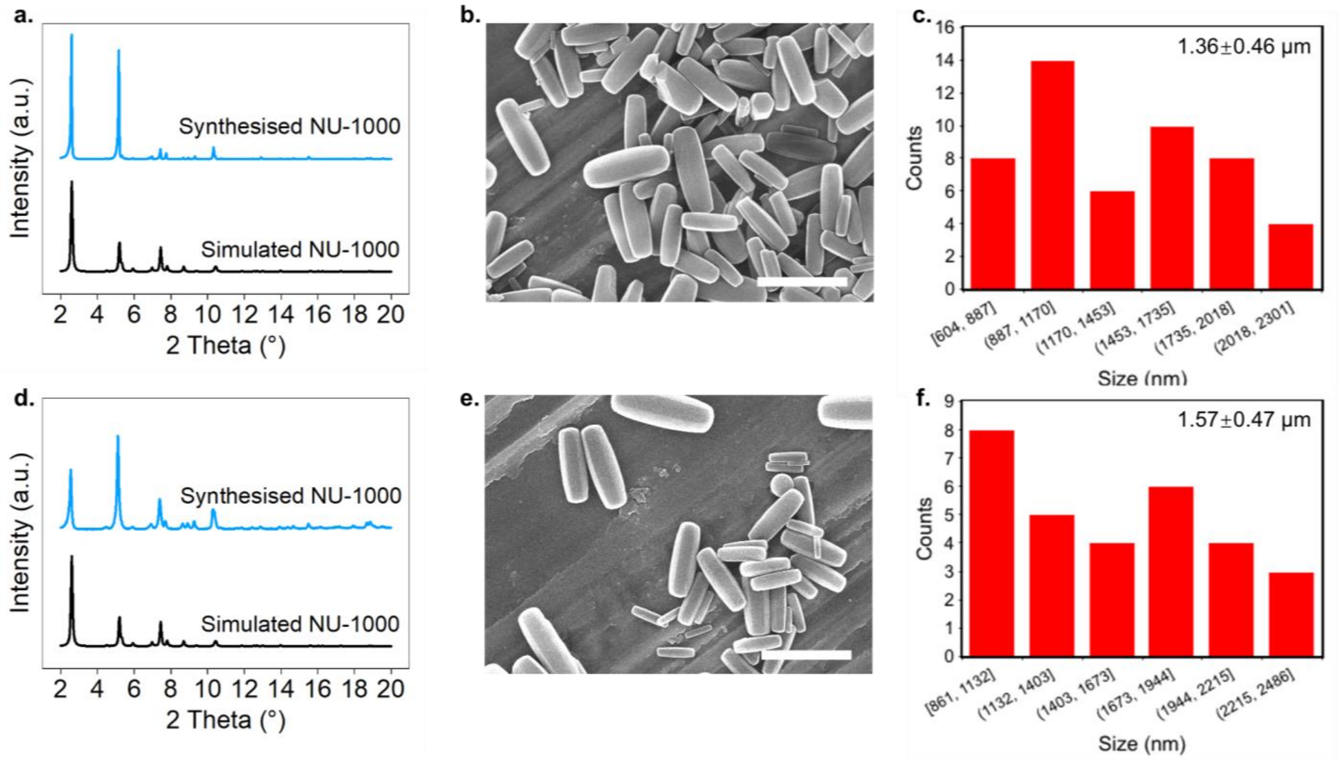

Figure S7. (**a**) PXRD pattern and (**b**) SEM of synthesised NU-1000 using biphenyl-4-carboxylic acid (B4CA); (**c**) PXRD pattern and (**d**) SEM of synthesised NU-1000 using benzoic acid (BA). Scale bars = 2 μm.

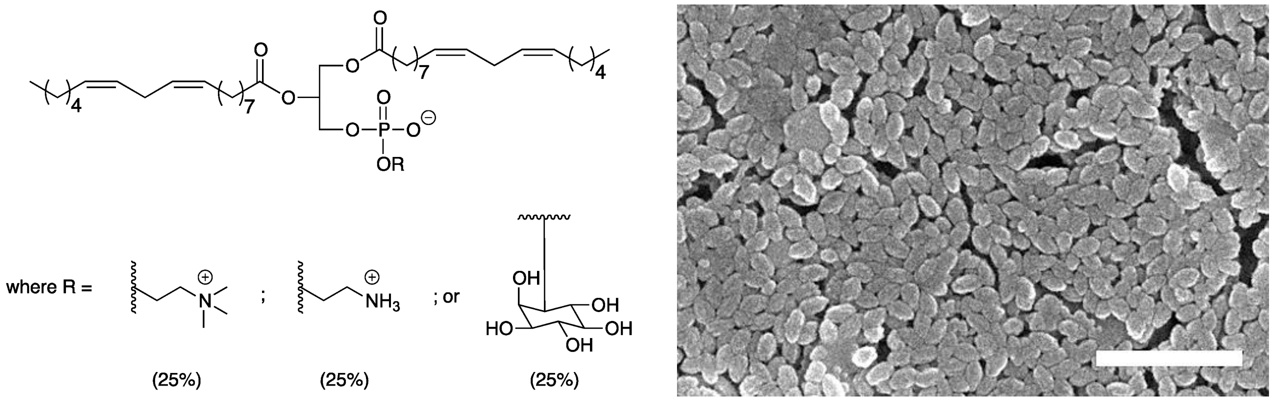

Figure S8. Chemical structure of asolectin and an SEM image of the synthesised NU-901 coated with asolectin, showing the coating does not affect the morphology of NU-901. Scale bar = 500 nm.

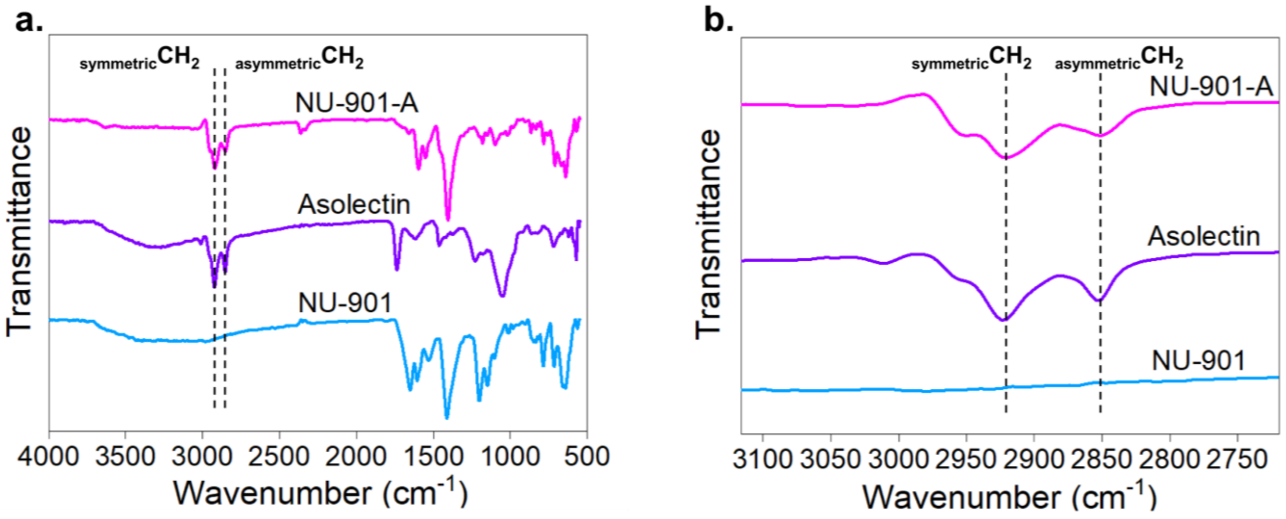

Figure S9. (**a**) Full and (**b**) enlarged FT-IR spectra showing the appearance of new bands at 2837 cm^{-1} and 2854 cm^{-1} attributed to the stretching vibration of $_{symmetric}CH_2$ and $_{asymmetric}CH_2$, respectively, from asolectin.

Table S1. Breakdown of individual components in NU-901-A, NU-901-A-F, Pem@NU-901-A and Pem@NU-901-A-F.

Breakdown of components (percentage of weight wt%)				
	NU-901-A	NU-901-A-F	Pem@NU-901	Pem@NU-901-A-F
NU-901	74.6%	61.8%	74.9%	52.8%
Pemetrexed	-	-	25.0%	15.1%
Asolectin	16.8%	20.1%	-	21.2%
F127	-	16.8%	-	10.1%
Bilayer (asolectin + F127)	-	36.9%	-	31.3%
TOTAL	91.4%	98.7%	99.9%	99.2%

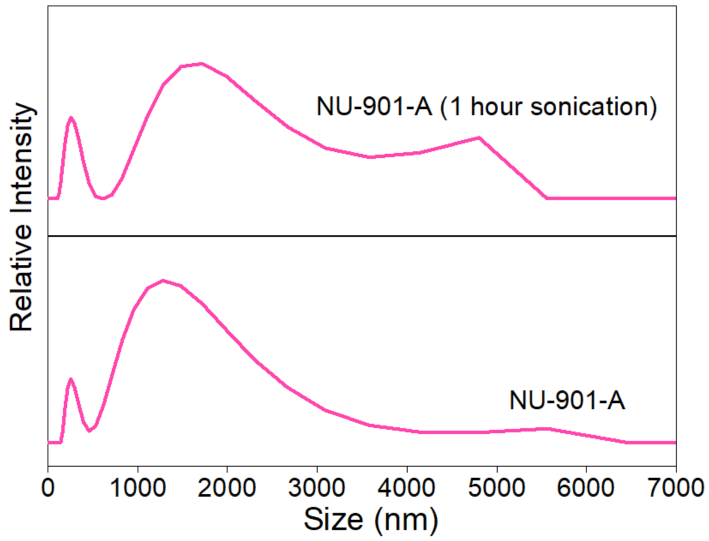

Figure S10. Dynamic light scattering (DLS) spectra of NU-901-A before and after 1-hour sonication at a concentration of 0.1 mg/mL, showing that the particles remained severely aggregated in water even after 1-hour sonication.

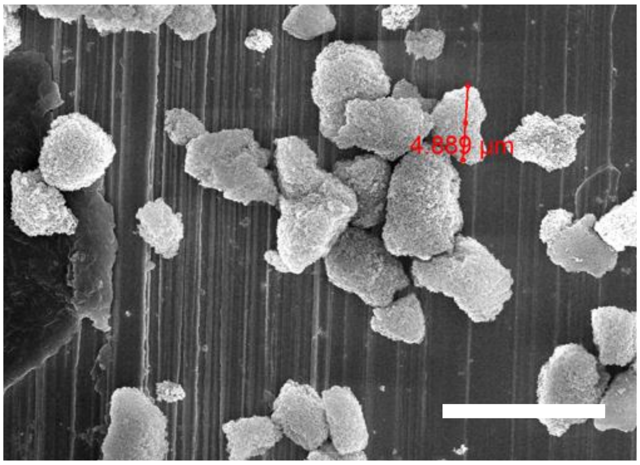

Figure S11. SEM characterization of NU-901-A dispersion in water after 1-hour sonication treatment showing big chunks of agglomerates due to high hydrophobicity of the material. Scale bar = 10 µm.

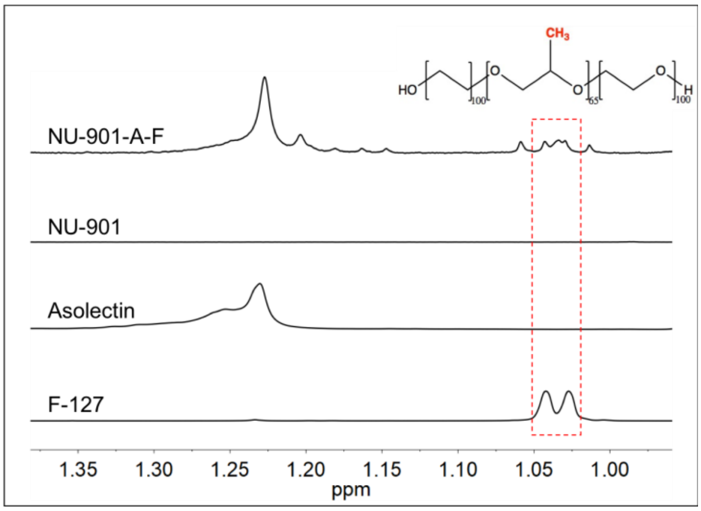

Figure S12. ^{1}H nuclear magnetic resonance (NMR) spectra of F-127, asolectin, NU-901 and NU-901-A-F, confirming the existence of F-127 in NU-901-A-F, as shown in the peaks of CH_3 in PGG block.

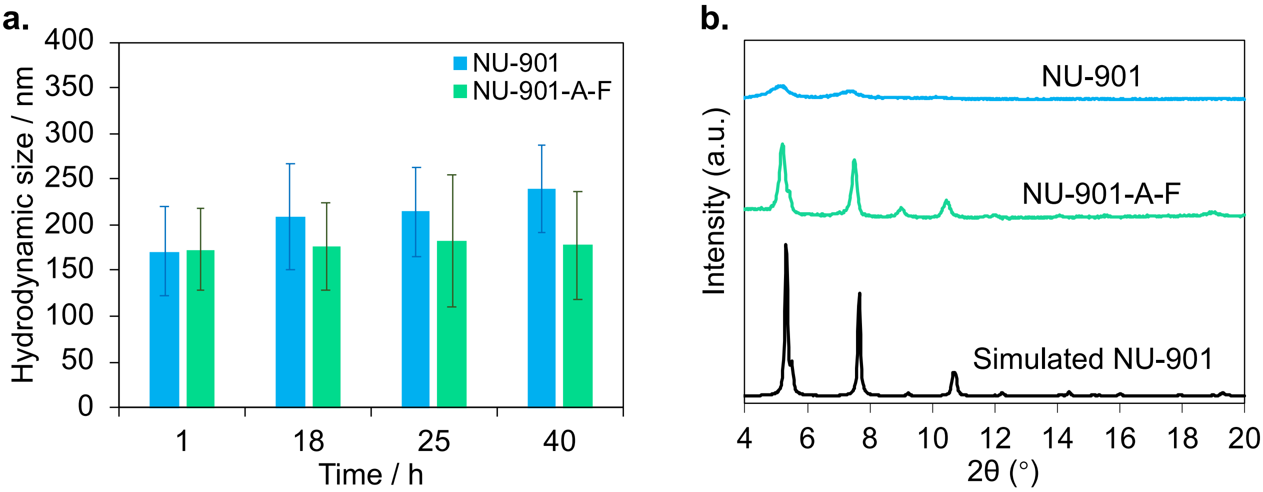

Figure S13. (a) Change in hydrodynamic size of bare and coated NU-901 in MilliQ water at t = 1 h, 18 h, 25 h, and 40 h. **(b)** PXRD patterns of NU-901 and NU-901-A-F after 48-hour dispersion in PBS, with simulated NU-901 as a reference.

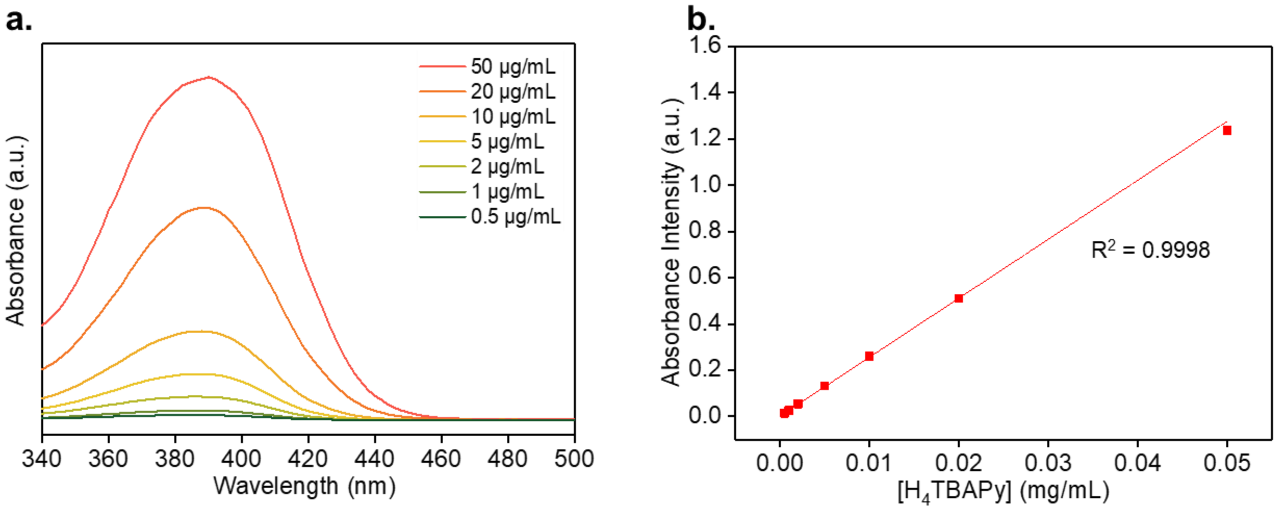

Figure S14. UV-Vis spectra of H4TBAPy linker in PBS (pH = 7) at different concentrations with the corresponded calibration curve.

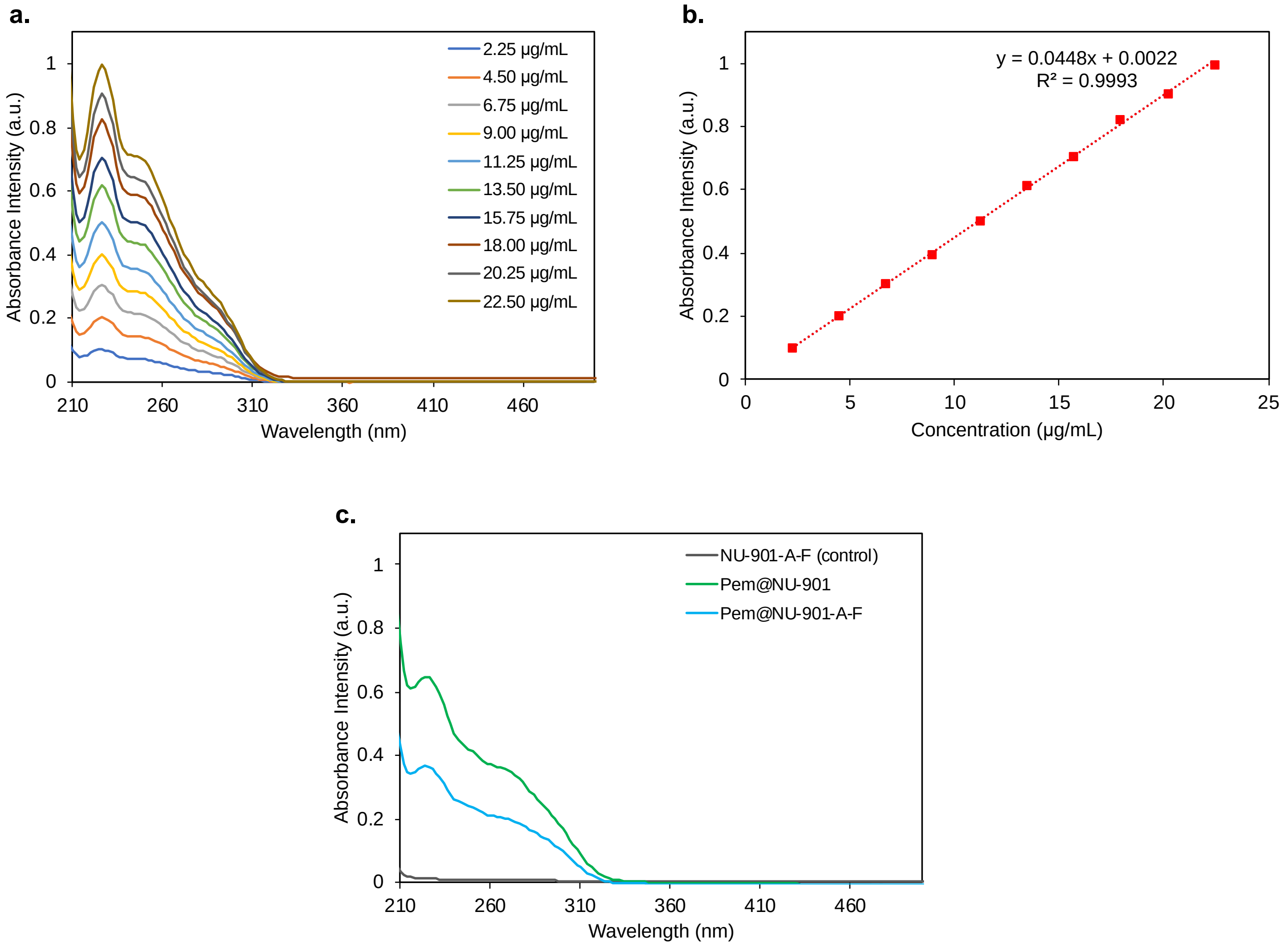

Figure S15. UV-Vis spectra of pemetrexed in water at (**a**) different concentrations with the (**b**) corresponded calibration curve. (**c**) UV-Vis spectra of pemetrexed loading in Pem@NU-901 and Pem@NU-901-A-F, with NU-901 as a negative control showing no interfered intensity to loading measurements.

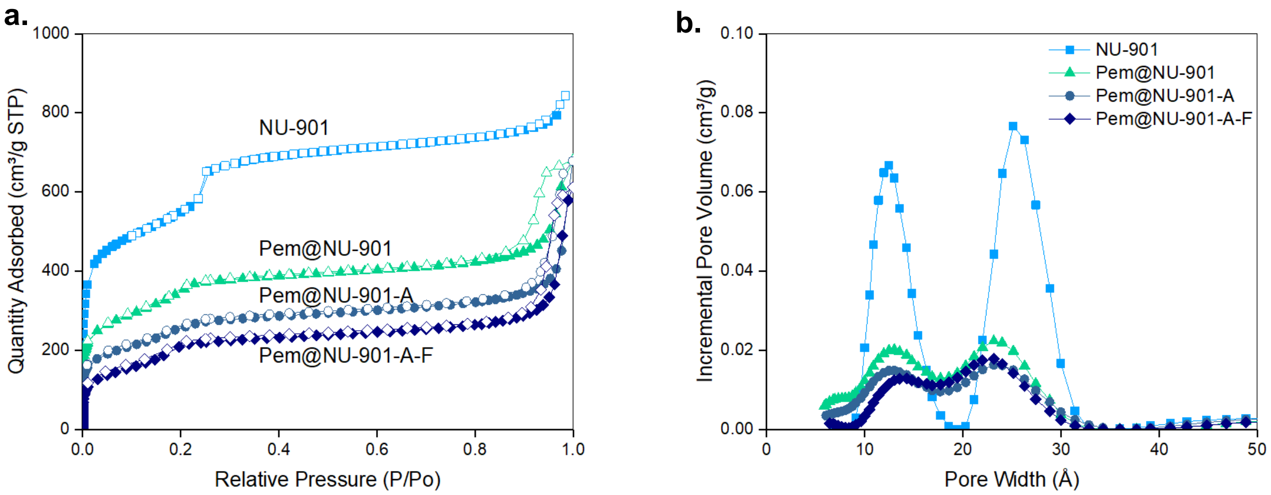

Figure S16. (a) N_2 isotherms of NU-901, Pem@NU-901, Pem@NU-901-A and Pem@NU-901-A-F at 77 K with (b) their corresponded pore size distributions (PSD) using NLDFT method.

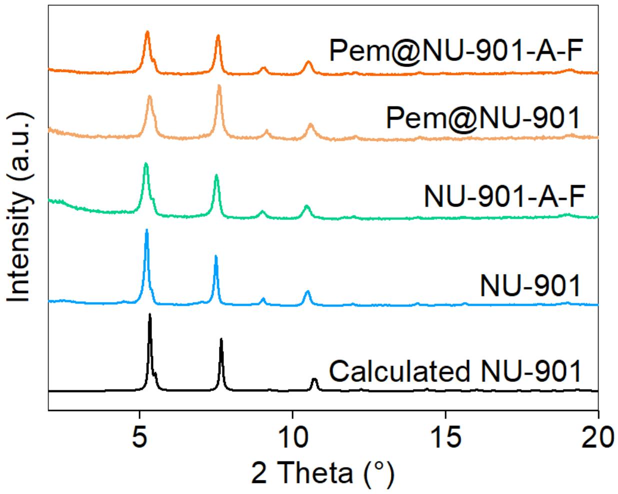

Figure S17. PXRD patterns of NU-901, NU-901-A-F, Pem@NU-901 and Pem@NU-901-A-F with calculated NU-901 as a reference.

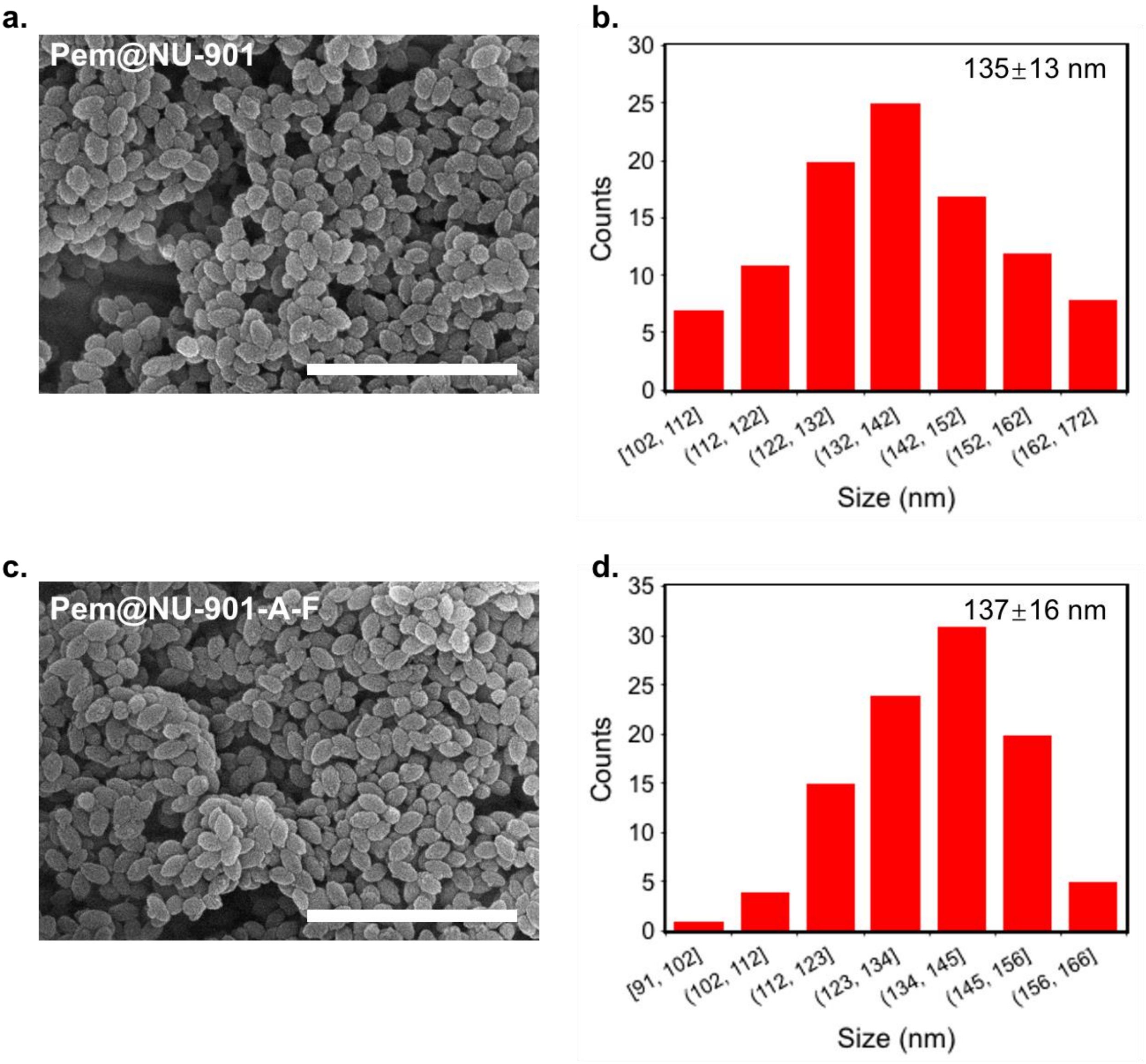

Figure S18. (a) SEM images of Pem@NU-901 with its (b) particle size analysis (n = 100 analysed with Image J); and (**c**) Pem@NU-901-A-F with its (d) particle size analysis (n = 100 analysed with Image J), scale bars = 1 µm.

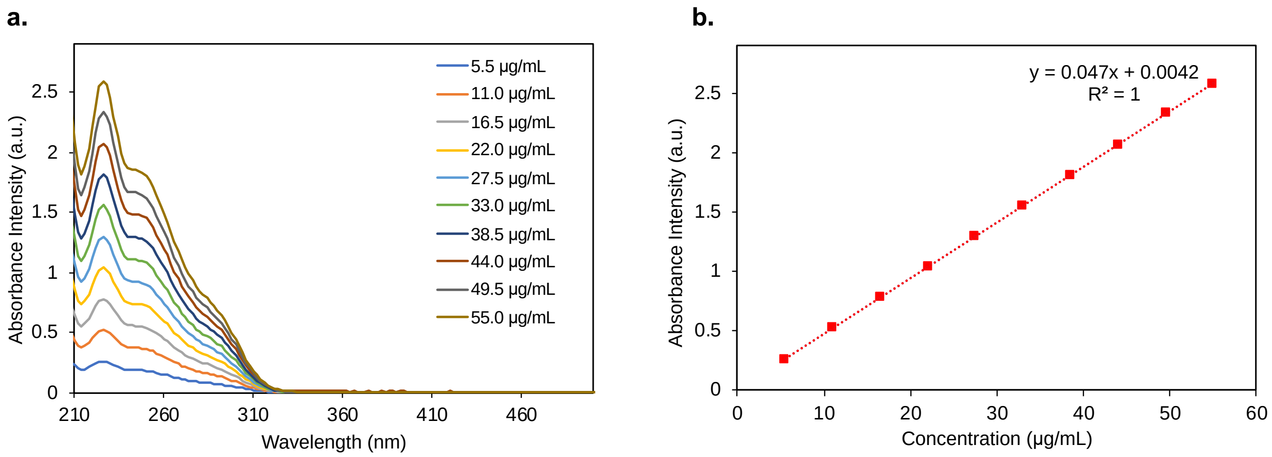

Figure S19. UV-Vis spectra of pemetrexed in PBS (pH = 7.4) at different concentrations with the corresponded calibration curve.

S4. Stability analysis in PBS

Table S1 provides the fitting results of the H_4TBAPy release in PBS from NU-901 and NU-901-A-F. The kinetics of H_4TBAPy degradation from NU-901/NU-901-A-F was adjusted using non-linear regressions to understand its behaviour. The profile was adjusted to a simple hyperbola model for NU-901-NU-901-A-F soaked in PBS[3]:

$$N(wt.\ \%)= \frac{N_{max}\ t}{t_{1/2}+t} \quad [1]$$

where N is the amount of linker released from the solid, N_{max} is the maximum amount released, t is time in hours and $t_{1/2}$ is the time needed to get half of the maximum amount delivered.

Table S1: Fit-curves for the release of H_4TBAPy.

MOF	Equation	R^2
NU-901 in PBS	$N_{H4TBAPy}$(wt%) = 1 t / (10.5 + t) x 100%	0.9752
NU-901-A-F in PBS	no release	1

S5. Analysis of pemetrexed release

Table S2 provides the fitting results of the pemetrexed release in PBS and water from Pem@NU-901 and Pem@NU-901-A-F. The kinetics of pemetrexed delivery from NU-901 and NU-901-A-F were adjusted using non-linear regressions to understand the release behaviour. The only exception is Pem@NU-901-A-F in PBS, where the delivery was adjusted to a hyperbola model considering two different release stages:

$$N(wt.\ \%)= \frac{N_{max\ (1)}\ t}{t_{1/2(1)}+t}+\frac{N_{max\ (2)}\ t}{t_{1/2(2)}+t} \quad [2]$$

where (1) and (2) represent two different stages, N is the amount of linker released from the solid, N_{max} is the maximum amount released, t is time in days and $t_{1/2}$ is the time needed to get half of the maximum amount delivered.

Table S2: Fit-curves for degradation profiles of pemetrexed.

MOF	Equation	R^2
Pem@NU-901 in H_2O	$N_{pemetrexed}$ (wt%) = 0.32 t / (0.002 + t) x 100%	0.9926
Pem@NU-901 in PBS	$N_{pemetrexed}$ (wt%) = 0.1512 t / (0.08 + t) x 100%	0.9898
Pem@NU-901-A-F in H_2O	$N_{pemetrexed}$ (wt%) = 1.03 t / (0.02 + t) x 100%	0.9905
Pem@NU-901-A-F in PBS	$N_{pemetrexed}$ (wt%) = 0.2476 t / (0.28 + t) x 100% + 0.95 t / (2.9 + t) x 100%	0.9917

S6. *In vitro* characterisation of nanoMOFs

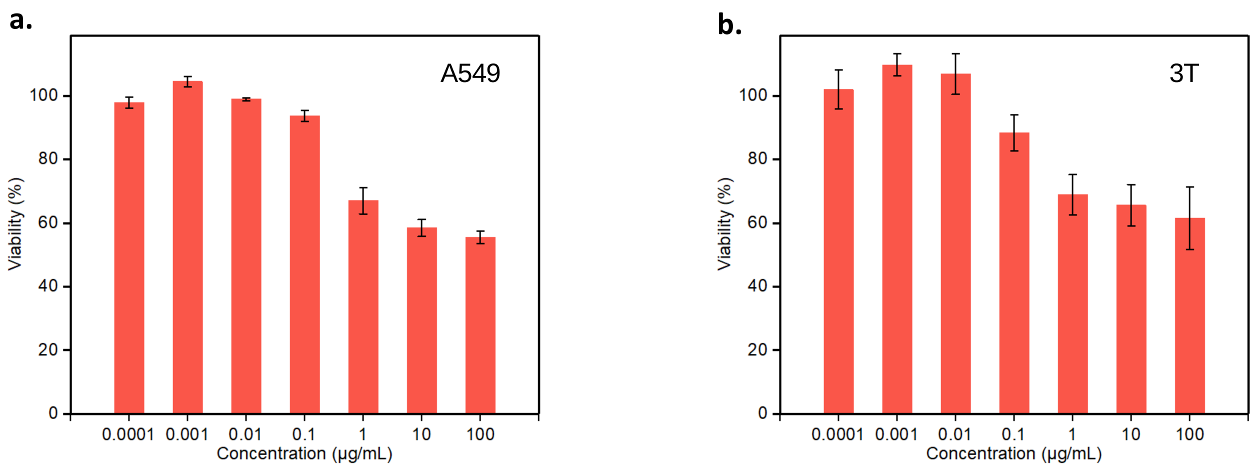

Figure S21. MTS studies. (**a**) A549 and (**b**) 3T cells' viability was measured by MTS assay after 72 hours incubation of pemetrexed. (n = 3 for biological replicates)

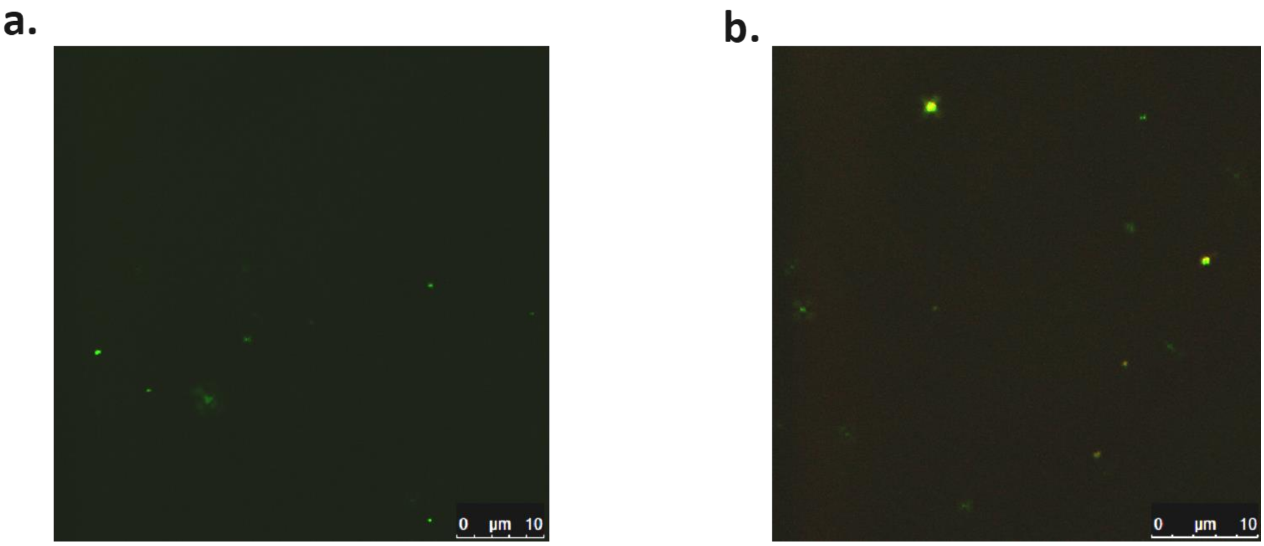

Figure S22. Confocal microscopy image of (**a**) Pem@NU-901 and (**b**) Pem@NU-901-A-F shown as green dim clusters. (scale bar = 10 μm)

S7. BET area calculation using BETSI

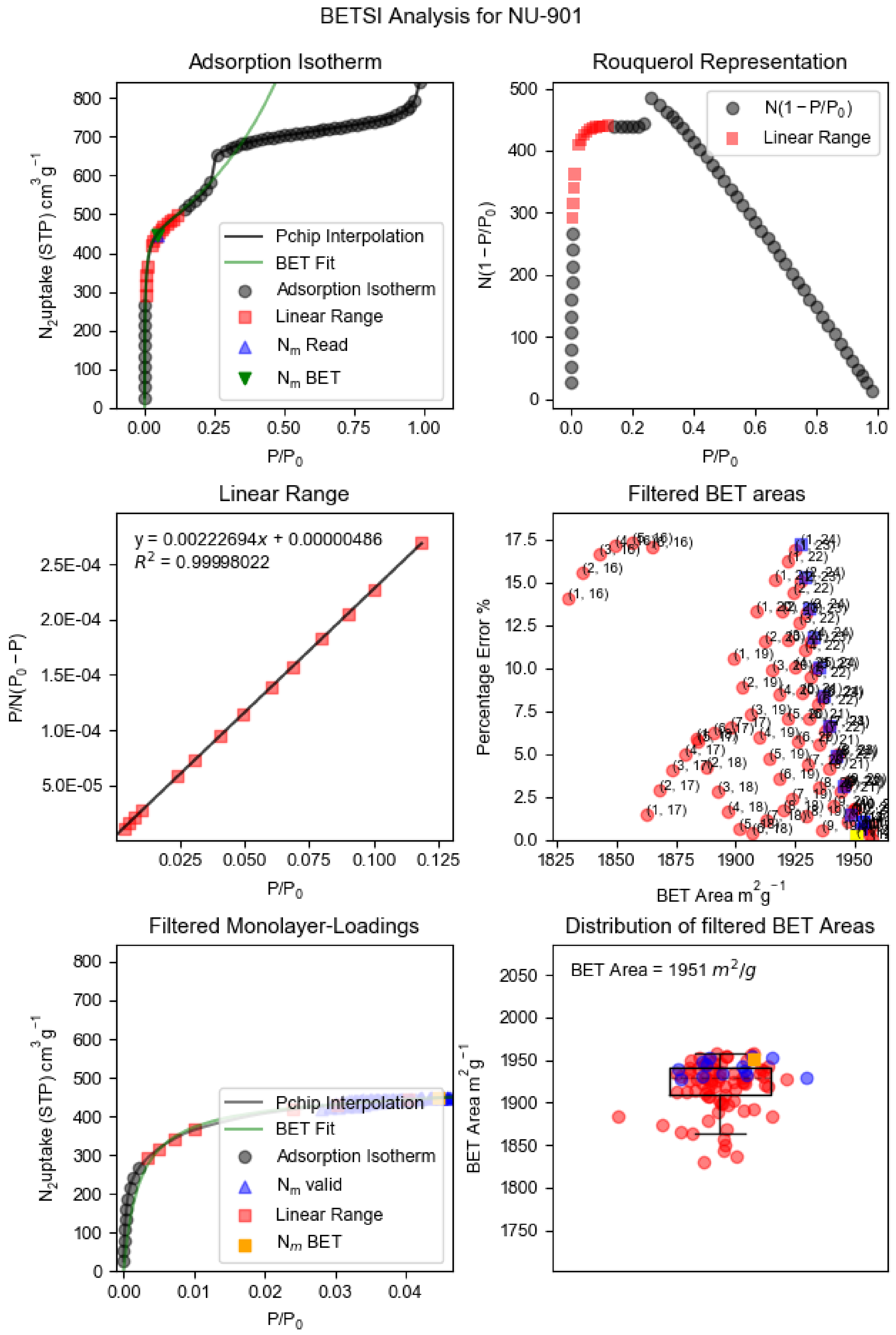

Figure S23. (a) BETSI analysis for NU-901.

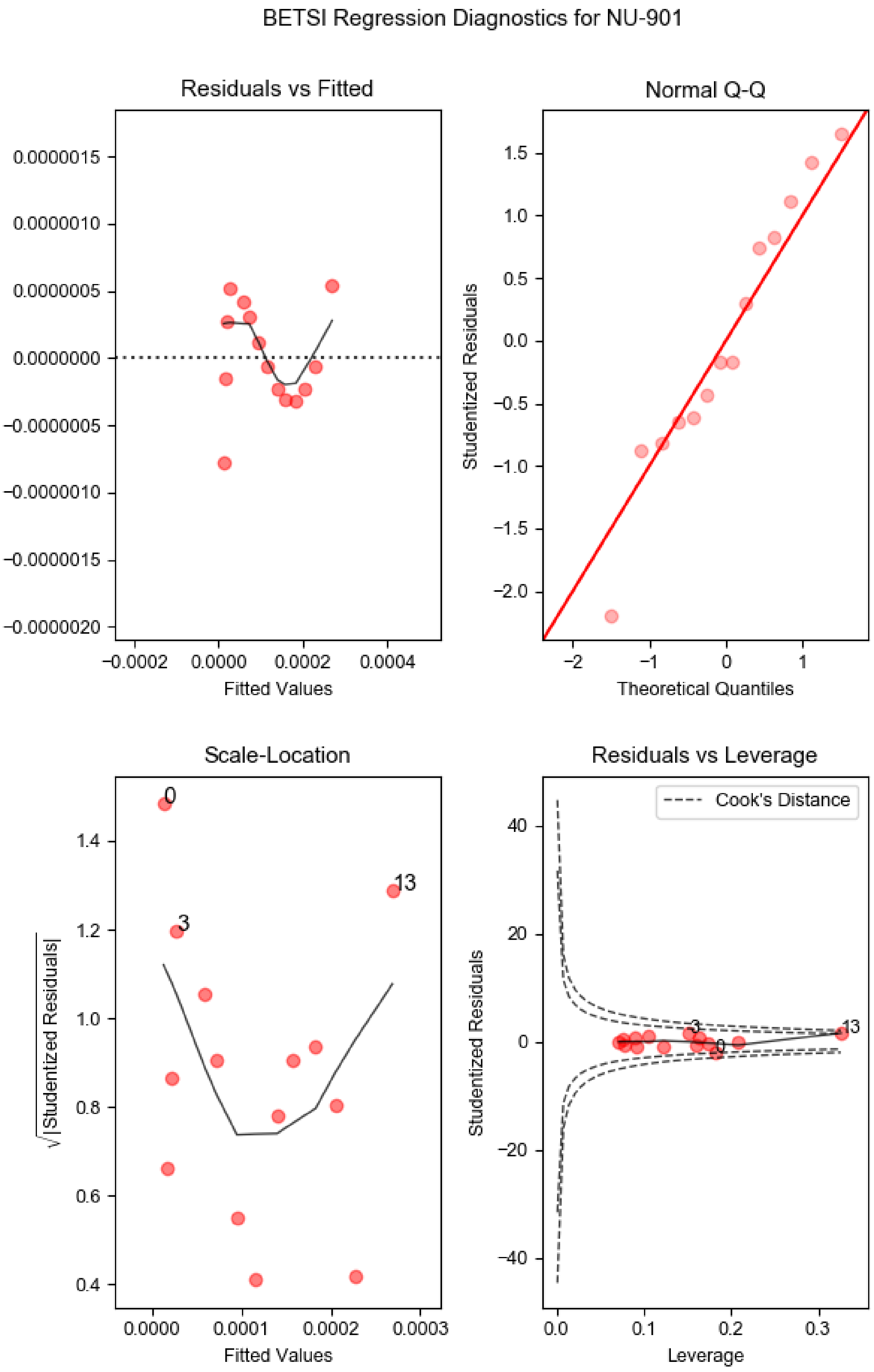

Figure S23. (b) BETSI regression diagnostics for NU-901.

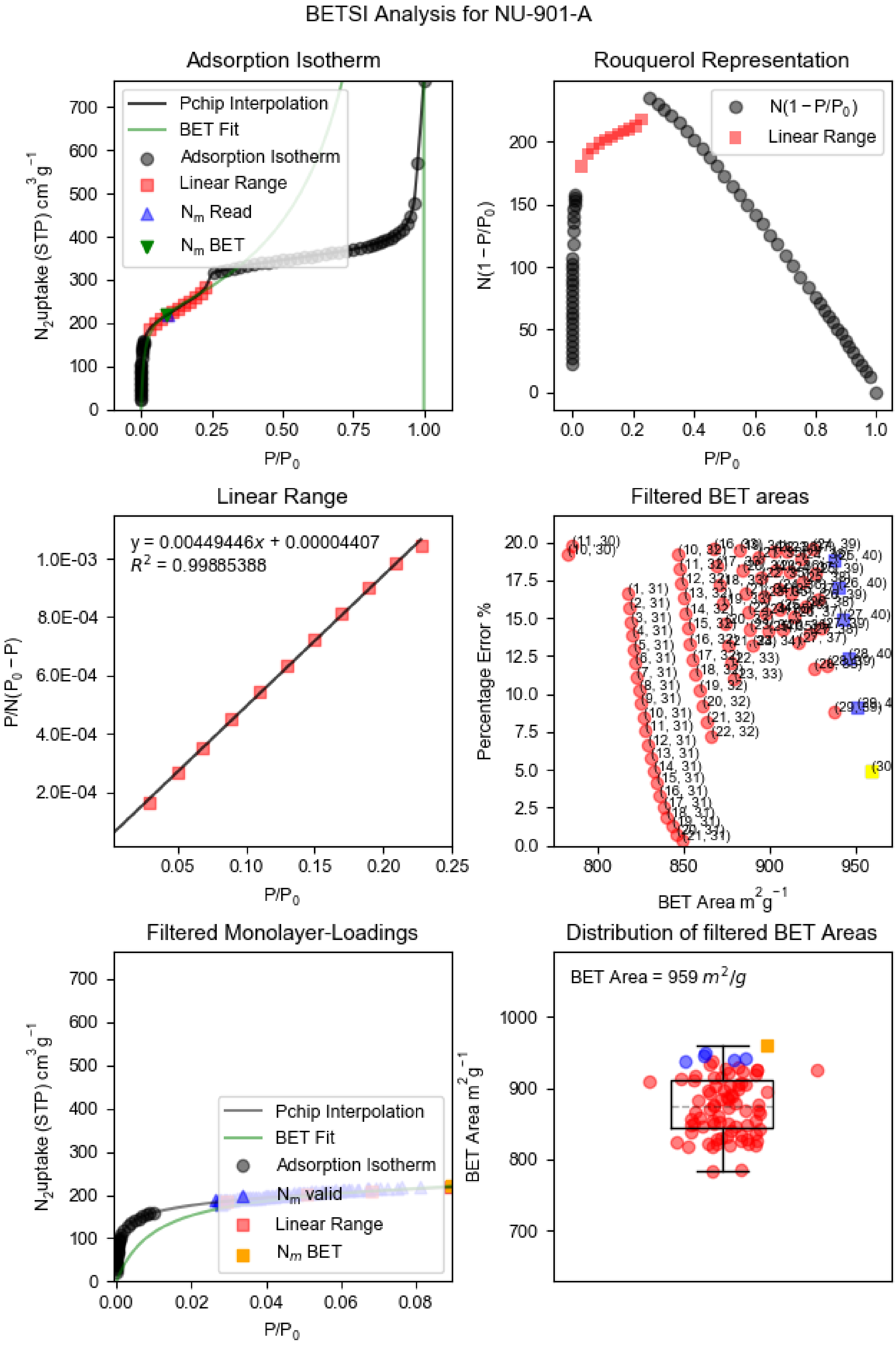

Figure S24. (a) BETSI analysis for NU-901-A.

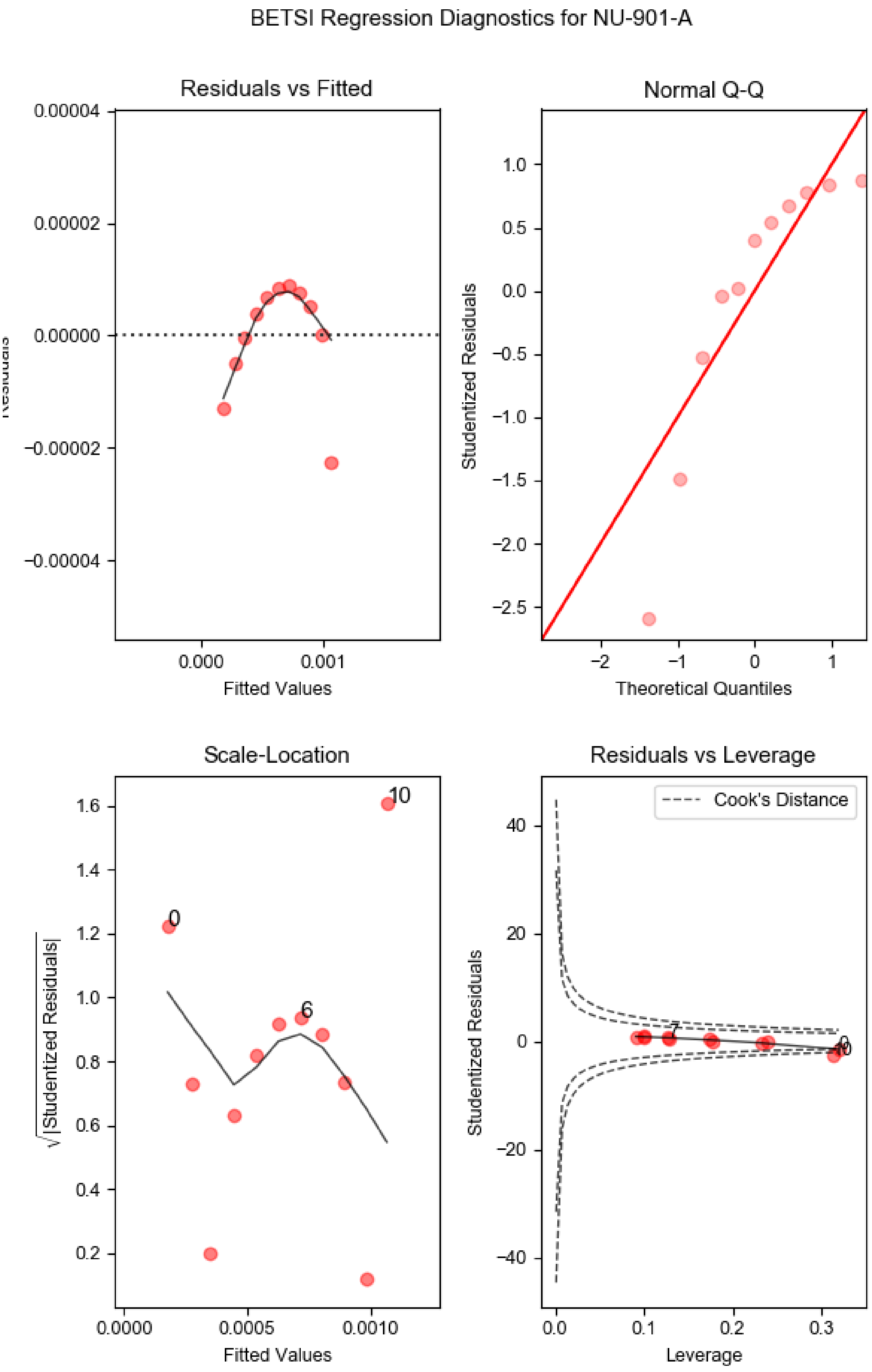

Figure S24. (b) BETSI regression diagnostics for NU-901-A.

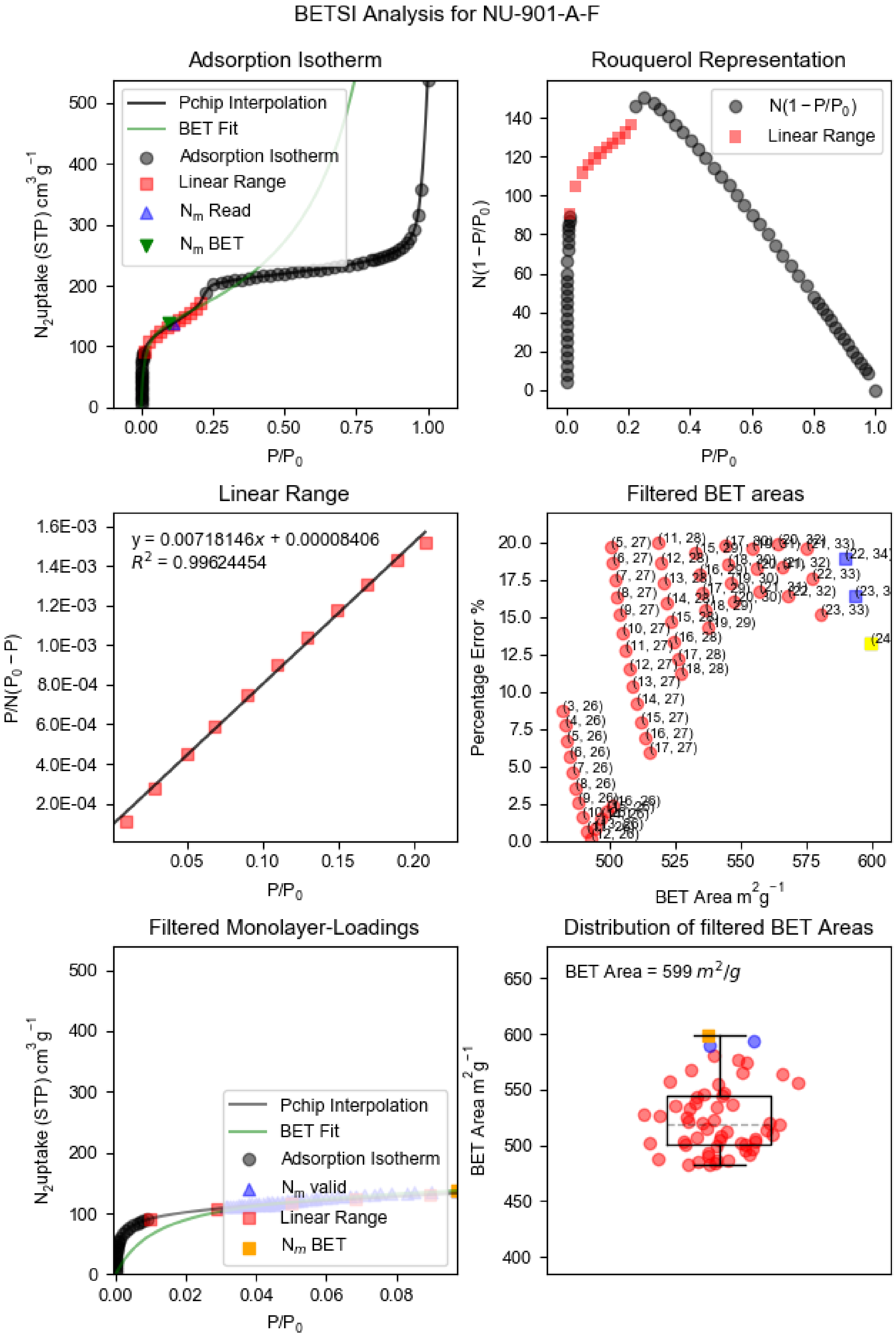

Figure S25. (a) BETSI analysis for NU-901-A-F.

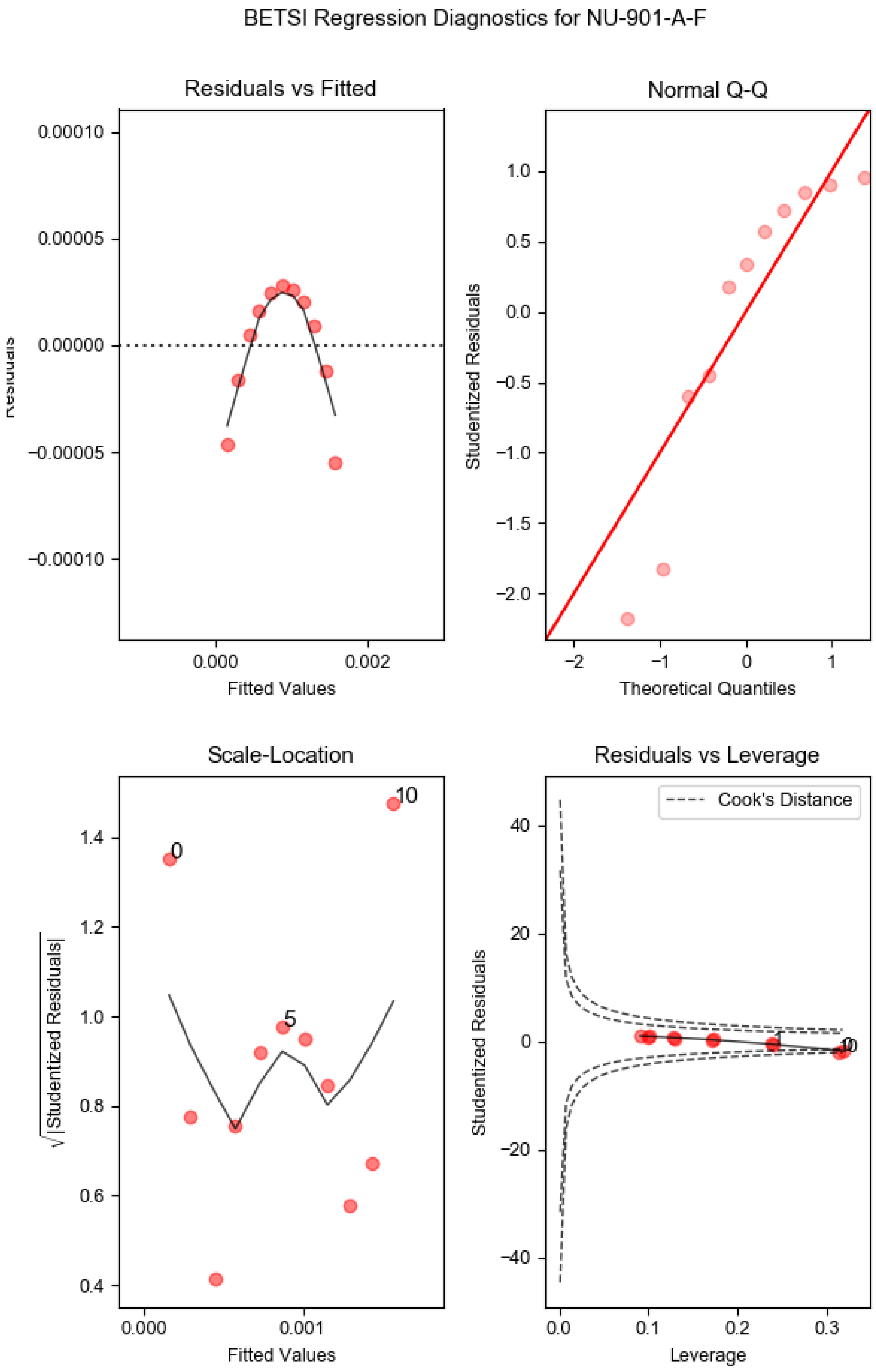

Figure S25. (b) BETSI regression diagnostics for NU-901-A-F.

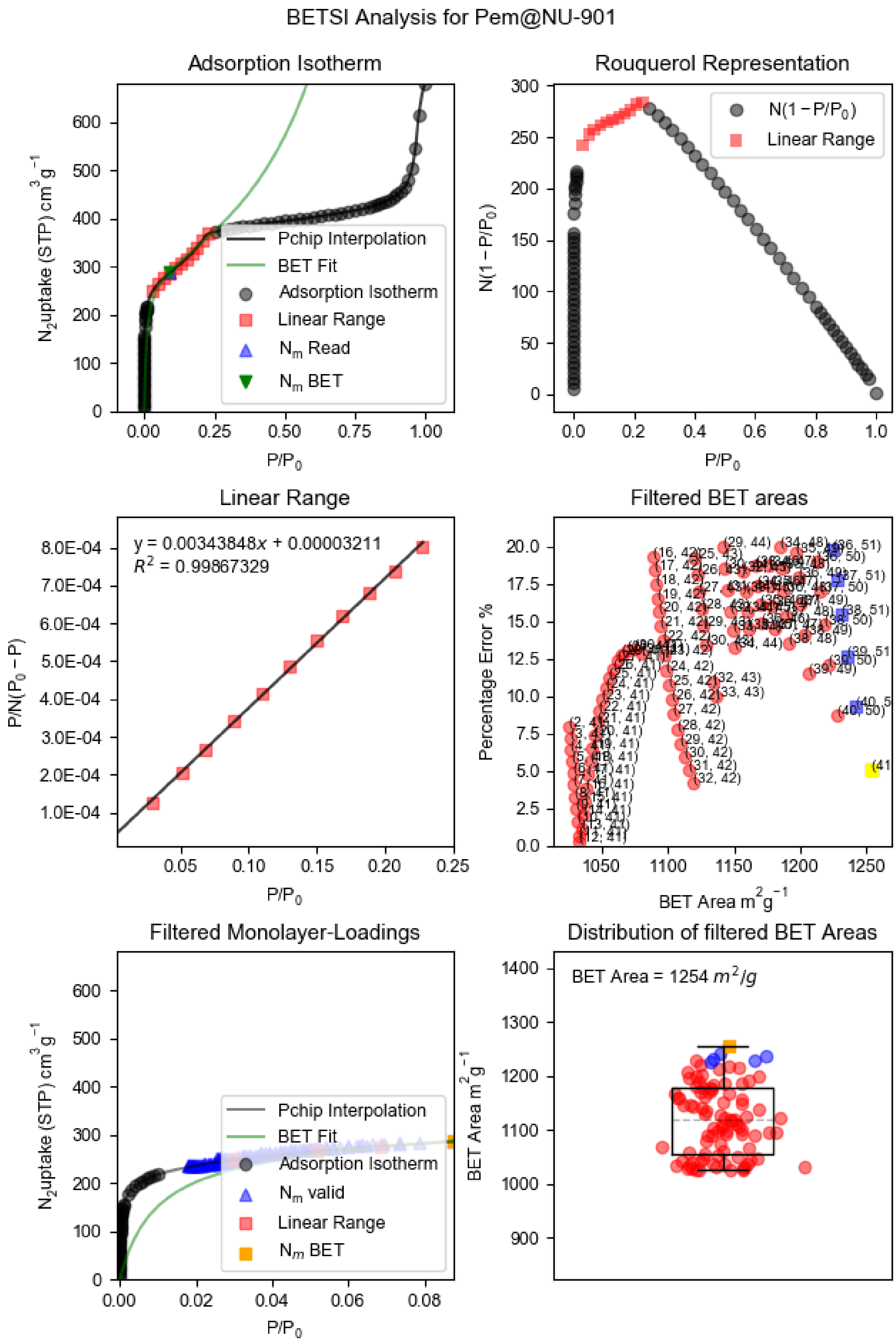

Figure S26. (a) BETSI analysis for Pem@NU-901.

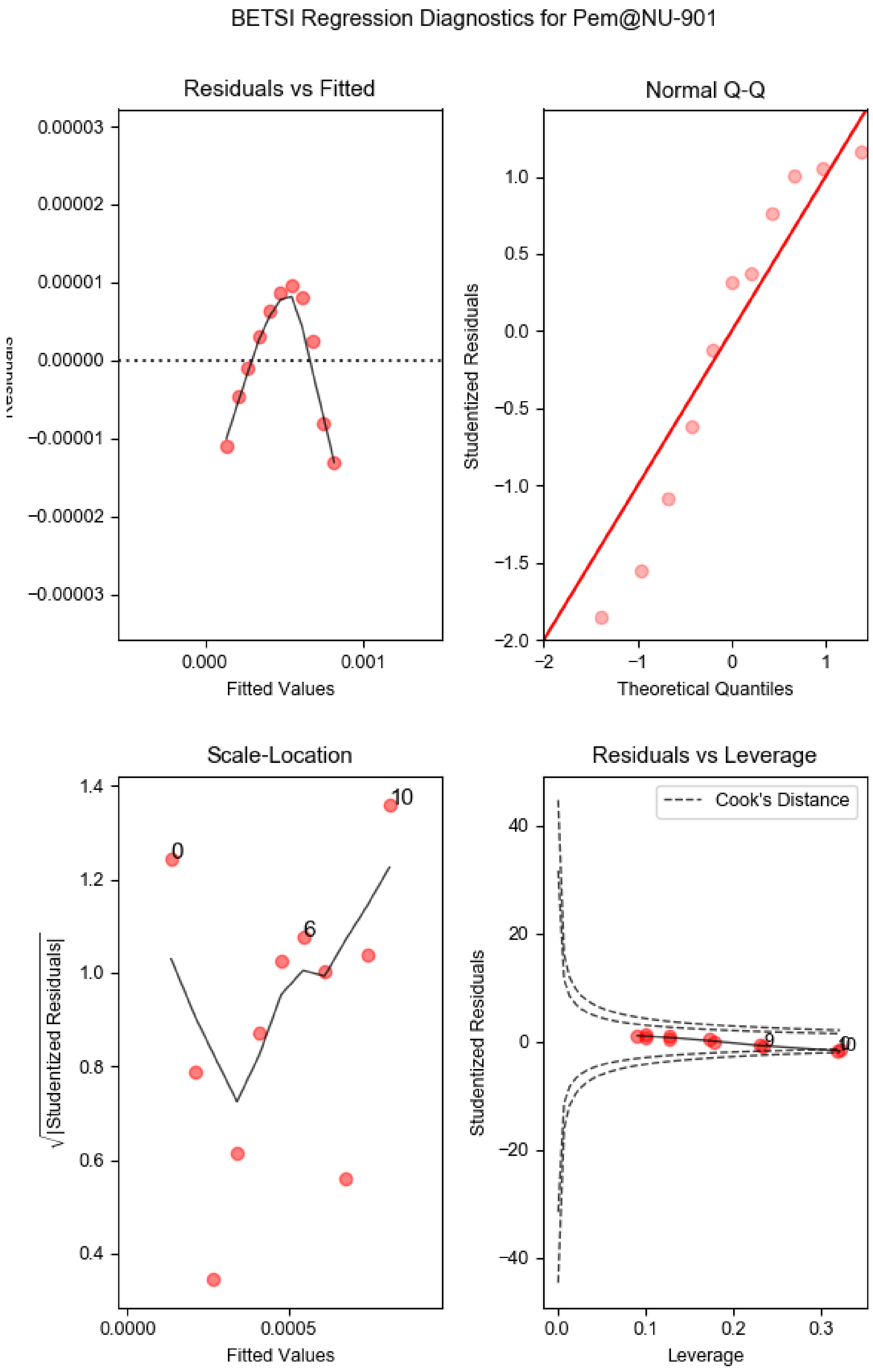

Figure S26. (b) BETSI regression diagnostics for Pem@NU-901.

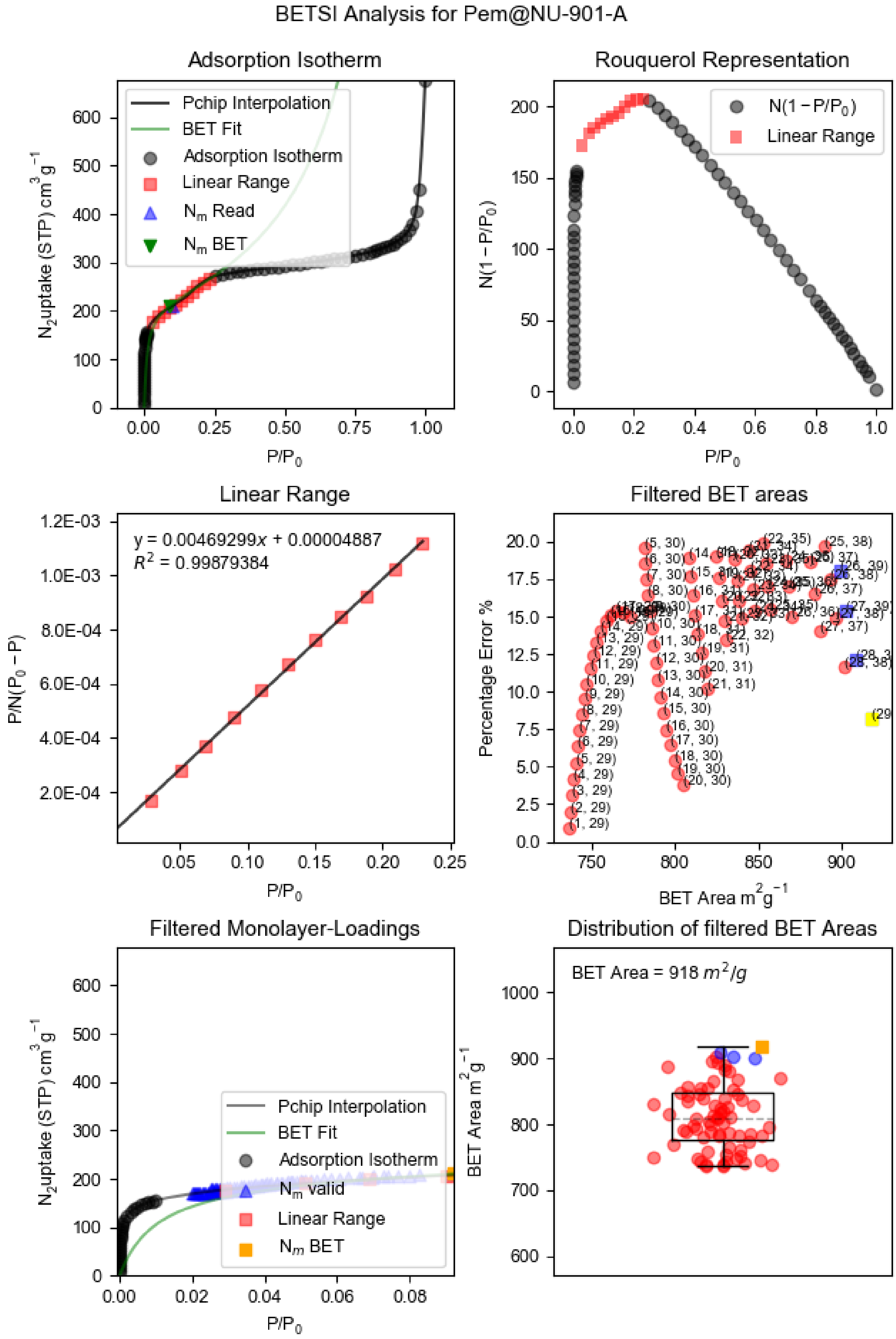

Figure S27. (a) BETSI analysis for Pem@NU-901-A.

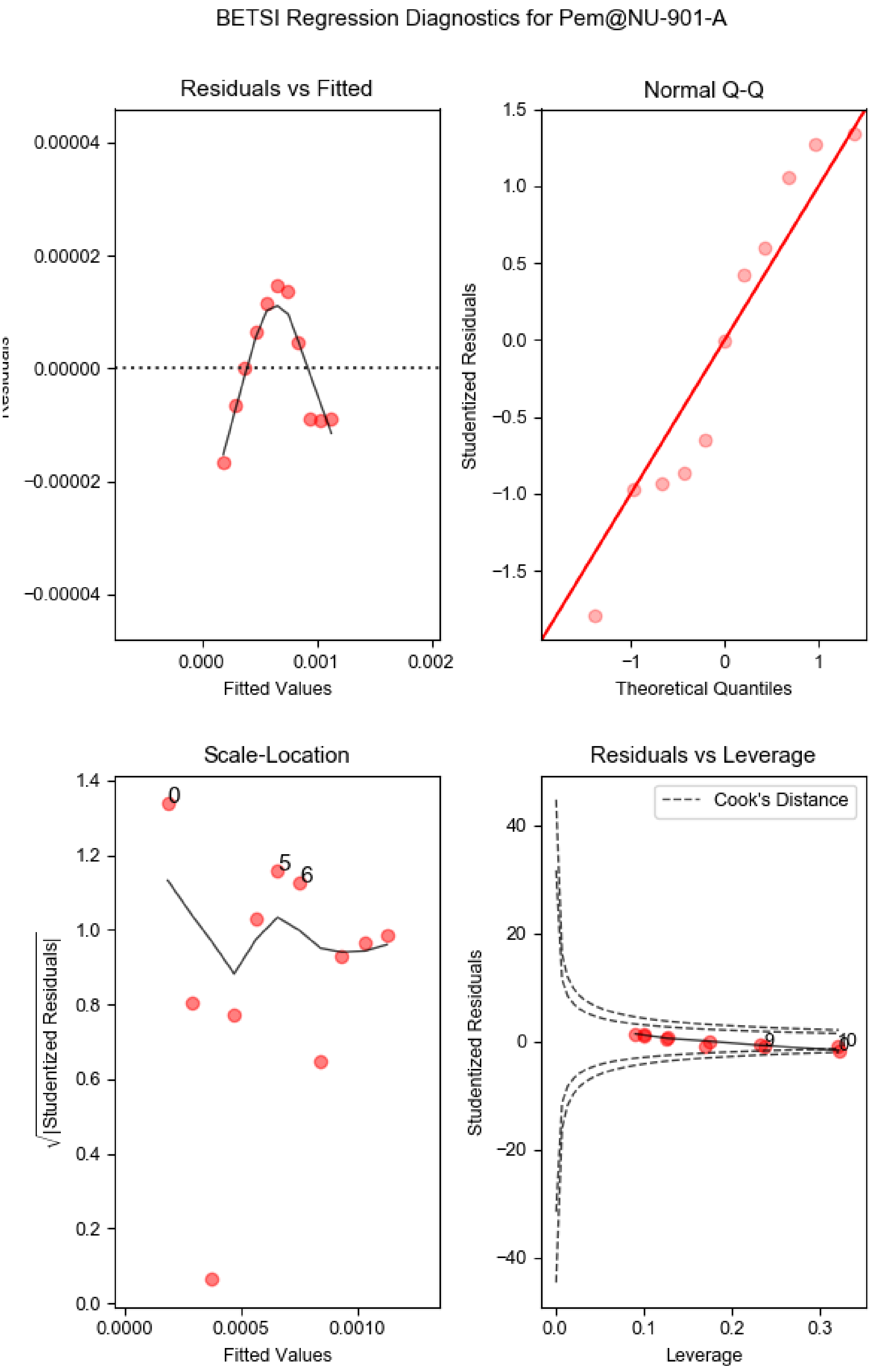

Figure S27. (b) BETSI regression diagnostics for Pem@NU-901-A.

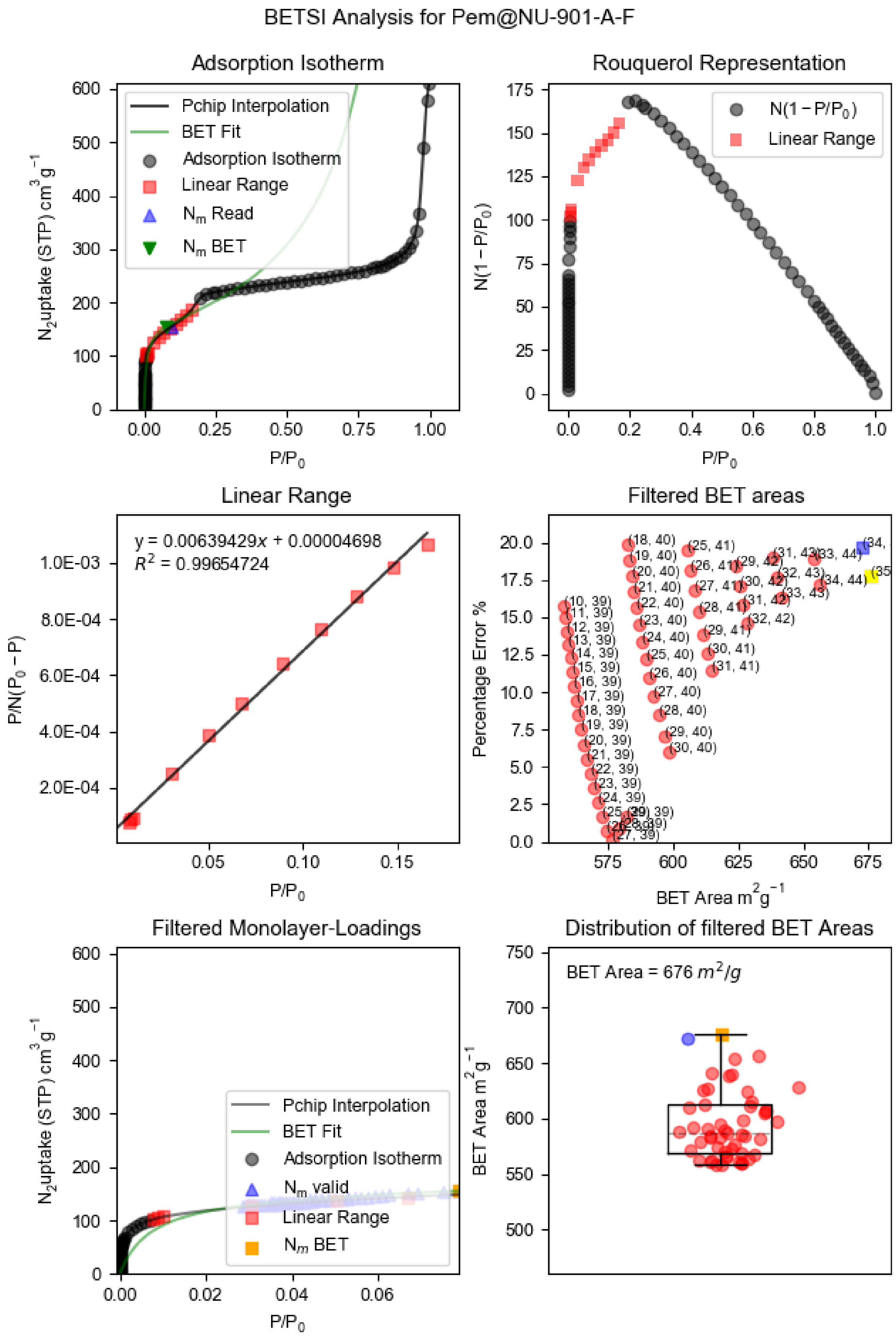

Figure S28. (a) BETSI analysis for Pem@NU-901-A-F.

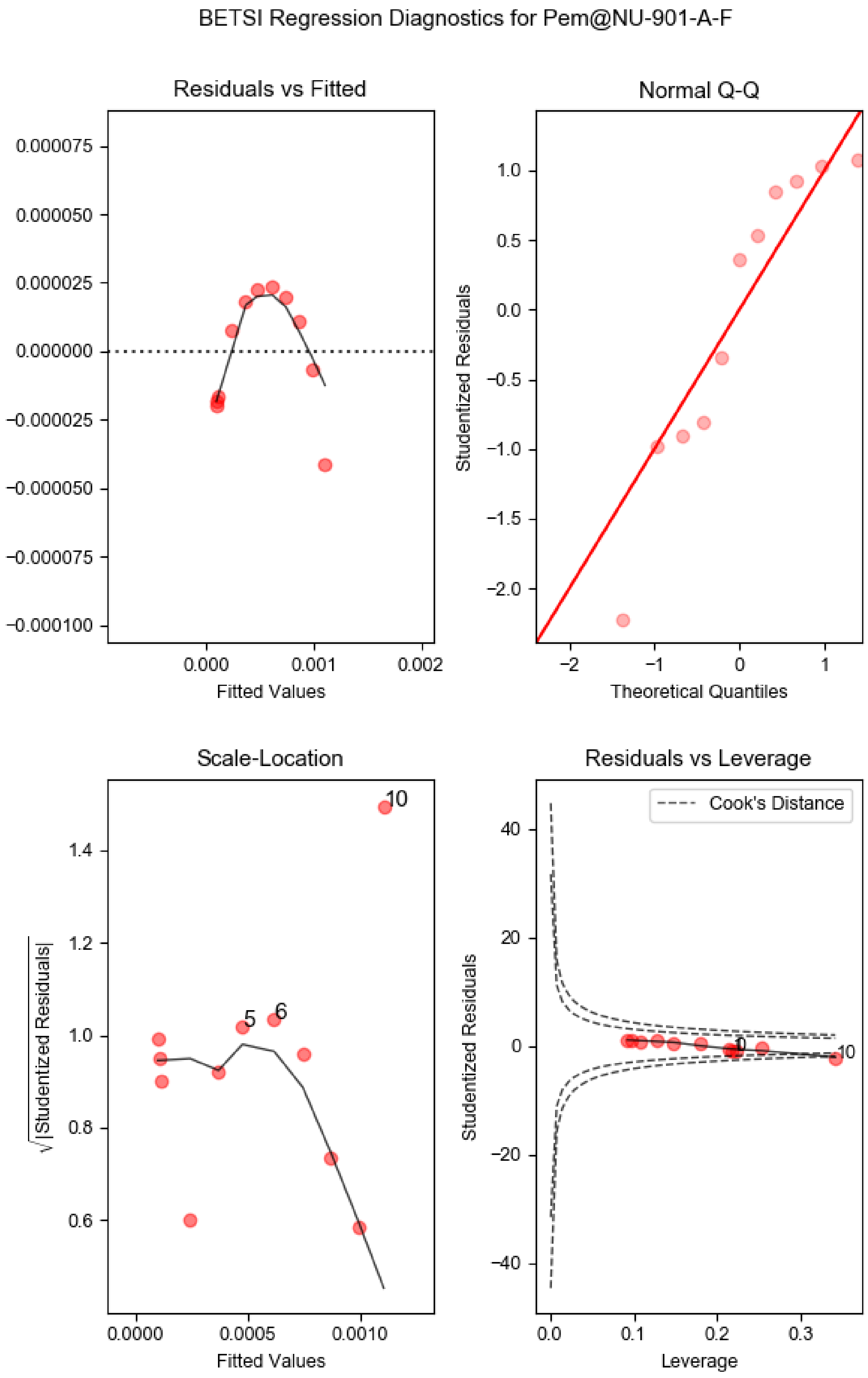

Figure S28. (b) BETSI regression diagnostics for Pem@NU-901-A-F.

S6. References

1. Chernova, T. *et al.* Molecular profiling reveals primary mesothelioma cell lines recapitulate human disease. *Cell Death Differ.* **23**, 1152–1164 (2016).
2. Wang, T. C. *et al.* Scalable synthesis and post-modification of a mesoporous metal-organic framework called NU-1000. *Nat. Protoc.* **11**, 149–162 (2016).
3. Orellana-Tavra, C., Köppen, M., Li, A., Stock, N. & Fairen-Jimenez, D. Biocompatible, Crystalline, and Amorphous Bismuth-Based Metal-Organic Frameworks for Drug Delivery. *ACS Appl. Mater. Interfaces* **12**, 5633–5641 (2020).
